# Does Hazardous-Waste Testing Follow Technical Guidance, Thus Help Protect Environmental Justice and Health?

**DOI:** 10.3390/ijerph19137679

**Published:** 2022-06-23

**Authors:** Kristin Shrader-Frechette

**Affiliations:** Department of Biological Sciences, University of Notre Dame, Notre Dame, IN 46556, USA; kshrader@nd.edu

**Keywords:** environmental justice (EJ), hazardous waste, soil-gas testing, Trammell Crow (TC), trichloroethylene (TCE), vapor intrusion, volatile organic compound (VOC), weight of evidence method (WoE)

## Abstract

Does representative hazardous-waste-site testing tend to follow or to violate government technical guidance? This is an important question, because following such guidance promotes reliable risk analysis, adequate remediation, and environmental-justice and -health protection. Yet only government documents typically address this question, usually only when it is too late, when citizens have already exhibited health harm, allegedly from living or working near current/former hazardous-waste sites. Because no systematic, representative, scientific analyses have answered the preceding question, this article begins to investigate it by posing a narrower part of the question: Does representative US testing of volatile-organic-compound (VOC) waste sites tend to follow or to violate government technical requirements? The article (i) outlines US/state-government technical guidance for VOC testing; (ii) develops criteria for discovering representative US cases of VOC testing; (iii) uses the dominant US Environmental Protection Agency method to assess whether these representative cases follow such guidance; (iv) employs the results of (iii) to begin to answer the preceding question; then (v) discusses the degree to which, if any, these results suggest threats to environmental health or justice. Our initial, but representative, results show that almost all US VOC-waste-site testing (that we investigated) violates government technical requirements and systematically underestimates risks, and this may help justify less expensive, potentially health-threatening cleanups, mostly in environmental justice communities. We outline needed future research and suggest two strategies to promote following government technical guidance for hazardous-waste testing.

## 1. Introduction

Early in 2018, the 40,000-member African-American community living in homes built on San Francisco’s former Hunter’s Point hazardous-waste site filed a $27 billion class-action lawsuit against the contractor Tetra Tech. The suit accused the corporation of falsifying sampling and, as a result, causing community members’ environmental injustice, cancer, and death [1]. A year later, the US Department of Justice also sued Tetra Tech for falsifying soil samples and conducting hundreds of millions of dollars of fraudulent waste-site testing and cleanup [2]. In 2019, the San Francisco Department of Public Health discovered that Hunter’s Point residents had elevated rates of 13 different cancers, all of which can be caused by site contaminants [3].

How typical of other hazardous-waste sites are the alleged Hunter’s Point testing flaws and environmental-health problems? For at least four reasons, public-health officials are unsure. *First*, although many governments monitor hazardous-waste generators, the reliability of site testing/cleanup is mainly unknown; scientific databases reveal no systematic, representative, third-party studies that empirically assess hazardous-waste testing. *Second*, as Section S2.2 of Supplementary Materials S2 explains, at least in the United States, most hazardous-waste testing is privatized; as a result, testing documents tend to be unavailable. *Third*, because of this privatization, government typically does not independently audit hazardous-waste sampling (see Section S2.2 of Supplementary Materials S2). *Fourth*, unless potentially affected communities provide evidence for possible waste-caused health harm, as at Hunter’s Point, government usually conducts no oversight waste-site testing. Yet by the time such oversight testing occurs, environmental disease or death generally have already occurred. Thus there is an important environmental-health data gap about the adequacy of hazardous-waste testing/cleanup and resulting harm, if any.

This article is a small step in beginning to address the preceding data gap. It investigates both whether current, representative, hazardous-waste-site testing follows regulatory guidance for assessing volatile organic compounds (VOCs) and the main potential environmental-health and environmental-justice consequences, if any, of following, versus violating, VOC-testing guidance.

### 1.1. Background

The question about the prevalence of flawed waste-site testing arose for the author in spring 2018, when members of the mostly poor minority community living near a hazardous-waste site—the former Naval Ordnance Testing Station, Pasadena, California, NOTSPA (see Section 1.2 below)—requested a pro bono technical consultation. They asked University of Notre Dame science and public-health faculty members, associated with the Notre Dame Center for Environmental Justice and Children’s Health, to assess (1) potential health and environmental-justice risks associated with NOTSPA and (2) the scientific reliability and health protectiveness of the testing/cleanup documents of NOTSPA’s developer, Trammell Crow (TC), the largest US/global commercial developer [4]. Nearby residents were concerned because they said the developer planned to employ land-use restrictions at NOTSPA (see Supplementary Materials S1, Figures S7–S9), instead of remediation (see Section 4.1.3), yet to build onsite apartments for families with children. This article is part of the work product resulting from the requested pro bono consultation. Besides assessing (1)–(2), this work product also included an investigation of the regulatory-guidance, testing documents, and environmental-justice/-health characteristics associated with a representative sample of VOC hazardous-waste sites.

Because NOTSPA is both a VOC hazardous-waste site and is surrounded by a typical environmental justice community (see Section 1.2 below), it illustrates the health and justice importance of following regulatory guidance for testing. In the period 1943–1976, NOTSPA was part of the US Navy’s “largest and most complete” weapons research and development center; it tested and manufactured anti-aircraft weapons, torpedoes, and missiles, including Polaris nuclear missiles [5]. After NOTSPA activities were moved to San Diego, the Navy sealed site VOC contaminants by asphalt-capping NOTSPA. In 1976, the Navy sold the unremediated site to a private storage and rental facility that now leases its 29 old World-War-II-era buildings. Although scores of site transformers and the incoming-supplies and outgoing-weapons rail lines have been removed, NOTSPA looks otherwise almost exactly as it did in 1943, with a weapons-manufacturing plant; combustion labs; massive, multistory weapons-test tanks; hazardous-chemicals storage rooms, Quonset huts, etc., many with floor drains into the soil [5] (pp. 2, 6, 13); see Figures S7–S9 in Supplementary Materials S1.

The NOTSPA developer’s testing documents reveal that chlorinated solvents—particularly the VOCs trichloroethylene (TCE), perchloroethylene (PCE), carbon tetrachloride (CT), and dibromochloromethane (DBCM), the four highest site risks—are ubiquitous in site soil, at concentrations up to nearly one million times above allowed levels. For decades, state regulators have warned of NOTSPA vapor intrusion (VI), migration of subsurface VOC carcinogens/neurotoxins into above-ground buildings. As a result, regulators filed an Imminent and Substantial Endangerment Order because of VI threats to site renters and nearby residents. However, the site has never been cleaned up (see Supplementary Materials S2, Section S2.1).

### 1.2. Environmental-Justice (EJ) Communities Surround Hazardous-Waste Sites

Like NOTSPA, hazardous-waste sites (and whether their testing/cleanup follows regulatory guidance) affect environmental-justice (EJ) communities because most toxic-waste sites throughout the world are surrounded by EJ communities. Thus, if pollution is not properly tested/remediated, EJ communities bear the highest risks. That is, statistically, the closer one lives to a hazardous-waste site (or another heavily polluted, risky facility), the more likely that one is a member of an EJ community. EJ communities are those whose members, as compared to the rest of the local population, tend to bear higher levels of pollutants, have poorer health, be members of minority groups, have lower socioeconomic status, be unemployed, have lower levels of education, or have several of the preceding characteristics [6].

The California state government’s CalEnviroScreen data show that the census tract (in which NOTSPA is located) is a typical EJ community. Regarding unemployment, in pre-COVID 2019, the average California unemployment rate was 4%, but the NOTSPA census-tract unemployment rate was 600% higher [7]. Regarding education, an average of 19% of Californians have not finished high school, but the NOTSPA census-tract low-education rate is 200% higher [7]. Regarding minorities, 35% of NOTSPA census-tract residents are “linguistically isolated”, non-English speakers [8]. Regarding poorer health, the California average for low-birthweight newborns is 7%, but for the NOTSPA census tract, this rate is 350% higher [7]. Likewise, the average percentage of California asthmatics is 8.5%, but in the NOTSPA census tract, the asthma rate is 271% higher [8]. Regarding pollution levels, the government’s CalEnviroScreen multiple-pollutant indicators show that for the average Californian, 50% of state census tracts pose worse health/pollutant risks than where they live; however, for NOTSPA census-tract residents, only 7% of state census tracts are more polluted than where they live [7].

Unsurprisingly, some of the health risks in the NOTSPA census tract may be at least partly attributable to NOTSPA-site pollutants. For instance, the disproportionately high incidence of low-birthweight infants in this census tract (350% higher than the state average [7]) may arise partly from exposure to the soil-gas/airborne VOC, TCE [9], one of the four main NOTSPA contaminants [10].

### 1.3. Why Most Hazardous-Waste Sites Are Not Remediated

Besides illustrating the importance of reliable waste-site testing and its potential environmental health/justice characteristics, NOTSPA also illustrates why most US hazardous sites have not been remediated. Although NOTSPA must follow US CERCLA-cleanup requirements, like most waste sites, it has not been remediated. Of 1714 US CERCLA/superfund sites, only 387 (23%) have been remediated [11,12,13]. Of up to 1,000,000 US brownfields [14], only 19% (192,230/1,000,000) have been remediated [15]; see Supplementary Materials S2, Section S2.1, including for US CERCLA and brownfields definitions. Why is there so little remediation?

One reason is that some decisionmakers have decided that immediate hazardous-site reuse has economic benefits that “outweigh…significant and unavoidable” hazardous impacts; as a result, roughly 9000 brownfields are “ready for reuse,” but not remediated [15,16,17] (p. 18). Another reason is that when cleanups are expensive, responsible parties often use legal maneuvers to avoid/reduce them. Amid such legal maneuvers, government typically has insufficient resources either to compel or to conduct remediation of the many US hazardous sites. 

Other reasons for incomplete or failed waste cleanup are fraud, inadequate government information, and privatized [15] (including “voluntary”) remediation that typically has minimal or no government oversight [18,19]; see Supplementary Materials S2 (Sections S2.1 and S2.2). Like NOTSPA, nearly all US toxic-site cleanups have been privatized because of inadequate government remediation funding. However, states vary in the levels to which their cleanups are privatized, that is, the degrees to which the states outsource (to for-profit redevelopers) remediation functions like permitting/testing/cleanup/ oversight/enforcement.

Most states argue that privatized waste-testing/cleanup saves money and increases redevelopment; however, most public-health and legal experts caution that financial motives typically drive privatized remediation and that corporations routinely “conduct a partial investigation or remediation without penalty” [18]. As a result, EJ communities typically face the greatest waste-site health threats from privatized testing/cleanup [18]; see Supplementary Materials S2, Sections S2.2 and S2.3.

### 1.4. Study Objective and Question

This study is **significant** because, to our knowledge, it is part of the first set of independent (non-government, non-interested party) analyses to begin systematically investigating hazardous-site VOC testing before potential health harm appears. Our study **objective** is to provide a preliminary answer to the **question**: Does representative testing at US subsurface VOC-contaminated sites tend to follow or to violate the “requirements” of the US regulatory technical guidance for VOCs? (see paragraphs below and Supplementary Materials S2, Section S2.7 for why/how government technical guidance can impose requirements.)

This question addresses the testing of emergent VI threats, partly because up to 250,000 US hazardous sites, even supposedly remediated sites, likely require VI testing and remediation [20]. In addition, the pro bono EJ-community consultation, requested of our university center, requires the examination of VOC-waste sites. 

Analysis of this question also focuses on regulatory-guidance “requirements” for testing, for at least three reasons. A *first* reason is that government technical guidance specifically uses the term “requirements;” it mandates that “soil-[VOC] data for evaluating vapor intrusion [VI] should meet the following [six] requirements” [21] (p. 17). A *second* reason is that following government technical requirements is relatively uncontroversial, as compared to following its mere recommendations. A *third* reason is that government waste-cleanup contracts typically award developers toxic-site-liability protection, but only if developers follow government testing/cleanup technical guidance, e.g., [22].

For critical environmental-health reasons, our question deserves analysis. A full 23% of US citizens, 54% of minorities, and 25% of children under age 5, live within 1 mile of brownfields or US CERCLA sites [23,24]. Yet as mentioned, given the absence of independent scientific assessment of potential testing violations and resulting health/EJ threats at representative hazardous-waste sites, most of which undergo privatized testing/cleanup, “little is known about the public costs and benefits” of privatized cleanups [25,26]. As a result, from California [27], to Colorado [27], to Indiana [28], to New York [27], to North Carolina [29,30], communities report VI, even at “remediated sites” [31,32]. 

Health and EJ threats from VI continue partly because the financial incentives in the 2002 US Brownfields Revitalization Act may have created a conflict of interest [18]: Private, for-profit parties conduct most US hazardous-site testing/remediation, which should serve the public interest/public health. Yet when privatized cleanups increase private profits, they typically decrease public-health protection; when they increase public protection, they typically decrease private profits [18,33]; see Supplementary Materials S2, Section S2.2. Hence the conflict.

## 2. Materials and Methods

What ***method*** can we use to evaluate our question, “Does representative US VOC-waste testing meet regulatory-testing requirements?” Because answering this question is not amenable to randomized, controlled trials—and because we must weigh multiple pieces of pre-existing evidence through meta-analysis, not merely generate evidence—we use the hazardous-waste assessment method developed/recommended by the US Environmental Protection Agency (EPA). This is the systematic, 2016, three-part, weight-of-evidence (WoE) method. Employed by most US hazardous-site assessors, as well as risk and environmental-impact assessors, WoE is EPA’s main method for weighing multiple pieces of often-conflicting lab, field, statistical, or modeling evidence to infer policy-relevant or potential regulatory actions [34], especially at US hazardous-waste sites.

As such, the WoE method has three main parts: (1) assembling a body of positive and negative evidence regarding some question, e.g., “which of two hypotheses regarding adequate hazardous-site testing appears more likely, that waste testing follows or that it violates regulatory guidance?”; (2) formulating and justifying evidence-scoring procedures for the preceding question; and (3) evaluating the preceding scoring results to answer the question [34] (p. 1). In short, these three parts of the WoE method are (1) assembling relevant evidence, (2) scoring the evidence, and (3) evaluating the evidence scoring. The ***materials*** required for this analysis will be generated by WoE Method Part (1), then evaluated by employing WoE Method Parts (2)–(3).

### 2.1. WoE Method, Part 1: Assembling Positive and Negative Evidence

To assemble all relevant evidence/materials regarding the question whether US VOC hazardous-site testing follows regulatory guidance (WoE Part 1), we develop and justify systematic selection criteria for two different types of evidence. These are (1.1) government regulatory-guidance documents for testing subsurface VOCs and (1.2) actual site-testing documents from a representative sample of hazardous-waste sites. Our aim is to assess whether actual testing complies with government requirements for testing. 

Because government VOC-testing guidance is complex, and because US hazardous sites number up to one million [14], we provide only a partial, but arguably a representative and conservative sample (see Section 2.1, Section 2.1.1 and Section 2.1.2), to assess whether hazardous sites follow technical guidance for testing. That is, we employ only guidance and testing documents (1.1)–(1.2) that cover (a) privatized testing, (b) VOC hazardous waste, and (c) California sites. 

We use criterion (a) to help ensure sample representativeness because, as already noted, most US hazardous-waste testing/cleanup is privatized. We use criterion (b), as mentioned, because VOCs/vapor intrusion are emerging contaminants; because hundreds of thousands of US waste sites, even supposedly remediated sites, still face VOC contamination; and because the university consultation request addressed a prominent VOC site. Finally, we use criterion (c) to help ensure sample representativeness and conservativeness because, among US states, California has:

- the most US military-hazardous-waste sites, most with VOCs [35];
- an environmental-leadership reputation [36] that should provide a conservative response to whether VOC-waste testing follows regulatory guidance;
- the second-highest number of US CERCLA sites [37]; and
- Envirostor, the preeminent hazardous-waste-site database [38,39].

#### 2.1.1. WOE Method, Part 1.1: Criteria for Evidence, Federal/California VOC-Test Guidance

To implement WOE Part 1.1 selection ccriteria, for federal/California regulatory-guidance documents, we use two explicit, comprehensive, and reproducible search strategies, one for California state-government, and one for federal, documents. The two-part federal search strategy is given in Supplementary Materials S2, Section S5.1, not here in the text, to provide manuscript readability, unencumbered by technical details that might obscure the thread of the argument. 

The three-part California guidance search strategy is more complex than its federal equivalent because all federal VOC-waste guidance is in US EPA databases. However, analogous California guidance is in multiple databases, depending on which state agency generated it. Although this California search strategy is also explicit, comprehensive, and reproducible, it likewise appears in Supplementary Materials S2, Section S5.2, so that its many technical details do not impede manuscript readability.

#### 2.1.2. WOE Method, Part 1.2: Criteria for Evidence, California VOC-Site Documents

To help ensure the representativeness and conservativeness of our VOC-testing results, we assess whether waste testing follows guidance, but only at hazardous sites that meet seven precise selection criteria (from WOE Part 1.2), three already defended (see preceding i, vi, vii). The seven criteria specify that to be representative and conservative, the waste sites (whose VOC-waste-site testing we assess) must:

i. be at California locations, subject to state guidance, for reasons already given in Section 2.1.
ii. have sampling performed by/for TC, the largest US commercial developer [4], who “pioneered…privatized remediation” [40] and is “the industry leader in Brownfields development” (see Supplementary Materials S2, Section S2.6). As a result, TC likely has the economic resources, expertise, leadership, and size to conduct the best testing possible, thus to provide conservative test results that tend to follow guidance.
iii. have undergone testing/remediation since 2011, because California’s main subsurface-VOC/VI guidance appeared in 2011 [21], and our testing results should be current.
iv. have publicly accessible documents, on California’s Envirostor database, because no other US states provide public/internet access to virtually all hazardous-waste-site documents, which is a necessary condition for this analysis.
v. have subsurface-trichloroethylene (TCE) contamination, because TCE is a no-safe-dose, genotoxic carcinogen [41]; is subject to EPA’s urgent and accelerated-action requirements to protect health [42]; may produce fetal heart defects after only a brief, airborne, 0.5 µg/m^3^ TCE exposure during pregnancy [41,42]; and contaminates at least 926 California hazardous-waste sites [43].
vi. have multiple subsurface-VOC carcinogens (see (a)–(c) in Section 2.1), so as to ensure that this analysis evaluates some of the deadliest, most complex sites that pose the greatest environmental health/justice threats.
vii. be undergoing privatized testing/cleanup (see (a)–(c) in Section 2.1), because most US hazardous-waste-site testing/cleanup is privatized, as explained earlier.

To implement the preceding seven (WoE Part 1.2) selection criteria (i–vii), we employed four specific, transparent, reproducible search strategies for California subsurface-VOC-testing-site documents. They are complex for at least two reasons. *First*, no single California database lists all VOC-hazardous-waste sites controlled by the federal government, state government, or US military. *Second*, none of the databases has all the filters that allow searches for key terms in our question/search criteria, such as VOC or TCE. Given the complexity of the three-page-long search strategy (WoE Method Part 1.2, California hazardous site), it appears in Supplementary Materials S2, Section S2.6, not here, so as to avoid encumbering the text with technical details that only some readers wish to see. 

### 2.2. WoE Method, Part 2: Formulating and Justifying Evidence-Scoring Procedures

WoE Part 2 employs two procedures to formulate and justify evidence-scoring procedures to evaluate the question this analysis investigates. The first procedure identifies the characteristic used to conduct environmental scoring of whether VOC testing follows regulatory guidance. This characteristic is any method or procedure that US EPA or the California Department of Toxic Substances Control (DTSC) VOC regulatory-guidance documents calls a soil-gas-VOC-testing “requirement” (search term “requir”) [21] (p. 17). Because state and federal governments identify this scoring characteristic as dictating mandatory or compulsory aspects of all VOC testing, it is not merely optional, recommended, or suggested technical guidance; instead it is what regulators uncontroversially consider essential for accurate, reliable testing. 

The second WoE Part 2 procedure explains how to score each violation of a VOC-testing requirement: score each violation, at each hazardous site, as 1. That is, for any hazardous site y_i_, violations are the sum of violations of each requirement x_i_: (x_1_ + x_2_ +….x_n−1_ + x_n_). Violation numbers range from 0 to x_n_; for all assessed hazardous sites y_n_, total violations are the sum of each site’s violations. Maximum violations number (x_n_) (y_n_).

### 2.3. WoE Method, Part 3: Evaluating the Part 2 Scoring of Evidence

WoE Part 3 employs four procedures, based on WoE Part 2, to assess whether representative hazardous-waste-site testing meets or violates government-regulatory requirements. The first procedure is to electronically search all VOC-testing documents (from all VOC-waste sites obtained in WoE Part 1), for terms in each government-formulated requirement (obtained in WoE Part 2), so as to systematically determine whether each testing document violates or meets each requirement. The procedure then uses WoE Part 2 to score each requirement violation. Finally, the procedure dictates adding all instances of requirement violations from all electronically-searched documents. 

The second procedure is to hand-scan each VOC-testing document (from each VOC-waste-testing site obtained in WoE Part 1), for sections that discuss any methods/terms relevant to VOC-testing requirements (obtained in WoE Part 2), so as to systematically determine whether each methodological section contains text showing that it violates or follows each Part 2 requirement. The procedure then uses WoE Part 2 to score each requirement violation. Finally, the procedure dictates adding all instances of violations from all hand-scanned, VOC-test-site documents. 

The third procedure is to add all violations from the previous e-searches and hand-scans, so as to obtain the total number of violations for all VOC sites assessed. Next one compares the numbers of actual, to total potential, violations. Total potential violations are represented by (x_n_) (y_n_), where x_i_ is each requirement, x_n_ is the total number of requirements, y_i_ is each testing site that meets WoE Part 1 criteria, and y_n_ is the total number of testing sites meeting these criteria. Actual violations are represented by (the number of VOC sites that violate requirement x_1_) + (the number of VOC sites that violate requirement x_2_) +….+ (the number of VOC sites that violate requirement x_n−1_) + (the number of VOC sites that violate requirement x_n_).

The fourth procedure is to determine, for each of the preceding scored violations, whether any situation-specific circumstances might justify saying that no violation occurred. (For instance, suppose there were a guidance requirement to test all VOC-soil-gas levels, on a 10-foot-by-10-foot horizontal grid, at 5-foot-depth intervals, from the surface to groundwater at 50 feet. However, if using this procedure showed that all surface-deposited, soil-gas VOCs had been attenuated to the level of 0 µg/m^3^ within 30 feet subsurface, and if there were no preferential VOC-migration pathways, then one could justify not following the requirement to test soil between 30 and 50 feet subsurface). If anyone explained/defended such situation-specific circumstances, and thus provided uncontroversial scientific justification for not following some aspect of guidance-required testing, then one could delete the supposed violation (from the sum of total violations already obtained); see A5 below. If there were no such circumstances, then the total number of VOC-waste-site violations would be the same as given in the preceding third procedure. 

Thus, to answer our question (“Does a representative sample of subsurface-VOC-waste sites tend to meet or to violate regulatory-testing requirements?”), one would merely add all violations (less any violations that had defensible, situation-specific justifications), then compare total violations to potential violations. Fewer violations would make a “meet” response (to the question) more likely. Greater numbers of violations would make a “violate” response more likely; see Supplementary Materials S2, Sections S2.4–S2.7.

### 2.4. Nine Methodological Assumptions

At least three assumptions (about using the WoE Method, already defended in Section 2.1.2) are central to the seven criteria employed for choosing a representative sample of VOC-waste-testing sites in WoE Method, Part 1.2. Assumption A1 is that California VOC-waste testing by TC, the industry leader in hazardous-site testing, is likely to reveal conservative testing violations. A corollary assumption, A2, is that if industry-pacesetting redeveloper TC exhibits excellent VOC-site testing in California, then such testing is possible elsewhere in the US. Another corollary assumption, A3, is that if TC exhibits guidance-violating, VOC-site testing in wealthy, populous, environmental-leader California, with its superior VOC-testing requirements, then such testing violations may be more likely elsewhere in the US and the world.

In addition to the preceding three assumptions, there are at least six main additional methodological assumptions, A4–A9. A4 is that, for reasons already stated (Supplementary Materials S2, Sections S2.5–S2.7), there are **no specific/quantitative/methodological rules** for following soil-gas-VOC-testing requirements. Instead, each scientific-rule-following situation is different, requiring rule interpretation. Thus one cannot show empirically (but only argue) that some scientific-rule-following is correct, unless one refers to other scientists’ expectations which, by definition, are not empirical [44].

A5 is the assumption that **government guidance specifies default-testing “requirements”** [21] (p. 17) that scientists must follow, unless they provide “adequate technical documentation” that their “alternative” tests are “technically equivalent” to requirements [45] (p. 2), [21] (pp. 1–2); see Supplementary Materials S2, Section S2.7 (no assessors provided such documentation).

A6 is the assumption that our **results provide only potentially indefensible testing violations**, as TC provides no evidence that its alternative/shortcut tests are either defensible (technically equivalent to required tests [45] (p. 2), [21] (pp. 1–2)) or accurately represented by TC. Yet both types of evidence are necessary conditions for showing the all-things-considered indefensibility and technical equivalence of TC’s testing (see L3).

A7 is the assumption that **WoE can provide no randomized/controlled hypothesis testing**, only systematic/transparent meta-analysis [34], as WoE is a method for assessing empirical testing, based on regulatory requirements for testing; WoE is not itself empirical testing.

A8 is the assumption that the preceding three-part WoE method can be used to assess guidance-following at VOC-waste testing in any US state that uses the same VOC guidance documents (namely, those of the US and California) and the same WoE criteria for choosing representative VOC-waste-testing sites that we use.

More generally, A9 is the assumption that **the preceding three-part WoE method can be generalized**, and thus used to assess the guidance-following of waste testing in any nation/state, provided at least three conditions are met. *First,* one must justify and correctly employ specific, public, representative, reproducible criteria for selecting which hazardous-waste regulatory-guidance documents (of some state/nation) to use. *Second*, one must justify and correctly employ specific, public, representative, reproducible criteria for selecting a sample of hazardous-waste sites whose testing to evaluate. *Third*, one must have full access to all relevant waste-testing documents. However, for reasons already mentioned, this condition is rarely satisfied, except perhaps in California. 

## 3. Results

### 3.1. WoE Method, Part 1 Results, Evidence: Guidance and Testing-Site Documents

The WoE Method, Part 1.1 results are government VOC-testing technical-guidance documents meeting Part 1.1 criteria from Section 2.1.1. These are three US EPA- [42,46,47] and five DTSC-guidance documents [21,45,48,49,50]. We include the 2020 DTSC-draft-guidance document [45] because DTSC now requires its use, and in 2021 archived the 2011 document.

The WoE Method, Part 1.2 results are privatized-testing documents from all waste-site facilities meeting the seven selection criteria, i-vii (Section 2.1.2). Results include all testing documents from the following sites.

- the 9-acre former US Naval Ordnance Testing Station, Pasadena, California (NOTSPA), Envirostor ID 19970020 [51];
- the 10-acre former heavy-manufacturing site, Monrovia, California, Envirostor ID 60002828 [52];
- the 51-acre former Raytheon missile site, Canoga Park, California, Envirostor IDs 41162124,80001366; Geotracker ID WDR100000974 [53];
- the 33-acre former Branford Landfill, Pacoima, California, Envirostor ID 19990021, Geotracker ID L10002785228 [54];
- the 18-acre former Santa Fe Railyards, Boyle Heights (Los Angeles), California, Envirostor ID 19400008 [55].

### 3.2. WoE Method, Part 2 Results: Scoring Regulatory Violations

The WoE Part 2 results are six government test “requirements” for assessing subsurface VOC contamination. That is, DTSC explicitly says that, to guide cleanup and protect health, “soil-gas [-VOC-testing] data for evaluating vapor intrusion should meet the following [six] requirements” or compulsory actions. These are that all sampling must

- (R1) be “collected near contaminant **sources**” [21,46], that is, direct-exposure origin points, reservoirs “that sustain a [VOC] contaminant plume” in soil/soil-gas/groundwater/air [56] (p. 34).
- (R2) be “from permanent/**semi-permanent wells**,” thus allowing multi season/same-location samples’ capturing seasonal/temporal contaminant variations, necessary for R3 [21,46].
- (R3) “represent **steady-state conditions**,” non-migrating contaminants [21,46].
- (R4) “follow Cal/EPA’s Active Soil-Gas[-VOC] **Investigation Advisory”** [21,46],
  including soil-gas testing that:
  (R4.1) continues “until vapor-phase **contaminants are no longer** **encountered**,” thus providing “subsurface,” **“three-dimensional” toxin delineations;**
  (R4.2) provides a “minimum, **two-sub-slab sampling events**” at all building centers;
  (R4.3) locates all “**maximum subsurface concentrations**” [48].
- (R5) employ method-detection/**reporting limits “lower than health-protective [screening]** levels” [21,46].
- (R6) be dense enough “to accurately extrapolate [all-depth, all-location, toxin-con-centration maps or] isoconcentration contours” [21,46].

### 3.3. WoE Method, Part-3 Results: Evaluating the Scoring of Regulatory Violations

The WoE Method Part 3 uses its Part-2 results (six government VOC-testing requirements, R1-R6 above) to assess testing at five VOC-waste sites. Recall that the five VOC-waste testing sites, discovered through WoE Part 1, are located in Pasadena (NOTSPA), Monrovia, Canoga Park, Pacoima, and Boyle Heights, California. Thus, total possible testing violations number 30 for all five hazardous-waste sites.

#### 3.3.1. Violations of Testing Requirements: Pasadena and Monrovia

Subsequent paragraphs show that, surprisingly, both Pasadena (NOTSPA) and Monrovia hazardous-waste-site testing violate all six of the preceding six regulatory requirements for VOC testing (R1–R6). The main contaminants at both sites are VOCs [10] (p. 34); [52] (Phase II Report).

Though NOTSPA “risk drivers” are chlorinated-VOC solvents and carcinogens CT, DBCM, PCE, and TCE [10] (p. 34), the Pasadena-site developer’s only remediation will be removing 11–13 metals-hotspots/drains [10] (pp. 20–42) (see Figure S1, Supplementary Materials S1), but leaving most VOCs onsite [10] (p. 34). Other contaminants include arsenic, dioxins, furans, hexavalent chromium, lead, mercury, nitroisodimethylamine, polychlorinated biphenyls (PCBs), polyaromatic hydrocarbons (PAHs), perfluorinated substances (PFAS), petroleum hydrocarbons, radioactive materials, explosives and propellants such as royal demolition explosive (RDX) and TNT, and other VOCs/semi-VOCs [57]. 

As Table 1 and Table 2 illustrate, most NOTSPA VOCs will remain “in place” [58], at levels up to 424,000 times above the 10^−6^ risk level, the health-protective, residential-screening level required for the planned 550 NOTSPA apartments; yet 40% of these apartments are for families with children [10,58]; see last column, Table 1 and Table 2. Table 1 and Table 2 below provide PCE-CT soil-gas levels because they likely pose NOTSPA’s highest human-health risks [10] (Appendix D, Table 3); see [59]. Table 3 shows how and why both the Pasadena NOTSPA and Monrovia hazardous-waste sites violate all six government-regulatory requirements for VOC testing.

- Columns 3 and 5 of Table 1 and Table 2 illustrate R1-requirement violations: most of the hundreds of PCE/CT sources (including those shown) will not be removed, as they are not in the 11–13 metals-hotspots/drains, which are the only areas that the redeveloper will remove onsite.
- Column 7 of Table 1 and Table 2 illustrates R2-R3 violations: tests provide one-time/temporary-well samples, not evidence for steady-state conditions, yet steady-state conditions are necessary for sampling; otherwise higher levels of contaminants are possible, and these higher levels are more likely both to migrate and to harm people.

At the Monrovia hazardous site, contaminants include PCE, TCE, trichlorofluoromethane (TCFM), other VOCs, aniline, anthracen, antimony, arsenic, barium, beryllium, cadmium, chromium, cobalt, copper, (E)-4-(2-Methoxystyryl) phenol, lead, mercury, molybdenum, nickel, PCBs, petroleum hydrocarbons, pyrene, selenium, tert-butyl alcohol, thallium, vanadium, and zinc [61]. Table 3 illustrates that the Pasadena and Monrovia sites violate all six requirements for VOC testing (R1–R6). Table 4 illustrates Pasadena and Monrovia violations of R5, namely, using methods that are too insensitive to detect many contaminants.

In summary, all Pasadena and Monrovia hazardous-site, soil-gas-test-VOC violations cause VOC-contaminant underestimates, given testing that invalidly limits sampling numbers (thus violating testing requirements R2, R4.3), as well as sampling range and distribution (thus violating testing requirements R1, R4.1–R4.3, and R6).

#### 3.3.2. Violations of VOC-Testing Requirements: Canoga Park and Pacoima Sites

At Raytheon’s former Canoga Park nuclear-/missile-testing facility [64], TC is developing the 36-acre Southern Parcel. Contaminants include 1,1,1-trichloroethane, 1,1-dichloroethane, 1,1-dichloroethylene, 1,2-dichloroethane, arsenic, benzene, beryllium/compounds, cadmium/compounds, hexavalent chromium, petroleum hydrocarbons, radioactive isotopes, PCE, chromium, TCE, TCFM, uranium, and vinyl chloride [65,66,67].

The Pacoima site is the former Branford Landfill. Its contaminants include VOCs 1,1,1-trichloroethane, acetone, bis(2-ethylhexyl)phthalate, CT, chloroethane, chloroform, di-nbutylphthalate, ethylene dichloride, methyl ethyl ketone, PCE, TCE, TCFM, vinyl chloride, carbonyl sulfide, carbon disulfide, lead, and manganese [68,69]. Unlike other sites, Pacoima meets test requirement R2, as it has seven semi-permanent soil-gas VOC probes, allowing collection of multiple samples, over time, at a location; however, probes exist only at the north/northwest borders [70]. Thus no data reveal VOC levels at the eastern, southern, or central parts of the site. However, regulatory data show the site has experienced multiple pollutant-level violations and mandatory safety-enforcement actions [71].

As Table 5 shows, Canoga Park and Pacoima sites violate most VOC regulatory-testing requirements. Many Canoga Park violations result from using only three temporary-well one-time sampling locations [72]; see Supplementary Materials S1, Figure S15. All the violations cause contaminant underestimations because such testing invalidly limits the sampling range and distribution (violations of requirements R1, R4.1, R4.3, R6), sample numbers (violations of R2, R4.3), and equipment/method sensitivity, needed to capture all above-health-protective-level concentrations (violations of R3, R5).

#### 3.3.3. Violations of Testing Requirements: Boyle Heights

TC’s Boyle Heights (Los Angeles) hazardous site, the former Santa Fe Railyards and Crown Coach factory, has VOC contaminants 1,1,1-trichloroethane, 1,1-dichloroethane, benzene, chloroform, cis-1,2-dichloroethene, dichlorodifluoromethane, dichloroethylene, PCE, and TCE, along with trans-1,2 dichloroethene, heavy metals, and petroleum hydrocarbons [79,80]. Though it was certified as remediated in 2019 [81], Table 6 shows its sampling violates all six test requirements, causing systematic contaminant underestimations, as tests invalidly limit the sampling range/distribution (violating requirements R1, R4.1–R4.3, R6), numbers (violating R2, R4.3), and equipment/method sensitivity (violating R3, R5).

In conclusion, as Table 7 summarizes, all five sites reveal the same pattern of violating most of the six regulatory requirements for testing VOC contaminants.

To summarize: our results show that four of five representative US privatized hazardous-site VOC cleanups violate six of six government-regulatory requirements for VOC testing, and one cleanup violates five of six such regulatory requirements; total violations number 29 of 30 that are possible. All violations arise from not conducting full-site tests, not capturing maximum-level concentrations, limiting sampling ranges/distribution (violations of requirements R1, R4.1–R4.3, R6) and numbers (violations of R2, R4.3), and using equipment/methods/procedures that are insensitive to many levels of VOC contamination (violations of R3, R5).

## 4. Discussion

To evaluate the significance of the preceding results, we discuss 5 questions. 

### 4.1. Question 1: Do the Preceding Violations of Regulatory Requirements for Hazardous-Waste Testing Suggest Public-Health Risks from VOCs?

#### 4.1.1. Health Threats from VOC-Waste Undertesting/Underreporting

As already mentioned, TC’s violations of regulatory requirements for VOC testing may threaten health because, at all five privatized site cleanups, assessors use VOC undertesting/underreporting—which results in reduced, less-costly, cleanups.

At Canoga Park, assessors provided neither sitewide sampling [73] nor all-contaminant, all-depth isoconcentration maps [73], both of which regulations and safety require. Instead, they sampled only three locations, each only 20% of the way down to groundwater, though regulatory guidance requires full testing to groundwater, mainly because VOCs migrate downward [72]; Supplementary Materials S1, Figure S15. When all Canoga Park test locations showed TCFM increasing with depth, assessors stopped sampling at, e.g., 33 feet, with its 260,000 µg/m^3^ TCFM [72] (Figure 4), which is 200 times above the approved SL/health-protective level [10] (Appendix D, Table 3). Ignoring the increasing TCFM levels; that VOCs can migrate indoors from 100+ feet [45], not just 33 feet; and that guidance requires further testing, assessors instead claimed that “concentrations do not pose a significant impact” [72]. They also proclaimed, without evidence or explanation, that “no further [clean-up] action” [72] (p. 1) was needed.

At Monrovia, assessors likewise conducted no sitewide testing, although regulatory guidance requires it. Though subsurface VOCs travel to deeper soil/groundwater/indoor air [21], assessors took only 21 samples (all at only 5 feet subsurface), on only 4 of 10 acres, and ignored deeper soil [62] (Table 1), contrary to requirements. They also ignored the fact that VOCs migrate indoors from 100+ feet subsurface [45], not just 5 feet. Nonetheless, they claimed, without evidence or explanation, that “No data gaps” exist, and that “hazardous materials….do not appear to have impacted subsurface conditions…no further assessment [therefore clean-up] is warranted” [83] (pp. 17–18).

At NOTSPA, as already mentioned, all 172 known subsurface soil-gas VOC locations have contaminant-violations that range from 300 to nearly 1,000,000 times above health-protective/residential-screening levels [10] (Appendix D, Table 3; Appendix E). Yet TC’s site documents allege **only “low-level VOC impacts**… [and thus contaminant] mitigation [not removal] will be performed, as necessary” (author’s bold) [10] (p. 38). However, regulatory guidance requires removing, not just mitigating, any VOCs that are more than 100 times above SLs/health levels [21] (p. 36), [49,50], and NOTSPA VOCs are 300–750,000 times above SLs. 

As the three preceding paragraphs show, using VOC undertesting/underreporting, potentially to justify inexpensive contaminant mitigation (instead of more expensive remediation/removal) suggests determinate bias. Why? A total of 100% of the 29 of 30 VOC-testing violations have unidirectional effects, namely, contaminant/risk underestimation. Yet because random or accidental violations should have bidirectional effects, this testing (sites assessed here) appears biased. Such VOC undertesting/underreporting also is consistent with TC’s risk under-calculation, which violates DTSC calculation requirements (see Supplementary Materials S2, Section S2.8) and is consistent with earlier analyses showing that TC testing has failed data-quality analysis [84], scientific-data audits [85], and data-usability analysis [86].

#### 4.1.2. Health Threats from Poor Regulatory Oversight of Privatized Testing

A second reason these results (showing 29 of 30 possible VOC regulatory-testing violations) threaten health is their arising from flawed DTSC oversight and enforcement. For instance, apart from not enforcing its own VOC-testing requirements, DTSC required no NOTSPA indoor-air tests. However, any of at least five facts should have triggered NOTSPA indoor tests, if the regulator were following its VOC-testing requirements:

- **DTSC’s own guidance requires** indoor-air tests when soil-gas risks exceed 10^−6^ [45] (pp. 17, 25); [21] (p. iii). Although all 172 NOTSPA soil-gas-sample risks far exceed 10^−4^ and many exceed 10^−2^, regulators forced TC neither to assess nor to remove VOC indoor-air threats to site renters [10] (Appendix D, Table 3); see Supplementary Materials S1, Table S1.
- DTSC’s 2004 ***Imminent and Substantial Endangerment Determination and Remedial Action Order*** warned about current site-renter risks from carcinogenic VI and ordered full testing and cleanup—neither of which has ever occurred [87].
- In exchange for site-liability protection, **DTSC-TC contracts require** TC to follow all technical guidance (including required indoor-air and soil-gas testing), so as to be able to conduct a CERCLA cleanup [88,89] (Exhibit E). Yet TC has not followed guidance.
- Despite onsite renters, **25 of 29 buildings had no required subslab soil-gas VI tests** [21] (p. 22), [45] (p. 21), and no buildings had indoor-air VI tests—both of which are required by VOC regulatory-testing documents.
- Responding to requests from the community near NOTSPA and tenant risks, in 2021 University of Notre Dame scientists conducted indoor-air tests of NOTSPA units whose tenants requested it [90]. **All 11 tested site locations violate all three California safety benchmarks** and have two-week-average indoor-air carcinogen concentrations > 10^−6^, namely, concentrations up to 4.4 (10^−4^)–2.0 (10^−5^), which violates **California’s No Significant Risk Levels** (NSRLs) [90] (Table 7); up to 1.9 (10^−4^)–8.7 (10^−5^), which violates **California’s Inhalation Cancer Risk** [90] (Table 8); and up to 6.7 (10^−5^)–3.1 (10^−6^), which violates **Environmental Screening Levels** [90] (Table 6). Yet in response to presentation of these published certified-lab results, regulators did nothing to protect site tenants.

Instead of following the preceding five regulatory requirements for indoor-air testing, and without any indoor-air-VOC evidence of its own, DTSC instead claimed NOTSPA “is safe at this time” [91]. Though all five privatized VOC sites (assessed here) have current renters who could be harmed, DTSC has required no indoor-air tests at any sites. Such apparently lax oversight may explain why, after more than 10 years of failed legislative efforts to reform DTSC, in July 2021 the California Legislature passed the DTSC Reform Bill (SB 158). It explained that:

“Over the past decade…DTSC has received complaints [that]…DTSC is not properly enforcing state and federal law…. Numerous statutory changes have been made to…address outstanding programmatic failings. However, many of the underlying concerns about [DTSC] transparency… [and] accountability…remain”

[92] (pp. 6–7).

Another example of DTSC-oversight failures is Exide Battery in East Los Angeles (LA), one of poorest, most-polluted Latino communities, and home to disproportionate numbers of children. For 30 years and without requiring any testing, DTSC claimed Exide’s impacts on East LA were “less than significant.” Only in 2020 did nearby victims force DTSC to issue an Imminent and Substantial Endangerment Order, to recognize Exide’s four felonies, its illegal releases of 3500 tons of lead, and its causing permanent IQ deficits and chronic health harm among 250,000 nearby residents, mostly children. Because of failed DTSC oversight, thousands of East LA children now have unsafe blood-lead levels (double the LA average lead level) that will limit their opportunities in life [93,94].

#### 4.1.3. Health Threats from TC Misrepresentations of Results of Its Privatized Testing

TC-testing violations also threaten health because, at least at the sites examined here, TC appears to misrepresent its flawed, privatized testing as reliable [84,85]. For example, Canoga-Park assessors ignored DTSC-approved TCFM SLs or health-protective levels [10] (Appendix D, Table 3); see Supplementary Materials S1, (Table S1). Instead, TC assessors falsely wrote in site documents that because no SLs were “readily available,” they self-calculated a 730,000 µg/m^3^ TCFM SL [72] (p. 14)—a level that is 562 times higher than the DTSC-approved SL [10] (Appendix D, Table 3). The TC assessors then claimed that Canoga Park soil-gas TCFM levels “were below all available…SLs” [72] (p. 1), yet 92% of all site samples violate the DTSC-approved SLs/health-protective levels by at least two orders of magnitude [10,72] (Appendix D, Table 3).

At NOTSPA, TC likewise claimed it “extensively investigated and tested” and “appropriately tested…the entire site,” although it violated all six of six DTSC soil-gas VOC testing requirements (see Table 7). TC also repeatedly made official guarantees to the Pasadena City Council that it would remediate NOTSPA “to the highest applicable standards” [91]. As Table 8 illustrates, such “highest standards” claims are false because:

- TC’s site technical documents show 15-foot, TCE, PCE, and CT soil-gas cleanup levels that are 12,400 µg/m^3^, 5470 µg/m^3^, and 705 µg/m^3^ [58]—which are, respectively, ***25,833 times, 11,891 times, and 10,522 times less protective*** than the highest-applicable residential standards (which are 0.48 µg/m^3^, 0.46 µg/m^3^, and 0.067 µg/m^3^) [21,45]. Residential standards are the 10^−6^-cleanup level that TC promised [91] and that both DTSC and US EPA regulations require for residential sites like NOTSPA.
- TC’s own site documents guarantee only 15–20-foot-subsurface partial shallow-soil cleanup [58] (p. 45). Therefore they ignore DTSC and US EPA Technical Guidance which warns that VOCs at least 100+ feet subsurface can cause carcinogenic VI [21,45,46]. Violating DTSC/US EPA technical guidance, TC instead claims it will use VOC mitigation—carcinogen monitoring, open-air carcinogen venting, slurry caps on a few VOC spots instead of cleanup, and land-use restrictions [58] (pp. 45–54). However, the “highest applicable standards” exclude such land-use restrictions. They require **full residential cleanup (10^−6^),** not just risky mitigation and land-use restrictions.
- TC’s own documents also admit that it will not remediate VOCs that are 20–150 feet subsurface that are 300,000–400,000 times above DTSC’s 10^−6^ cleanup level (see Table 1 and Table 2). However, regulatory documents show these contaminants could cause VI, indoor-air cancers, and birth defects among site residents. TC documents also admit that remaining NOTSPA contaminants “may exceed” regulatory requirements [58] (p. 48). Again, all such admissions by TC contradict its highest-standards-cleanup claim [91].
- TC documents likewise say NOTSPA VOCs are “low level” [10] (p. 38), yet its own test data show all 172 tested locations have VOC levels 300–750,000 times above required residential cleanup health-protective levels [10] (Appendix D, Table 3; Appendix E).
- TC claims NOTSPA “soil outside the storm-drain system is expected to be non-hazardous” [58] (p. 42), contradicting its own test data (see Table 1 and Table 2).
- TC claims NOTSPA northern soil is “clean” [58] (p. 60), [95], contradicting its test data (see Table 1 and Table 2, last column).
- TC officially claimed and wrote to Pasadena City Council that “all of the [toxin-] impacted soils” will “be removed” from the NOTSPA site [91], yet its own site documents clearly show the opposite (see Table 8 below):

In summary, TC’s misrepresentations of its hazardous-site testing and DTSC’s regulatory-oversight deficiencies may exacerbate TC’s apparent violations of VOC-testing requirements. As a result, they may pose additional threats to environmental health.

### 4.2. Question 2: Will EJ Communities Bear Most Burdens from VOC-Testing Violations?

Because EJ communities host all five privatized VOC cleanup sites assessed here, testing violations could worsen their health, and thus the economic burdens borne by these already-at-risk communities. For instance, **the California regulator’s CalEnviroScreen, the online multiple-pollutant indicator, ranks Pacoima and Boyle Heights toxic site census** **tracts among California’s 4% most polluted** [96,97]. Boyle Heights’ population is 94% Latino and 46% foreign-born, with 42% more children than California’s average [98]. Pacoima’s population is 85% Latino and 47%foreign-born, with 30% more children than California’s average [99]. Testing violations could worsen both communities’ above-state-average levels of air pollution, hazardous sites, poverty, underemployment, and threats to children [96,100].

**Canoga Park’s** former Raytheon missile site is part of an 81% minority, 63% Latino, EJ community dominated by Cold-War government-weapons-testing contractors [101]. VOC-testing violations, at the hazardous-waste sites that these contractors created, add to the health problems facing the Canoga Park community. For decades local health has also been threatened by Boeing’s 2700-acre Santa Susana Field Lab (SSFL), two miles northwest of the Canoga Park hazardous site. SSFL is an unremediated US CERCLA site that conducted missile and other weapons testing, along with nuclear-reactor testing and plutonium and uranium fuel fabrication for nuclear weapons and reactors. Of 10 onsite nuclear reactors, none had any containment, and four had serious accidents, including two meltdowns. One of these core melts caused hundreds to thousands of Los-Angeles-area cancers and radiation releases 132 times higher than Three Mile Island. Given US-government weapons-complex secrecy, no-one ever conducted full epidemiological studies of the site. Nevertheless, in investigations of Canoga Park hazardous-site exposures, UCLA and US Geological Survey contaminant-monitoring assessments show that SSFL has caused chronic (1948–present) Canoga Park radionuclide, VOC, and metals exposures [102,103]—none of which has ever been fully evaluated or remediated.

At the **Pasadena and Monrovia** sites, subsurface VOC-testing violations also could worsen risks for five EJ populations. One such population is the disproportionately minority, uneducated, unemployed, low-birthweight, asthmatic residents of the census tract in which NOTSPA is located; this census tract is one of California’s 7% most polluted (see Introduction). The second near-NOTSPA EJ population is patients at Kaiser’s hospital-size/medical/urgent care facility, abutting the east side NOTSPA. The third NOTSPA EJ population is the middle-/high-schoolers and college students taking classes at Pasadena City College, abutting half of the north side of NOTSPA [86].

A fourth EJ population is future site residents, especially disproportionate numbers of children, in TC’s planned 986-unit Pasadena NOTSPA and Monrovia hazardous-site apartment redevelopment, abutting the 10-lane Interstate-210, a major east–west Los Angeles diesel-truck artery [104]. Though 31% of California households include children who are age 18 or younger [105], 40% of these Pasadena-/Monrovia-hazardous-site apartments are for families with children; they will face both subsurface VOCs and airborne-freeway-cancer risks that are 1500 times higher than California’s average cancer risks [106]. Although California recommends against building homes, medical facilities, daycare centers, schools, or playgrounds within 500 feet of freeways [107], TC ignored these recommendations and is building family apartments in both waste-site locations.

A fifth EJ population is blue-collar workers, renting units on the former NOTSPA. The already-cited (Section 4.1.2.) 2021 University of Notre Dame tests show that the renters’ current indoor-air risks are hundreds of times above all three allowed safety benchmarks in California [90]. In short: **EJ communities host all five privatized cleanup sites where systematic VOC-testing violations will likely worsen both their health and the pollution they face.**

### 4.3. Question 3: Does Previous Research Support Our Results?

As already mentioned, no refereed publications—except in 2021 by the author [84]—systematically assess whether hazardous-waste testing, most of which is privatized, violates testing requirements such as scientific standards. However, some literature generally supports the results of this analysis. States like Connecticut, Massachusetts, and New Jersey have privatized most toxic-site testing/cleanup/mitigation functions, yet provide “virtually no” state-government oversight; however, every year a tiny percentage of such sites in several states receives annual safety audits, virtually none of which involve onsite visits or testing. Yet even these cursory audits show that 71% of hazardous-site testing/cleanups fail to meet minimal safety standards [18] (p. 183), [33]. Legal analyses likewise show that privatized hazardous-waste testing/cleanup can cause serious health threats [18,33]. Such data are consistent with the surprising results reported here. No independent, refereed, published data are contrary to the initial results reported here.

### 4.4. Question 4: What Are the Key Limitations of this Study?

This study has at least three limitations.

(L1) It provides only a *preliminary* assessment of only *five sites*, only *privatized waste* cleanups, only sites whose main contaminants are *VOCs*, and only *California sites* that meet the seven site-selection criteria outlined in the WoE Method [21,45]. Furthermore, it uses only regulatory technical requirements for assessing testing reliability.
(L2) It provides no quantitative analysis of potential harm from any poor testing.
(L3) Given the lack of access to private TC/DTSC/federal testing documents, this analysis identifies only at-face violations of requirements for VOC testing, not the all-things-considered defensibility of these violations. However, defensibility seems unlikely, given at least five red flags: (1) This analysis uncovered 29 of 30 possible VOC-testing violations; (2) All violations are unidirectional and uniformly underreport risks; (3) No site assessors explain/defend these testing-violations, as the regulator requires; none show reasons that their VOC testing was equivalent to required testing (see A1); (4) TC repeatedly misrepresents its privatized testing as reliable (see Question 1), instead of being transparent about its tests; (5) The results of this analysis, showing testing-requirement violations at representative waste sites, are consistent with other results, showing that testing at representative waste sites also fails data-quality analysis [84].

While it would be desirable to overcome the preceding three limitations of this analysis, this is not possible, at present, for at least four reasons.

***First,*** a more comprehensive analysis—covering hazardous-waste sites in other states/nations—is not possible until scientists have access to all hazardous-site-testing documents within a state/nation, as California’s Envirostor provides. Of course, the US EPA has data on federal or superfund sites [108], states like Massachusetts provide online hazardous-waste releases [109], and states like New Jersey have map-specific lists of brownfields [110], etc. However, in the US, only California’s Envirostor online database has associated testing documents, available for download, for all hazardous-waste sites for which California is responsible.

***Second,*** a more comprehensive analysis of whether hazardous-waste testing in different nations/US states follows technical regulatory guidance is not possible because despite US EPA VI technical guidance [46], VI guidance differs among US states and different nations; see, e.g., [111]. Internationally, field testing for VI is not common, except in countries like Australia, Canada, Denmark, the UK, and the US; in fact, most nations have no regulatory guidance for VI/VOC field testing [112].

***Third,*** given the preceding obstacles to the analysis of whether hazardous sites follow required VOC testing, it is unsurprising that US EPA admits that it does not know whether human health and environmental justice are protected near most hazardous-waste sites, whether supposed remedial actions actually alleviate waste-site deficiencies, or whether disadvantaged communities always face harmful, “disproportionate health effects” near hazardous-waste sites [113] (p. 6).

***Fourth***, even this limited analysis is important because it and its 2021 companion paper [84] are the first independent, systematic, and reproducible studies of the reliability of hazardous-waste testing. They are the first to begin (1) to address key data gaps regarding the quality of privatized hazardous-waste testing and its untested regulatory compliance [114]; and (2) to employ specific, explicit, comprehensive, and reproducible criteria for finding representative samples of hazardous-waste testing sites in a state/nation.

### 4.5. Question 5: What Are the Main Future Research and Policy Implications?

Our results—that a representative, conservative sample of **privatized, US, VOC-waste testing sites tend to violate government-testing requirements**—suggest the importance of examining non-privatized, non-California, non-VOC, or non-TC waste testing/cleanup, to see whether further results are consistent with those discovered here. Such investigations, however, will be impeded by the four problems noted in Section 4.4.

Future investigations likewise might compare different hazardous-waste exposures and resulting health impacts associated with sites that meet, versus those that fail to meet, government-regulatory-testing requirements. Finally, as already suggested in the previous section, researchers might examine the effects of test data availability/nonavailability on waste cleanup, given that few US states post online hazardous-facility-testing/cleanup documents [38,39]. As a result, other states and nations are likely to be less able than California to assess the adequacy of hazardous-waste testing.

Regarding future policy, an inexpensive way to improve the oversight of waste testing, environmental justice, and environmental health might be to require all testing/cleanups to have annual, routine, independent scientific-data audits (RISDA) [85,115]. Analogous to financial-data audits, RISDAs do not interfere with sampling, but merely check whether reported/published testing conclusions agree with test data. A RISDA likely would have shown TC problems, because after NOTSPA failed a RISDA [85], this red flag motivated the current study.

One legal reform to promote reliable testing might be for states to pass analogues of the US EPA’s scientific integrity laws. These are binding on federal contractors who are “conducting, supervising, communicating, and utilizing [scientific testing and] results” [116]. If California’s privatized toxic-site cleanup contracts had included such a state contractor integrity clause, this might have prevented the testing violations outlined here [84,85,86,90]. More generally, governments might reevaluate their commitments both to overseeing hazardous-site testing/cleanup and to ***privatizing most toxic waste*** cleanups. After all, waste-site assessors’ already-mentioned financial conflicts of interest may be one reason for the apparent California violations of VOC-waste testing requirements.

## 5. Conclusions

Our results (29 of 30) reveal a representative, conservative sample of privatized-testing violations that answer our study question in the affirmative: **Does US hazardous-waste testing (most of which is privatized), at representative US subsurface-VOC sites, tend to follow or to violate regulatory “requirements” for VOC testing?**

If our results can be replicated, they suggest that inadequate waste-site testing may put at risk both environmental health and EJ. Flawed waste testing/cleanup may force some members of EJ communities to choose between hazardous housing and no housing. Our analysis also suggests that privatized cleanups may face difficulties like those of private prisons. California’s governor recently abolished private prisons, claiming they are “driven to maximize…profits… [and] lack proper oversight” [117]. Does privatized waste testing/cleanup do the same?

## Figures and Tables

**Table 1:** Highest of 172 known-soil-gas-VOC-violation locationsa for per/tetrachloroethylene (PCE), single or temporary-well samples, NOTSPA, Pasadena, California.

Sample ID ^a^	North or South Part of Site?	Do TC Documents Identify Sample as from a PCE Source ^b^?	Is It a Sub- Slab Sample? ^b^	Does TC Consider Sample Area “Non-Hazardous,” Thus Not to Be Removed ^c^? (Not Hotpot/Drain)	Depth in Feet	PCE (µg/m^3^) ^a^	Times above 10^−6^ PCE Health-Protective or Screening Level (0.46 µg/m^3^) ^d^ (÷col 7 by 0.46)
NMSV10-5	N	Y	Y	N	5	342,000	743,480
V9-15	S	Y	N	Y	15	137,000	298,000
VD2-30	S	N	N	Y	30	122,000	265,217
V5-15	S	N	N	Y	15	79,000	172,000
V9-10	S	Y	N	Y	10	39,100	85,000
V10-5	S	N	N	Y	5	36,300	79,000
NMSD3-60	S	N	N	Y	60	22,300	48,480
V6-15	N	N	N	Y	15	20,500	45,000
VD1-20	N	N	N	Y	20	20,400	44,347
NMSD3- 113	S	N	N	Y	113	17,900	38,913

^a^ [10] (Appendix D, Table 3) and [10] (Appendix E) show all 172, all one-time/temporary-well samples; all violate PCE health-protective/screening levels; see Supplementary Materials S1 (Table S1 and Figure S2). ^b^ Assessors identify only V9-10/V9-15 and NMSV10 above as PCE (secondary) sources [6] (Figures 9 and 10); see Supplementary Materials S1 (Figures S2–S6). Secondary sources [21] (p. 5) are direct-exposure starting points, reservoirs sustaining contaminant plumes in groundwater/air/soil gas, [56] (p. 34). ^c^ [58] (p. 42). ^d^ DTSC [60], used in [6,10].

**Table 2:** Highest of 172 known soil-gas-VOC-violation locations ^a^ for carbon tetrachloride (CT), single or temporary-well samples, NOTSPA, Pasadena, California.

Sample ID ^a^	Is Sample from North or South Part of Site ^b^?	Do TC Documents Identify Sample as from a CT Source ^b^?	Is it a Sub- Slab Sample? ^b^	Does TC Consider Sample Area “Non-Hazardous,” thus Not to be Removed ^c^ (No Hotpot/Drain)?	Depth in Feet	CT (µg/m^3^) ^a^	Times above 10^−6^ CT Health-Protective/Screening Level (0.067 µg/m^3^) ^d^? (÷col 7 by 0.067)
NMSD3-113	S	N	N	Y	113	28,400	424,000
NMSD3-84	S	N	N	Y	84	24,300	363,000
NMSD3-150	S	N	N	Y	150	20,600	307,463
NMSD3-150	S	N	N	Y	150	18,500	276,119
NMSD2-150	N	N	N	Y	150	13,200	197,015
NMSD2-130	N	N	N	Y	130	12,900	193,000
NMSD2-150	N	N	N	Y	150	9830	146,700
NMSD3-60	S	N	N	Y	60	8390	125,224
NMSD1-85	S	N	N	N	85	7530	112,388
NMSD1-99	S	N	N	N	99	5950	90,806

^a^ [10] (Appendix D, Table 3) and [10] (Appendix E) show all 172, all one-time/temporary-well samples; 60% violate PCE health-protective/screening levels; see Supplementary Materials S1 (Table S1 and Figure S2). ^b^ Assessors identify only 9 VOC-CT secondary sources: V2, V3, V8, V10, V12, V18, NMSC6, NMSC8, and NMSC14, despite many violations like those above [6] (Figures 9 and 10), [10]; see Supplementary Materials S1 (Figures S2–S6). Secondary sources [21] (p. 5) are direct-exposure starting points, reservoirs sustaining contaminant plumes in groundwater/air/soil gas, [56] (p. 34). ^c^ [58] (p. 42). ^d^ DTSC [60], used in [6,10].

**Table 3:** Soil-gas-VOC-testing violations at the former NOTSPA, Pasadena, California (P) and the former heavy-manufacturing site, Monrovia, California (M).

	DTSC Requires ^a^ Testing	TC Violates These Requirements, Given
R1	At/near contaminant sources	P: No site-wide sampling, ^b^ no source tests for 33 of 35 site pollutants. ^c^ M: Sources unknown; no sitewide sampling (21 samples on 10 acres). ^aa^
R2	With multi-season/ sample, same-semi- permanent-well tests	P and M: Only one-time samples from temporary wells. ^b,aa^
R3	Under steady-state conditions	P and M: No steady-state, toxin-nonmigration tests, given R2above. ^b,aa^
R4.1	Per CA Soil-Gas Advisory: Test to 3D-contaminant-plume extent.	P and M: See R1; no offsite tests, no tests 180 ft above groundwater. ^b^ M: soil-gas VOC samples were all 5-feet subsurface. ^aa^ P: property-line toxins are up to 80,000 times above screening or health- protective levels (V10-5). ^b^
R4.2	Per Soil-Gas Advisory: Give 2 center-subslab samples/building	P: 86% of buildings had no required subslab samples. ^b^ M: 43% of buildings had no required subslab samples. ^bb^
R4.3	Per Soil-Gas Advisory: Provide maximum-level concentrations.	No maxima given, as sources are unknown; VOCs migrate downward, but sites had no tests within 180+ feet above groundwater. ^b,aa^
R5	With detection limits as sensitive as screening levels	Detection limits were up to 1000 times (P), ^b^ and 435 times (M), ^cc^ less sensitive/protective than required screening levels; see Table 4.
R6	Provide all- contaminant/depth isoconcentration maps	P: Maps for only 2 of 35 VOCs, only for 5–15 feet subsurface. ^c^ M: No isoconcentration maps. ^dd^

^a^ [21] (p. 17); [48] (pp. 7–9, 17, 19). ^b^ P: Table 1 and Table 2 above; Supplementary Materials S1, Table S1 and Figure S2. ^c^ P: Supplementary Materials S1, (Figures S3–S6). ^aa^ M: [62] (Table 1). ^bb^ M: Supplementary Materials S1 (Figure S13). ^cc^ M: [62]. ^dd^ M: [62].

**Table 4:** Re R5, Pasadena and Monrovia, California waste-site testing used soil-gas VOC method-detection/reporting limits (MDLs) that fail required screening levels (SLs). The sites will be redeveloped for 986 residential apartments.

	Required SLs ^a^, µg/m^3^	Pasadena MDLs ^b^, µg/m^3^	Monrovia MDLs ^c^, µg/m^3^
**carbon tetrachloride**	0.067	20	not given
**chloroform**	0.12	20	not given
**dibromochloromethane**	0.13	20	not given
**per/tetrachloroethylene**	0.46	20	20
**trichloroethylene**	0.48	20	20

^a^ Stricter SLs take precedence, given federal/state differences. CT/PCE SLs, per California [60]; others from US EPA [63]. ^b^ Supplementary Materials S1 (Table S1). ^c^ [62].

**Table 5:** Violations of testing requirements, former missile-testing site, Canoga Park, California (CP) and former landfill, Pacoima, California (P).

	DTSC Requires ^a^ Testing	TC Violates These Requirements, Given
R1	At/near contaminant sources	CP: No sitewide soil-gas survey, no location of TCFM sources. ^b^ P: No evidence of sitewide testing/source identification. ^c^
R2	With multi-season/sample, semi- permanent-well tests	CP: Only semi-permanent wells, no multi-season tests. ^d^ P: 7 semi-permanent probes/33 acres, inadequate multi-season tests. ^e^
R3	Under steady-state conditions	CP: No steady state, ^b,d^ given R2 and increasing groundwater toxins. ^f^ P: No: known steady state/wells at site S/SE/center. ^e^
R4.1	Per CA Soil-Gas Advisory: Test to 3D-toxin-plume extent.	CP and P: See R1. No testing of toxin-plume boundaries. ^g,c^
R4.2	Per Soil-Gas Advisory: Give 2 center-subslab samples/building.	CP: No buildings had required subslab sampling. ^i^ P: No evidence of required subslab sampling. ^c^
R4.3	Per Soil-Gas Advisory: Provide maximum-level toxin concentrations.	CP and P: No sitewide sampling, no location of maximum-level concentrations. See R1.
R5	With method-detection limits as sensitive as screening levels	CP: Detection limits ^g^ 500 times less protective than required; ^h^ assessor-calculated TCFM SL = 562 times > DTSC’s approved SL. ^i^ P: Online database has no required sampling reports/logs/surveys. ^j^
R6	Provide all-contaminant/depth isoconcentration maps	CP: Given R1, no complete isoconcentration maps. ^b,f,k^ P: No isoconcentration maps, no sampling reports provided. ^j^

^a^ [21] (p. 17). [48] (pp. 7–8, 17, 9). ^b^ CP: [73]. ^c^ P: [68,69,70,71]. ^d^ CP: [72] (p. 2). ^e^ P: [70,74]. ^f^ CP: [75]. ^g^ CP: [72,76,77]. ^h^ CP; [10] (Appendix D, Table 3). ^i^ CP: [72]; see Supplementary Materials S1 (Figure S14; (for SLs) Table S1). ^j^ P: [54,78]. ^k^ [79]; see Supplementary Materials S1 (Figures S15 and S16).

**Table 6:** **Violations of testing requirements**, former Santa Fe Railyards, Boyle Heights, California.

	DTSC Requires ^a^ Testing	TC Violates These Requirements, Given
R1	At/near contaminant sources	No evidence of source locations, no sitewide-soil-gas survey; see R2. ^b^
R2	With multi-season/sample semi- permanent-well tests	Only 16 semi-permanent wells for 18-acre site, 2013–2017; no full-site testing. ^b^
R3	Under steady-state conditions	No steady-state: Migrating VOCs, increasing soil-gas well VOCs. ^c^
R4.1	Per CA Soil-Gas Advisory: Test to 3D-contaminant- plume extent.	No testing to 3D-contaminant-plume extent. ^c^ (See R1–R3.)
R4.2	Per Soil-Gas Advisory: Provide 2 central-subslab samples/ building.	33% of buildings had no required subslab samples. ^b^
R4.3	Per Soil-Gas Advisory: Provide maximum-level concentrations.	No maximum concentrations provided. ^c^ (See R3.)
R5	With method-detection limits as sensitive as screening levels	Detection limits are 200+ times > required screening levels. ^c,d^
R6	Provide all-contaminant/ depth isoconcentration maps.	No isoconcentration maps, no evidence of sitewide sampling. ^b,c^

^a^ [21] (p. 17). [48] (pp. 7, 8). [48] (pp. 9, 17). ^b^ [79]; see Supplementary Materials S1 (Figures S14 and S17). ^c^ [79]; see Supplementary Materials S1 (Table S2). ^d^ [82].

**Table 7:** VOC-testing violations (V) or no violations (NV), 5 California redevelopments of hazardous sites.

	Pasadena, Apartment Homes	Monrovia, Apartment Homes	Canoga Park, Business-rental Units	Pacoima, Business- Rental Units	Boyle Heights, Business-Rental Units
6 DTSC Requirements for Soil-Gas VOC Testing:	Former Naval Ordnance Test Station ^a^	Former Manufacturing Site ^b^	Former Nuclear-Missile Testing, Development, Manufacturing Site ^c^	Former Landfill ^c^	Former Railyards ^d^
At/near sources	V	V	V	V	V
With multi-season, multi- sample, same semi-permanent well tests	V	V	V	NV	V
Under steady-state conditions	V	V	V	V	V
Follow CA Soil-Gas Advisory	V	V	V	V	V
Using method-detection limits at/below screening levels	V	V	V	V	V
Providing all-depth, all- contaminant isoconcentration maps	V	V	V	V	V

^a^ See Table 1, Table 2, Table 3 and Table 4. ^b^ See Table 3 and Table 4. ^c^ See Table 5. ^d^ See Table 6.

**Table 8:** Do NOTSPA documents show “all of the impacted soils” will “be removed,” as TC claims [91]?

NOTSPA Chlorinated VOCs, Industrial Solvents	US EPA, DTSC Residential Soil-Gas VOC Cleanup Levels, ^b^ 10^−6^ Target Cancer Risk ^c^	TC’s Allowable Shallow Soil- Gas VOC Cleanup levels ^d^	TC’s Allowable VOC Levels Are How Much Less Protective than DTSC’s 10^−6^ Target-Risk Cleanup Levels? (Divide Column 3 by Column 2)
Trichloroethylene, TCE ^a^	0.48 µg/m^3^	12,400 µg/m^3^	25,833 times less protective
Perchloroethylene, PCE	0.46 µg/m^3^	5470 µg/m^3^	11,891 times less protective
Carbon tetrachloride, CT	0.067 µg/m^3^	705 µg/m^3^	10,522 times less protective

^a^ Brief/airborne exposure to 0.5 µg/m^3^ TCE may produce fetal cardiac defects [41,42]. ^b^ [10] (Appendix D, Table 3); [60,63]. ^c^ [10,60]. ^d^ [58] (p. 37).

## Data Availability

All data are in public documents, most of which are on the internet, and all these documents are cited in the paper. This paper produced no new, original data.

## Supplementary Materials

The following supporting information can be downloaded at: https://www.mdpi.com/article/10.3390/ijerph19137679/s1. Supplementary materials include S1 and S2. S1 is a set of figures and tables from cited references, broken into three parts (1a, 1b, 1c), because the entire file is too large to be sent by email. Otherwise S1 would have to be sent as a google drive link. The text cites these tables and figures and indicates their availability in S1. S2, likewise cited in the text, is a .docx file. It contains 8 sections outlining the author’s further technical details or explanations of material in the text. It also includes all references cited either in the text (references [1,2,3,4,5,6,7,8,9,10,11,12,13,14,15,16,17,18,19,20,21,22,23,24,25,26,27,28,29,30,31,32,33,34,35,36,37,38,39,40,41,42,43,44,45,46,47,48,49,50,51,52,53,54,55,56,57,58,59,60,61,62,63,64,65,66,67,68,69,70,71,72,73,74,75,76,77,78,79,80,81,82,83,84,85,86,87,88,89,90,91,92,93,94,95,96,97,98,99,100,101,102,103,104,105,106,107,108,109,110,111,112,113,114,115,116,117] or in S1 and S2 (references [1,2,3,4,5,6,7,8,9,10,11,12,13,14,15,16,17,18,19,20,21,22,23,24,25,26,27,28,29,30,31,32,33,34,35,36,37,38,39,40,41,42,43,44,45,46,47,48,49,50,51,52,53,54,55,56,57,58,59,60,61,62,63,64,65,66,67,68,69,70,71,72,73,74,75,76,77,78,79,80,81,82,83,84,85,86,87,88,89,90,91,92,93,94,95,96,97,98,99,100,101,102,103,104,105,106,107,108,109,110,111,112,113,114,115,116,117,118,119,120,121,122,123,124,125,126,127,128,129,130,131,132,133,134,135,136,137,138,139,140,141,142,143,144,145,146,147,148,149,150,151,152,153,154,155,156,157,158]. The author uses S1 and S2 mainly so that the text itself is more linear and readable, thus more accessible to experts from a wide variety of fields, and so that technical details can be outlined, although they are not essential to the main line of argument of the paper itself, but underlie the paper. Within S2’s eight sections (Sections S2.1–S2.8) outlines the main types of US hazardous-waste sites, and many circumstances affecting their remediation. Section S2.1 explains why most US hazardous-waste sites have neither been assessed nor remediated. Section S2.2 explains why many hazardous-waste-site threats are unknown, including that their testing/remediation tends to be privatized. Section S2.3 explains why hazardous-waste cleanup typically does not correct environmental injustice. Section S2.4 explains US EPA’s 2016 Weight of Evidence (WoE) method and why it is essential to evaluating hazardous-waste sites. Sections S2.5 and S2.6, respectively, outline systematic, transparent, fully reproducible WoE strategies to find federal and state of California regulatory guidance on vapor-intrusion (VI) testing—and hazardous-waste sites whose vapor-intrusion-testing documents to evaluate. Section S2.7 explains why government guidance can impose requirements, although it is not part of regulations. Section S2.8 outlines TC’s violations of 6 DTSC-mandated, cancer-calculation rules and explains why DTSC requires Human Health Screening Evaluations(HHSEs) to use maximum contaminant concentrations. (TC violates both these rules and the HHSE requirement).

## Abbreviations

BAD	Brownfields Acquisition and Development
CAL-EPA	California Environmental Protection Agency
CARB	California Air Resources Board
TC	Coldwell Banker Richard Ellis (TC) Group, Inc.
CERCLA	Comprehensive Environmental Response, Compensation, and Liability Act (known also as “Superfund”)
CT	Carbon tetrachloride
DTSC	California Department of Toxic Substances Control
DUE	Data-usability evaluation
EASI	Environmental Assets Services Incorporated
EPA	United States Environmental Protection Agency
MDL	Method-detection limit
N	North
NOTSPA	US Naval Ordnance Testing Station, Pasadena, California
NW	Northwest
PCBs	Polychlorinated biphenyls
PCE	Perchloroethylene
PFAS	Perfluorinated substances
R	Requirement
RCRA	United States Resource Conservation and Recovery Act
RL	Reporting limit
S	South
SE	Southeast
SL	Screening level
SSFL	Atomics International/US Atomic Energy Commission Santa Susana Field Lab
TC	Trammell Crow Company
TCE	Trichloroethylene
UCLA	University of California, Los Angeles
US	United States
USGS	United States Geological Survey
VOC	Volatile organic compound
WoE	Weight-of-evidence
**Abbreviations from Referencs**	
CHEJ	Center for Health, Environment and Justice
CIWQS	California Integrated Water Quality System
CLA-DPW	City of Los Angeles Department of Public Works Bureau of Sanitation
CPPCD	City of Pasadena Planning and Community Development
DTSC-HERO	California Department of Toxic Substances Control, Human and Ecological Risk Office
ELC-ABA	Environmental Litigation Committee of the American Bar Association
GAO	US Government Accountability Office
GTI	Groundwater Technology Incorporated
LACPC	Los Angeles City Planning Commission
LACRA	Community Redevelopment Agency of the City of Los Angeles
LARWQCB	Los Angeles Regional Water Quality Control Board
MIG	Moore, Iacofano, and Goltsman Inc.
NAVFAC	US Naval Facilities Engineering Command
NJDEP	New Jersey Department of Environmental Protections
NYOPH	New York Office of Public Health
OEHHA	California Office of Environmental Health Hazard Assessment
RCC	Retail Compliance Center
SFRWQCB	San Francisco Regional Water Quality Control Board
Shaw	Shaw Environmental and Infrastructure
SWRCB	California State Water Resources Control Board
TCR, Monrovia, and MIG	Trammell Crow Residential, Monrovia, and Moore, Iacofano, and Goltsman
TCR	Trammell Crow Residential
