# Supplementary material for: Does Hazardous-Waste Testing Follow Technical Guidance, Thus Help Protect Environmental Justice and Health?
Source: Int J Environ Res Public Health. 2022 Jun 23;19(13):7679. doi: 10.3390/ijerph19137679 (PMC9265884; doi:10.3390/ijerph19137679)

Contents of Supplemental File S1a

Pages

Figures.. 1-20
Figure S1…... 2
Figure S2…... 3
Figure S3…... 4
Figure S4…... 5
Figure S5…... 6
Figure S6…... 7
Figure S7…... 8
Figure S8…... 9
Figure S9…... 10

Contents of Supplemental File S1b

Figure S10…... 11
Figure S11…... 12
Figure S12…... 13
Figure S13…... 14
Figure S14…... 15
Figure S15…... 16
Figure S16…... 17
Figure S17…... 18
Figure S18…... 19

Contents of Supplemental File S1c

Tables.. 20-33
Table S1…... 21-23
Table S2…... 24-30
Table S3…... 31
Table S4…... 32
Table S5…... 33

FIGURES

Figure S1. "Hot-Spot" Sampling Locations at Former Naval Ordnance Test Station, Pasadena, CA [6, 59], Figure 7.

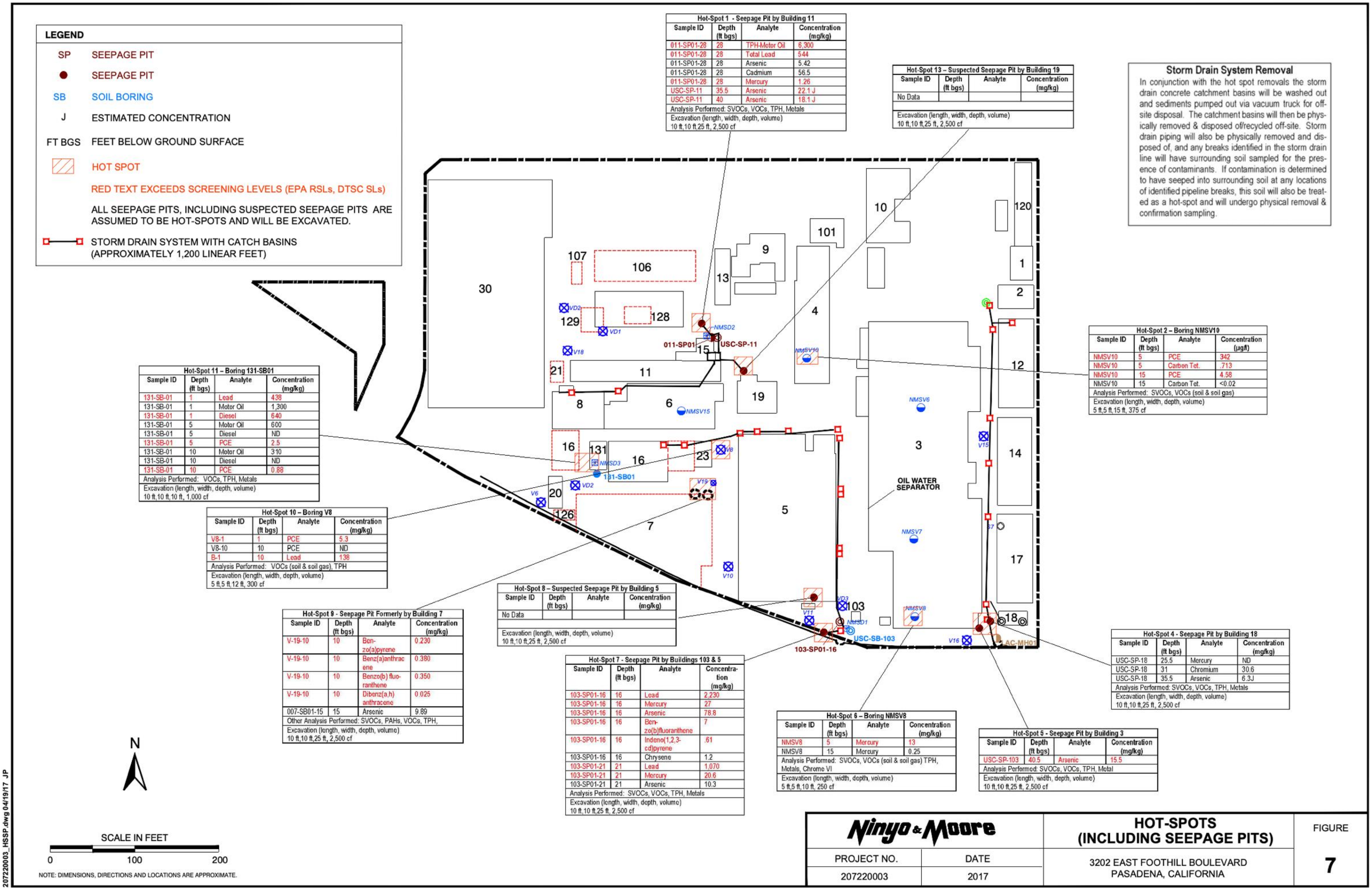

Figure S2. Environmental Sampling Locations at Former Naval Ordnance Test Station, Pasadena, CA [6, 58, 59], Figure 4.

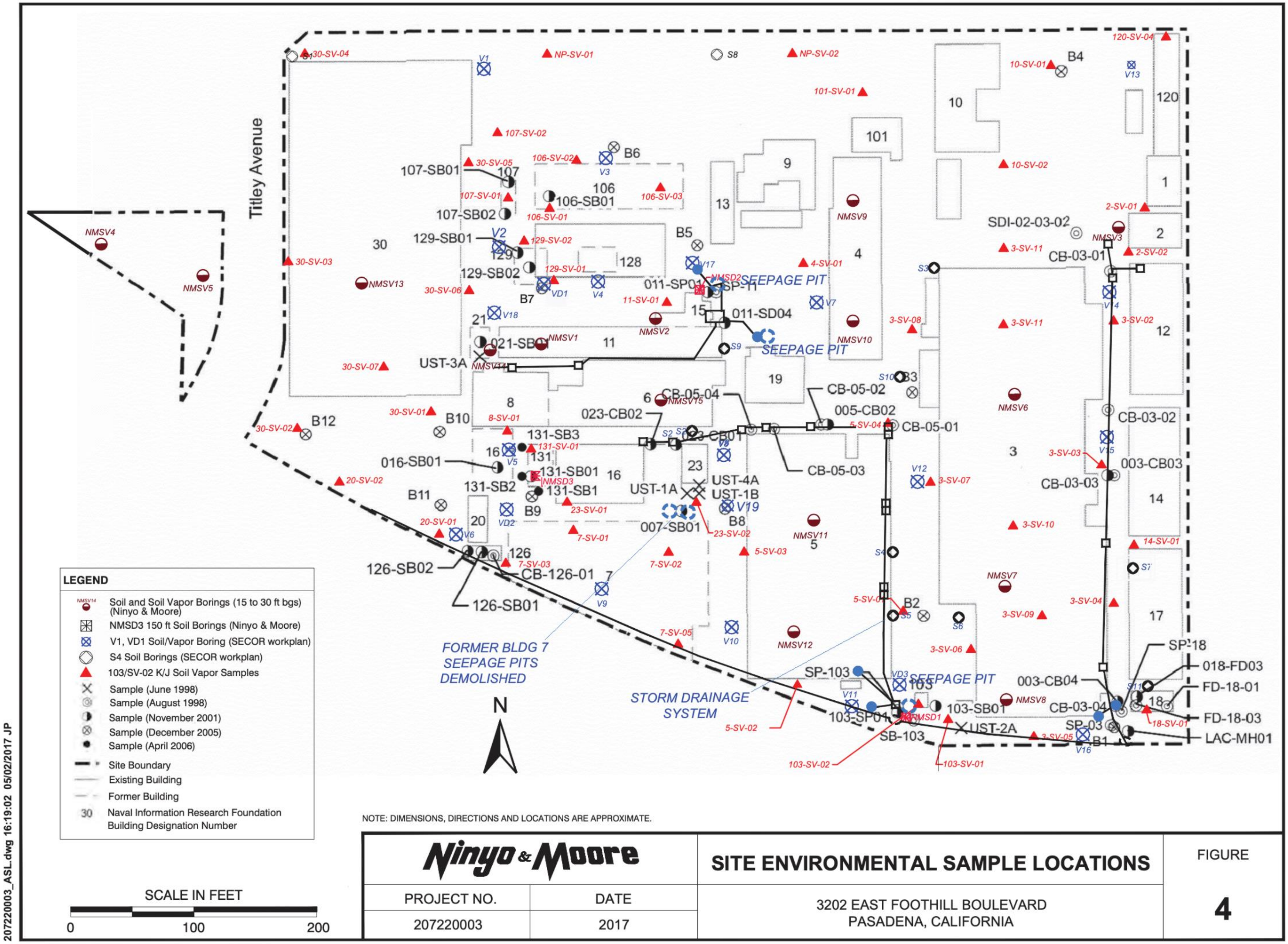

Figure S3. PCE Soil Gas Isoconcentration Map at Former Naval Ordnance Test Station, Pasadena, CA [6, 59], Figure 9.

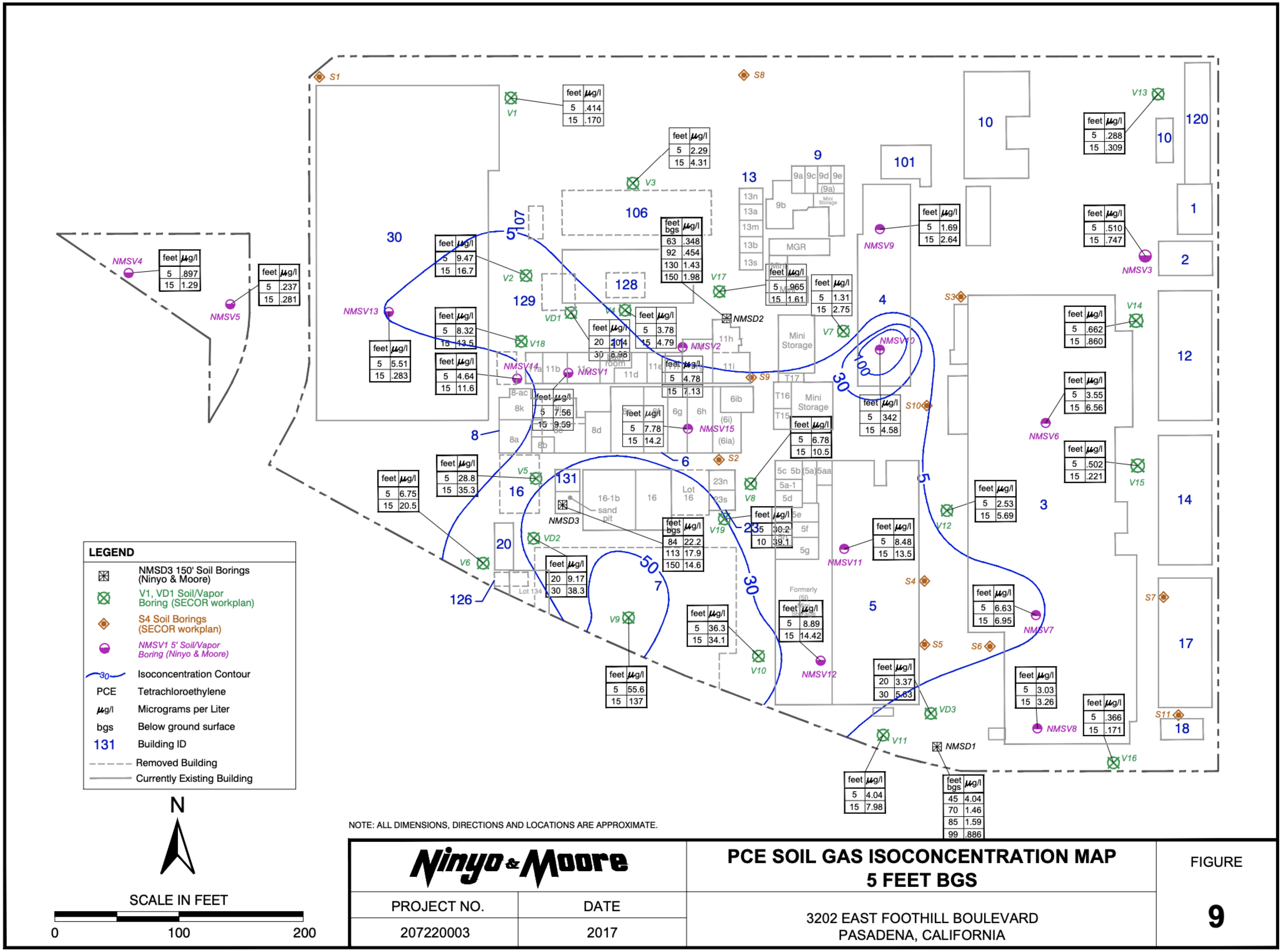

Figure S4. PCE Soil Gas Isoconcentration Map at Former Naval Ordnance Test Station, Pasadena, CA [6, 59], Figure 10.

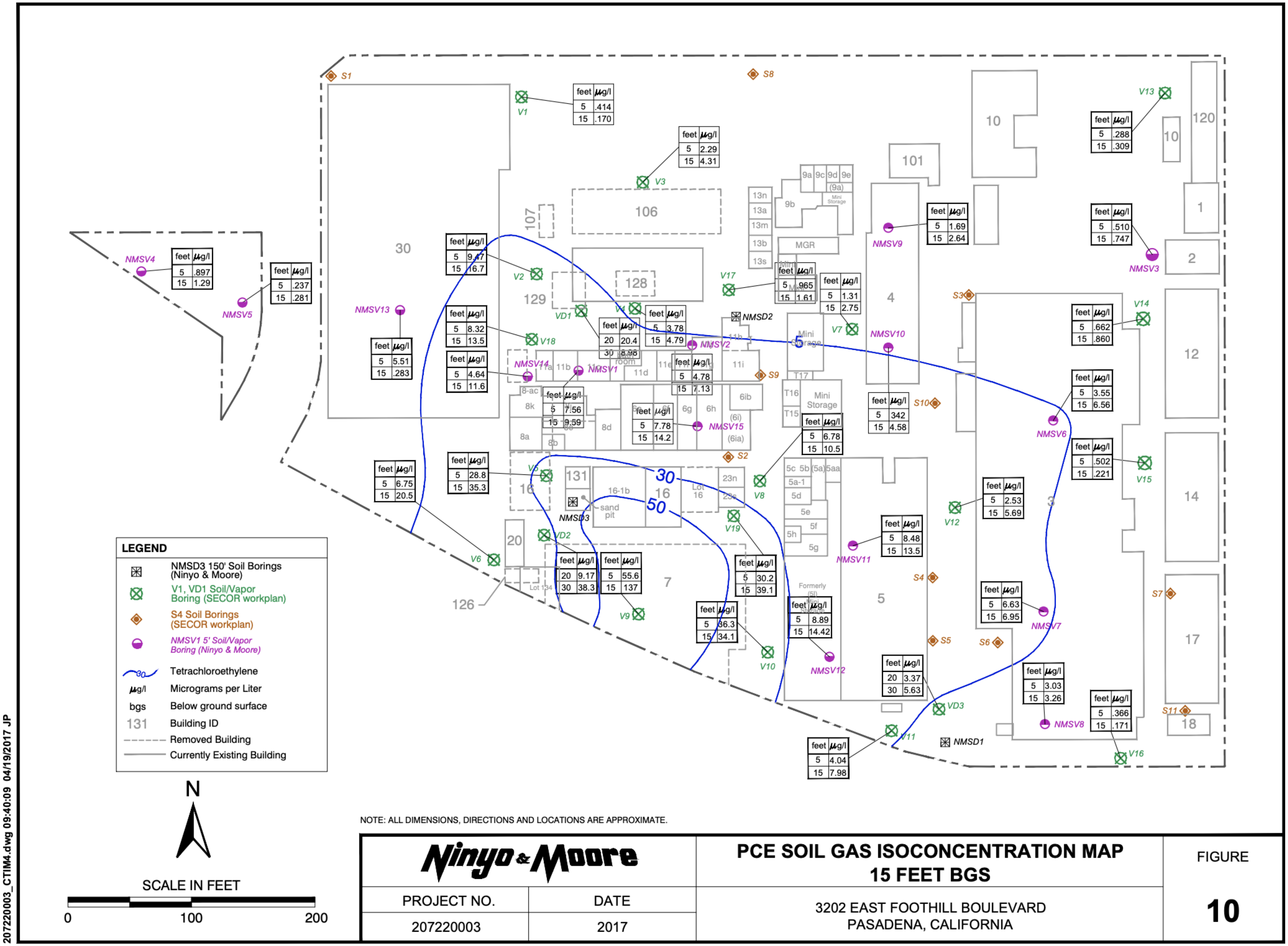

Figure S5. CT Soil Gas Isoconcentration Map at Former Naval Ordnance Test Station, Pasadena, CA [6, 59], Figure 11.

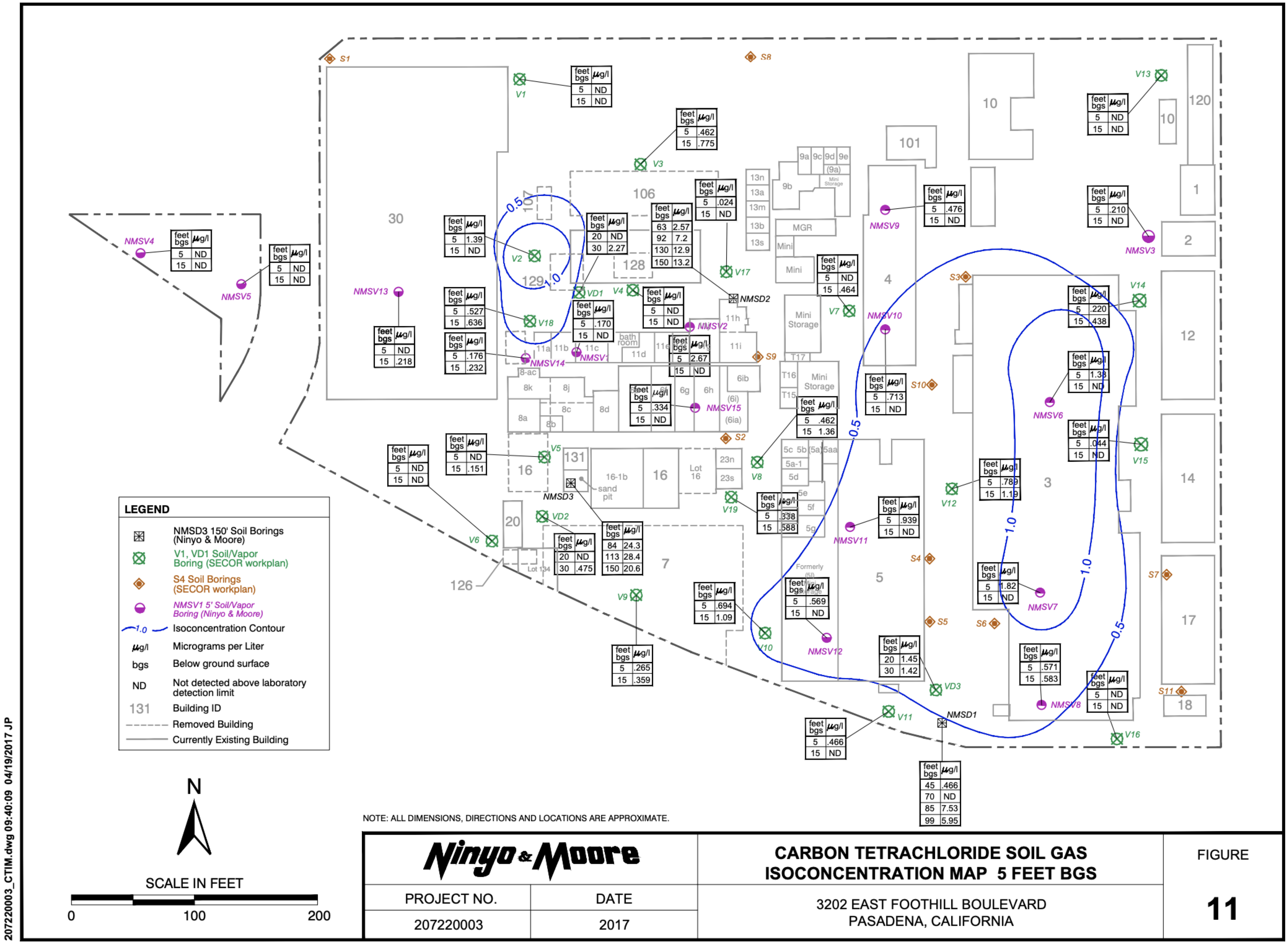

Figure S6. CT Soil Gas Isoconcentration Map at Former Naval Ordnance Test Station, Pasadena, CA [6, 59], Figure 12.

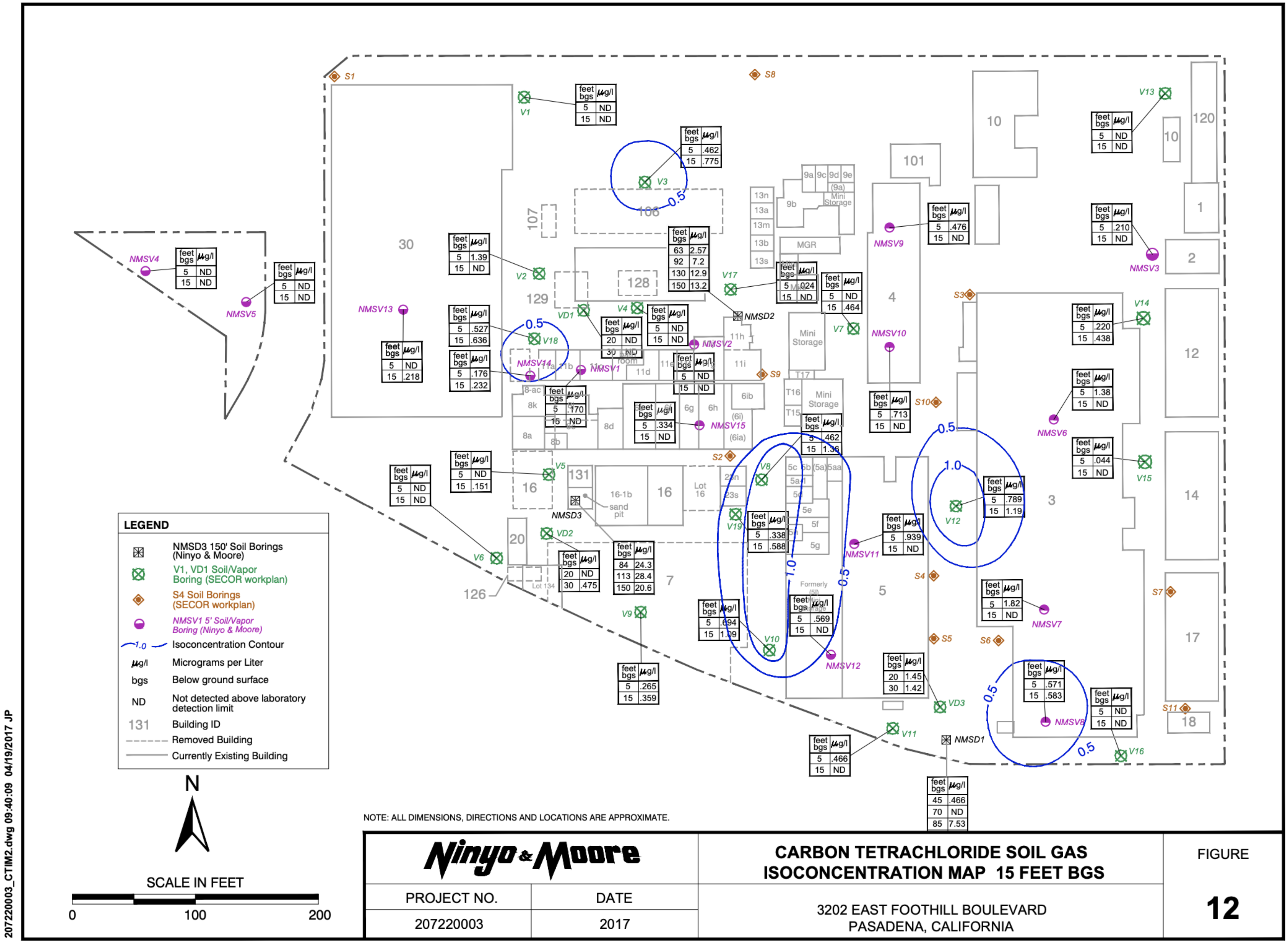

Figure S7. Site Location at Former Naval Ordnance Test Station, Pasadena, CA [59], Figure 1.

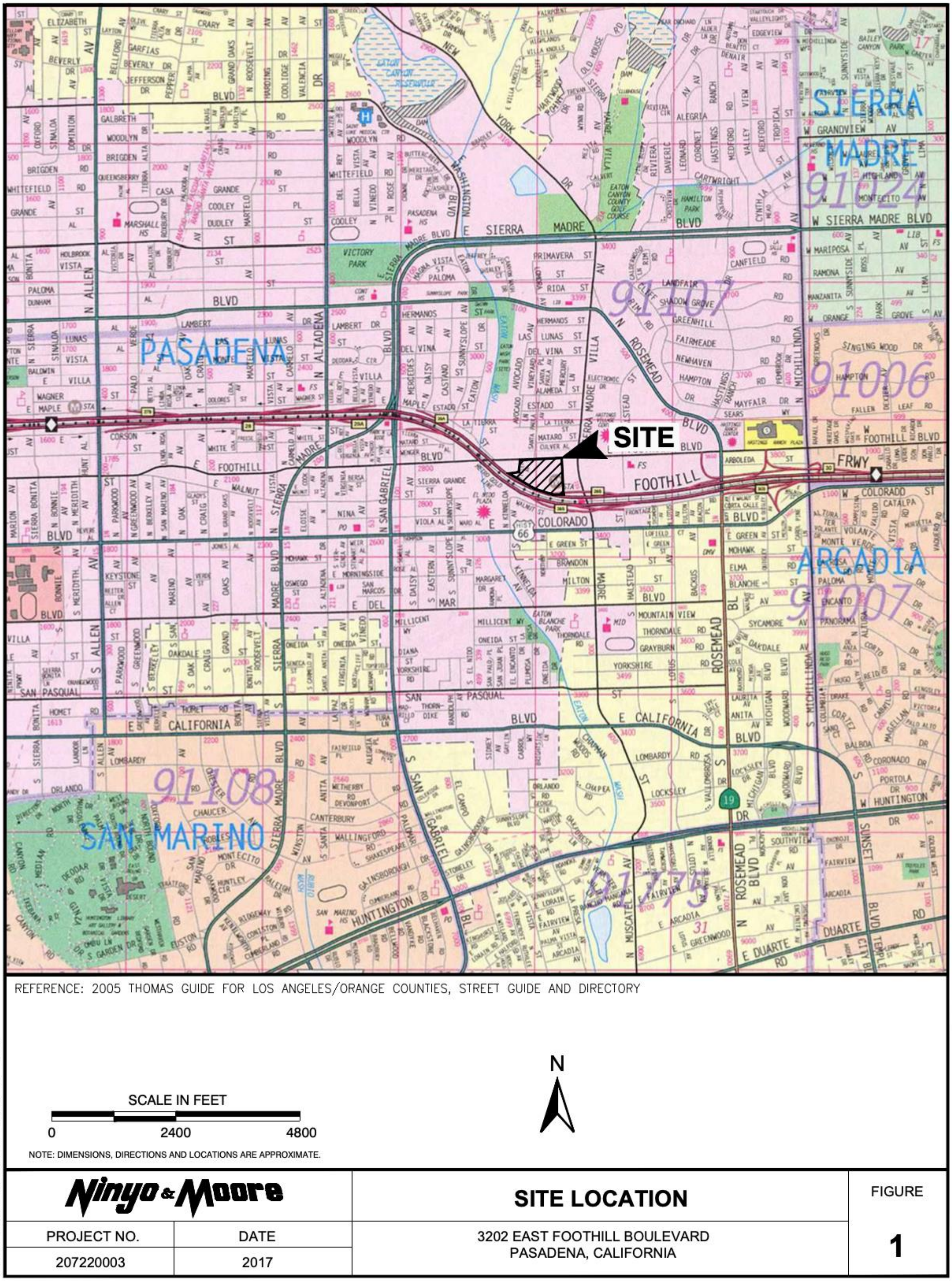

207220003_SL.dwg 09:37:05 04/19/2017 G□□JP

Figure S8. Site Plan at Former Naval Ordnance Test Station, Pasadena, CA [59], Figure 2.

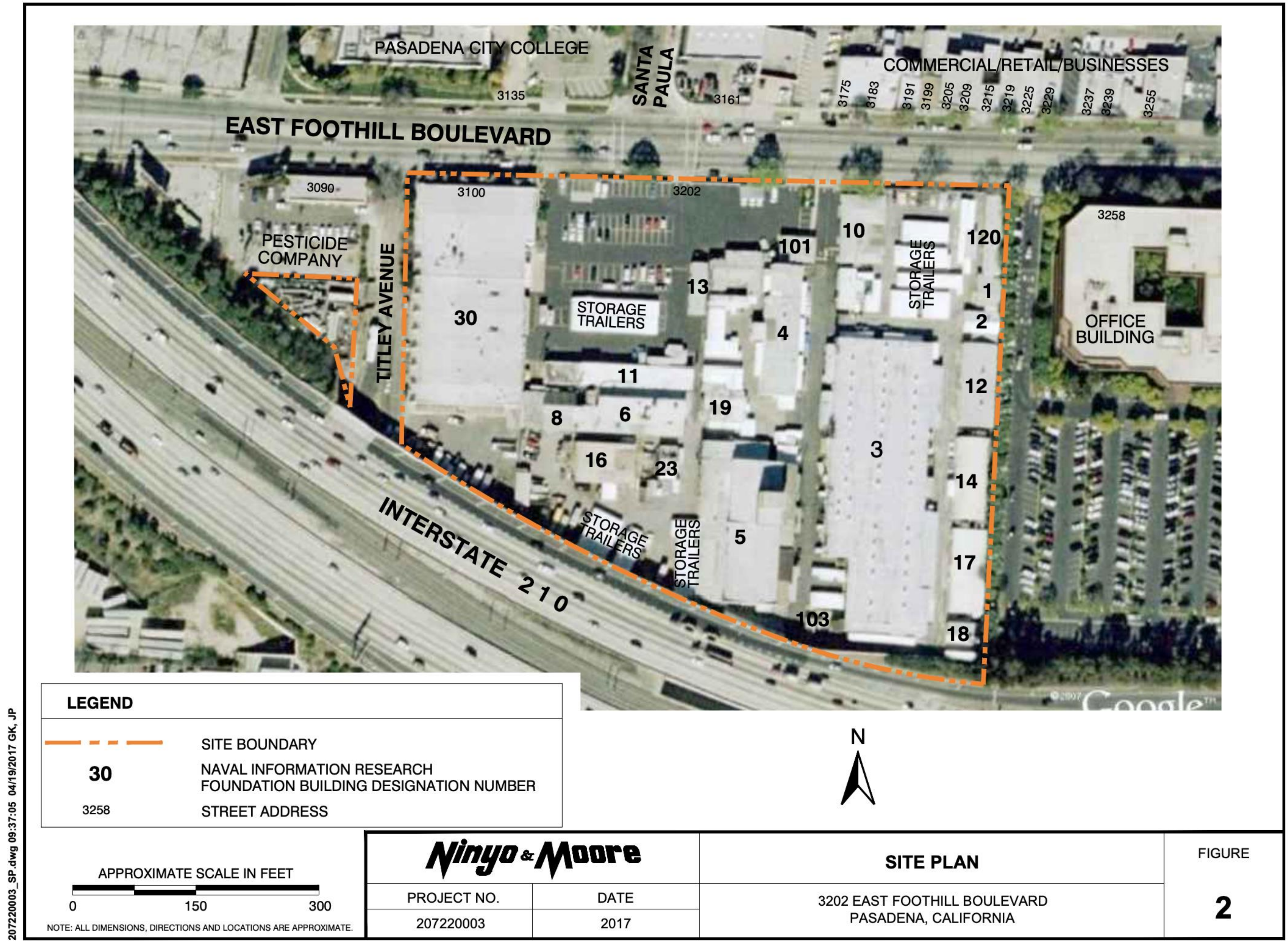

Figure S9. Past Military and Current Site Use at Site at Former Naval Ordnance Test Station, Pasadena, CA [59], Figure 3.

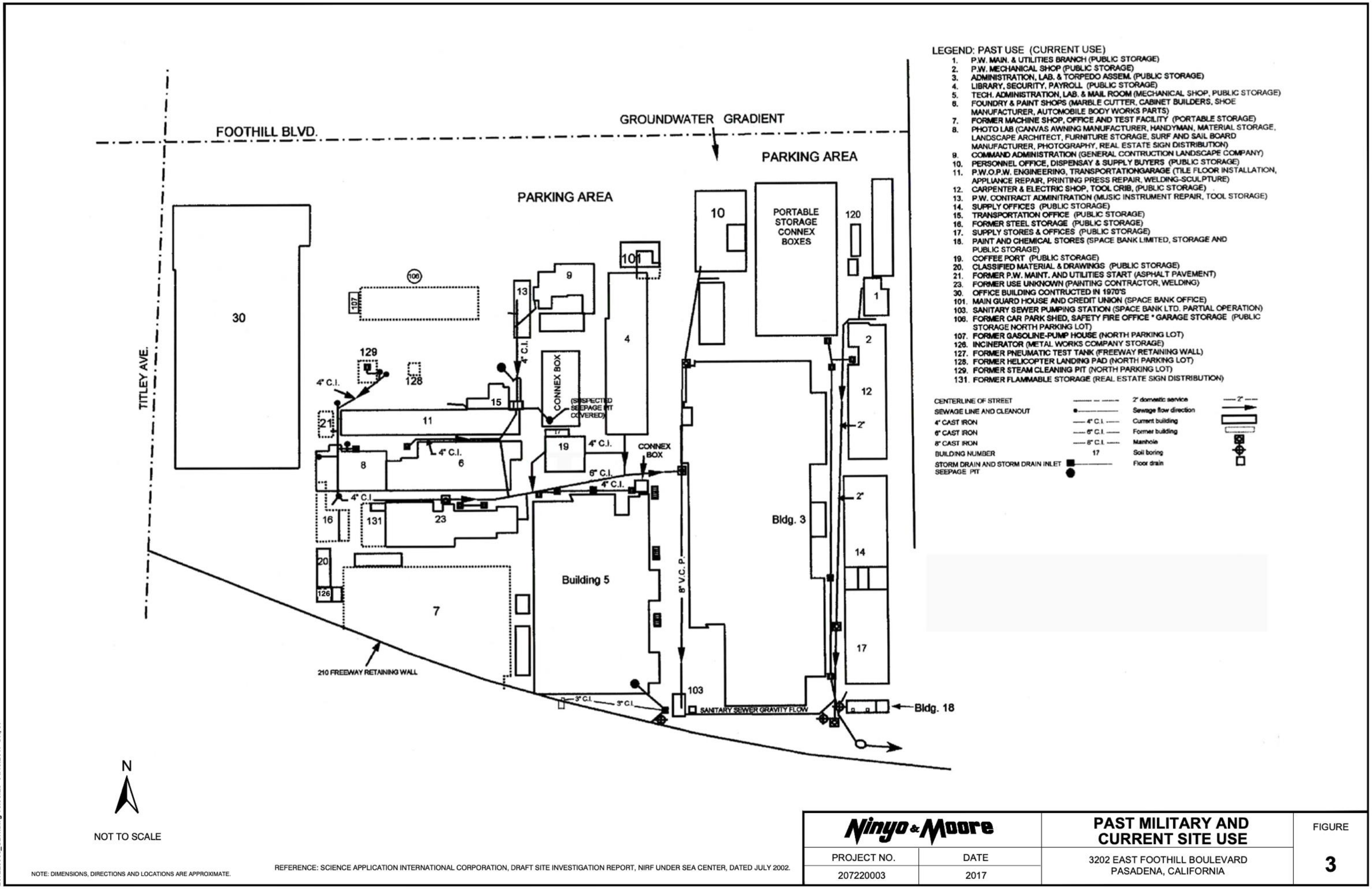

Supplement: Supplementary file 1 [file ijerph-19-07679-s001.zip › 6-2-22-ksf-Supplemental File S1a.pdf]
