# Supplementary material for: Does Hazardous-Waste Testing Follow Technical Guidance, Thus Help Protect Environmental Justice and Health?
Source: Int J Environ Res Public Health. 2022 Jun 23;19(13):7679. doi: 10.3390/ijerph19137679 (PMC9265884; doi:10.3390/ijerph19137679)

Contents of Supplemental File S1b

Pages
Figure S10…... 11
Figure S11…... 12
Figure S12…... 13
Figure S13…... 14
Figure S14…... 15
Figure S15…... 16
Figure S16…... 17
Figure S17…... 18
Figure S18…... 19

Figure S10. Storm Water Seepage Pit Diagram at Former Naval Ordnance Test Station, Pasadena, CA [59], Figure 5.

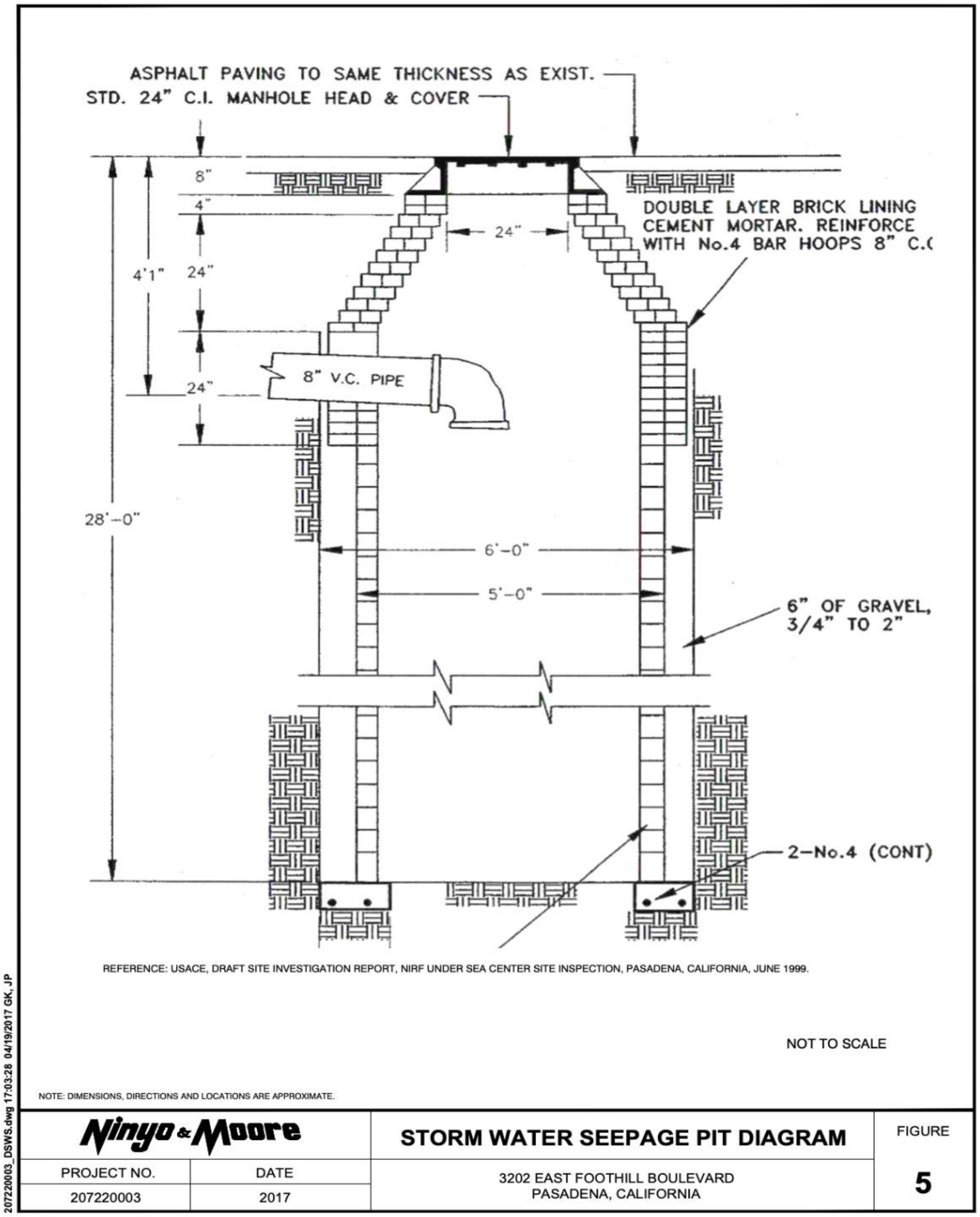

Figure S11. TPH and PAHS Sampling Locations at Former Naval Ordnance Test Station, Pasadena, CA [59], Figure 6.

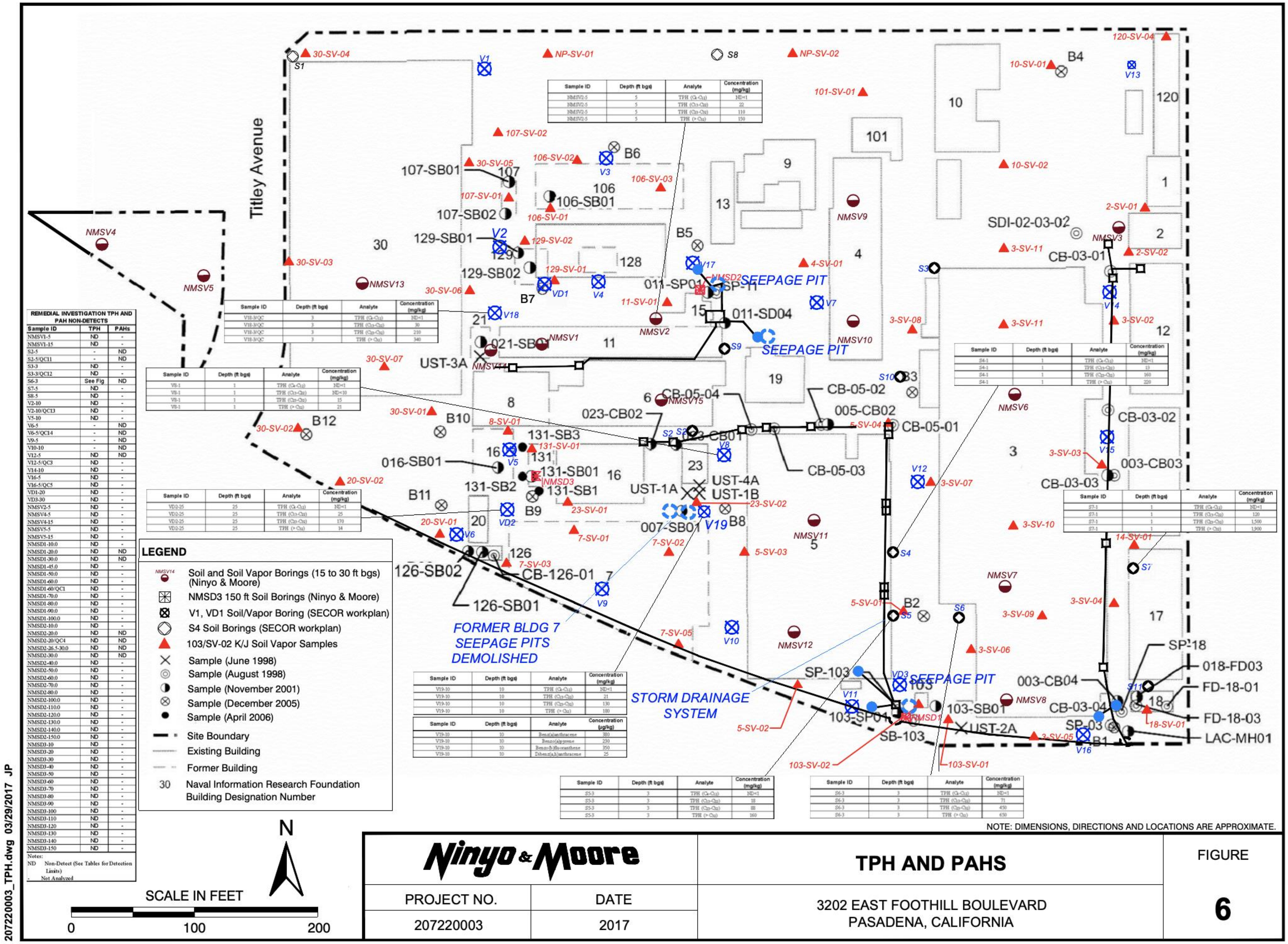

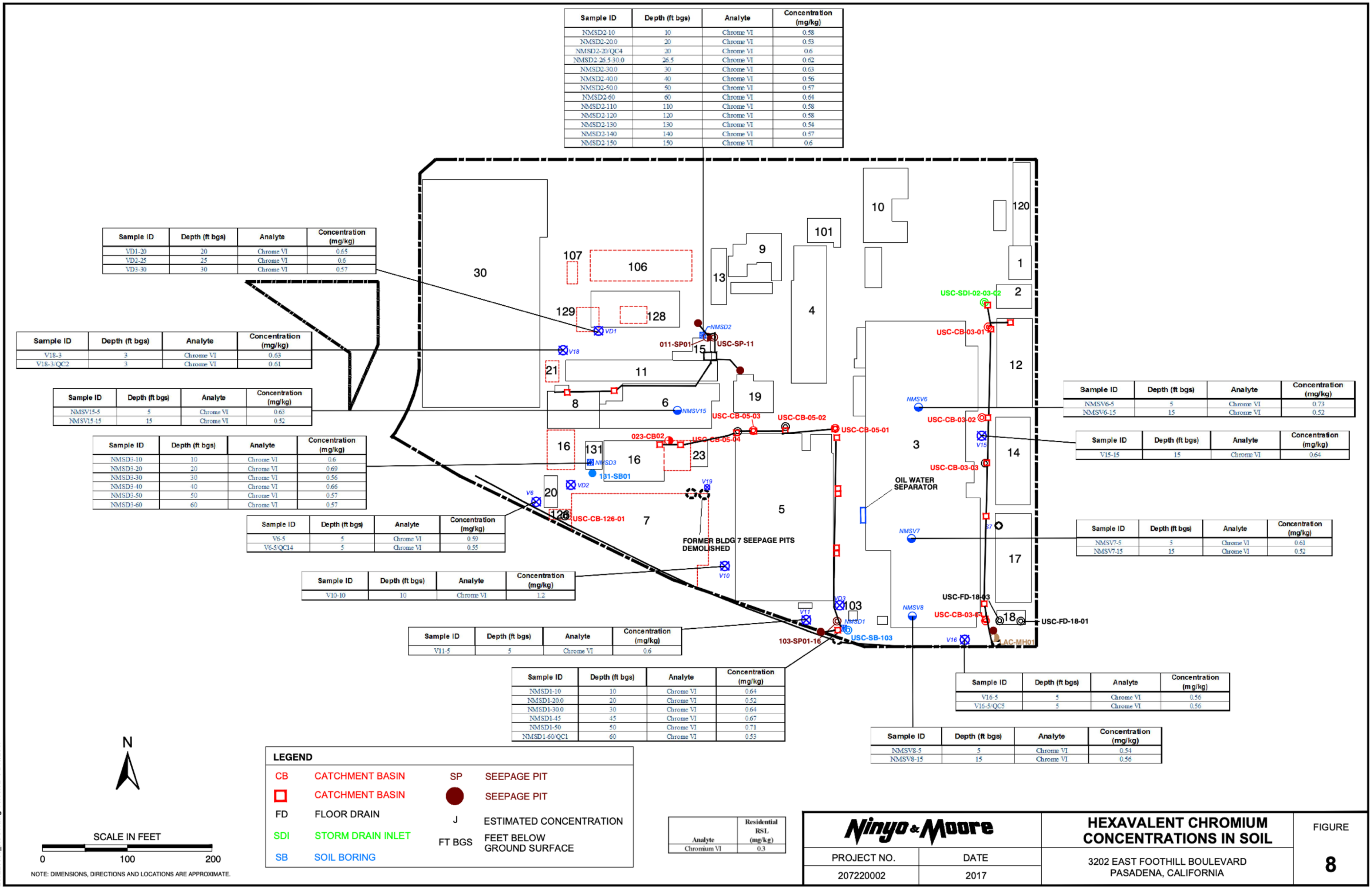

Figure S12. Hexavalent Chromium Concentrations in Soil at Former Naval Ordnance Test Station, Pasadena, CA [59], Figure 8.

Figure S13. Boring Locations at Former Industrial Site, Monrovia, CA [83], Figure 3.

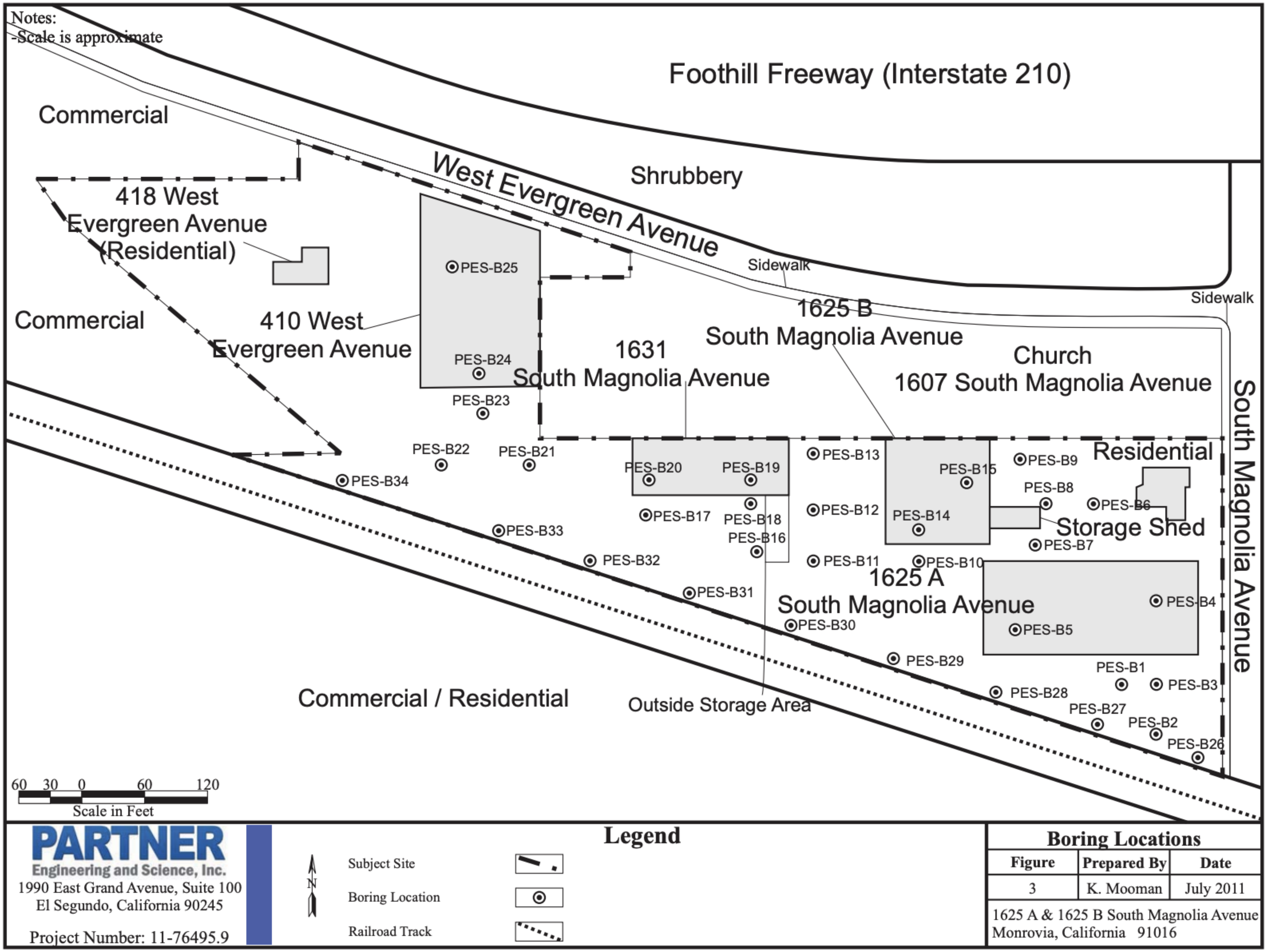

Figure S14. Soil Vapor Extraction System, Soil Vapor Extraction Well and Semi-Permanent Soil Vapor Probe Layout at Former Santa Fe Railyard, Boyle Heights, CA [79], Figure 7.

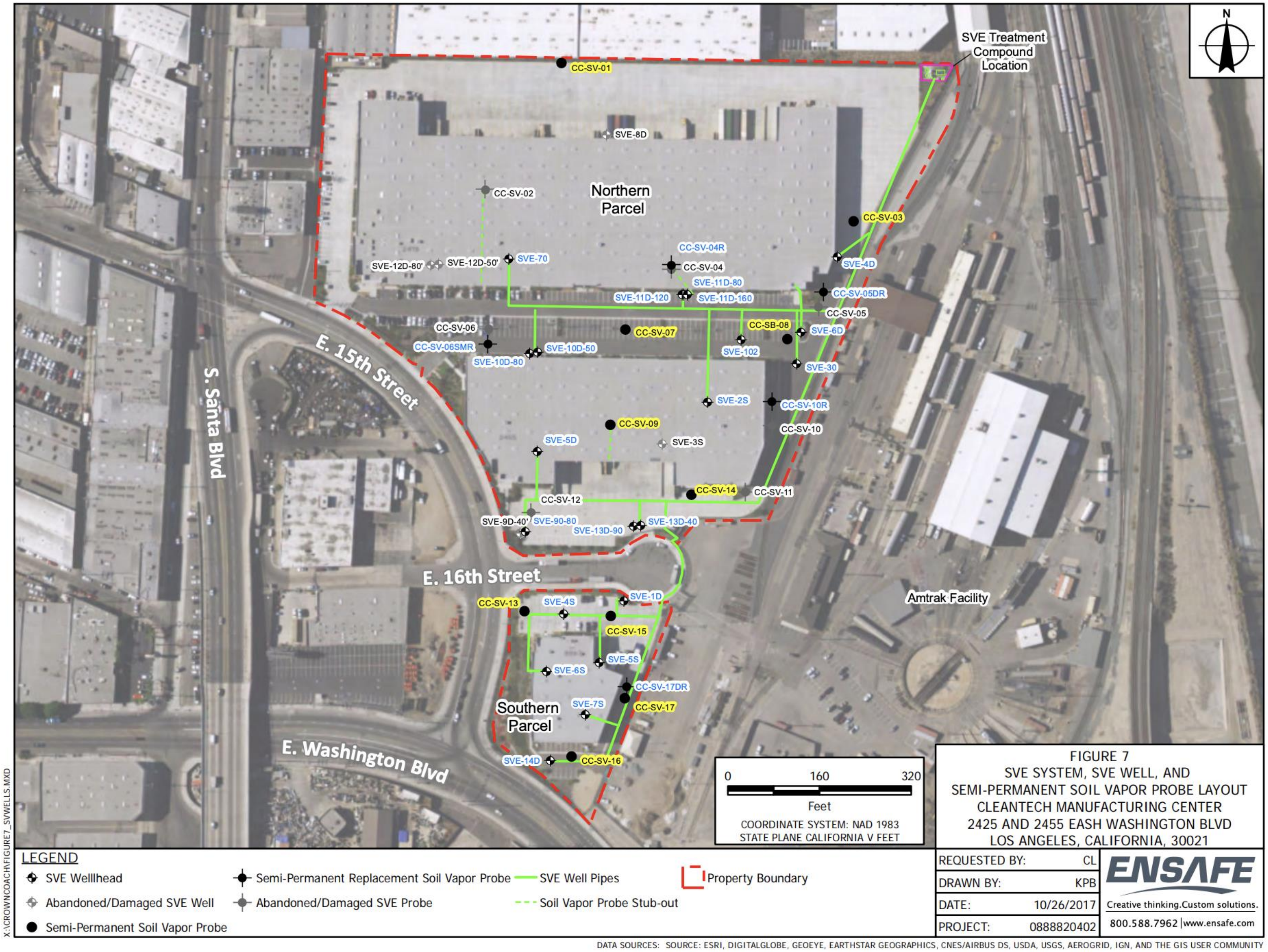

Figure S15. Soil Gas Analytical Results for Freon 11 at Former Raytheon/Atomics International Nuclear Weapons Site, Canoga Park, CA [72], Figure 4.

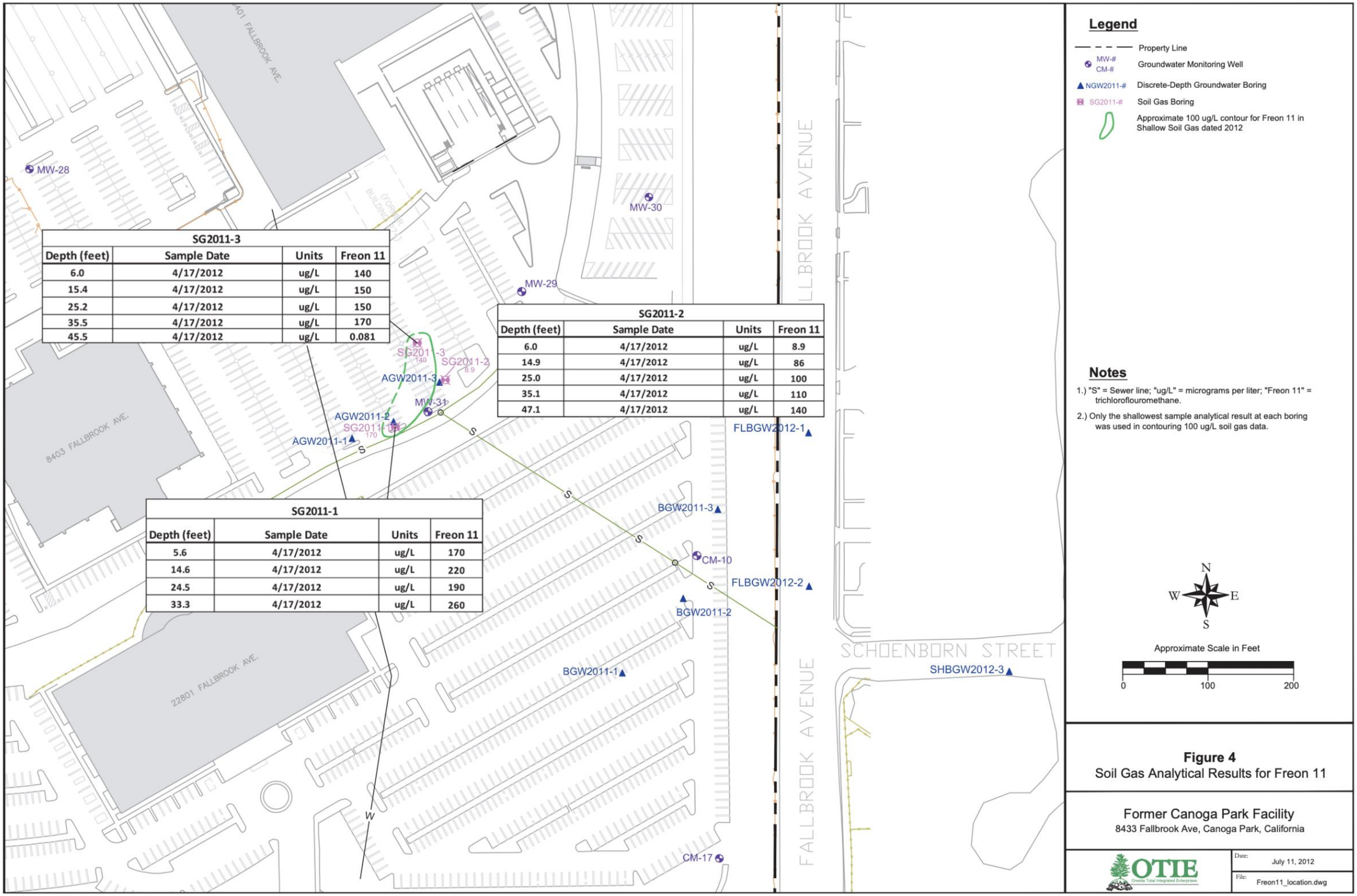

SG2011-3

Depth (feet)	Sample Date	Units	Freon 11
6.0	4/17/2012	ug/L	140
15.4	4/17/2012	ug/L	150
25.2	4/17/2012	ug/L	150
35.5	4/17/2012	ug/L	170
45.5	4/17/2012	ug/L	0.081

SG2011-2

Depth (feet)	Sample Date	Units	Freon 11
6.0	4/17/2012	ug/L	8.9
14.9	4/17/2012	ug/L	86
25.0	4/17/2012	ug/L	100
35.1	4/17/2012	ug/L	110
47.1	4/17/2012	ug/L	140

SG2011-1

Depth (feet)	Sample Date	Units	Freon 11
5.6	4/17/2012	ug/L	170
14.6	4/17/2012	ug/L	220
24.5	4/17/2012	ug/L	190
33.3	4/17/2012	ug/L	260

Figure S16. Sampling Locations at Former Raytheon/Atomics International Nuclear Weapons Site, Canoga Park, CA [72], Figure 3.

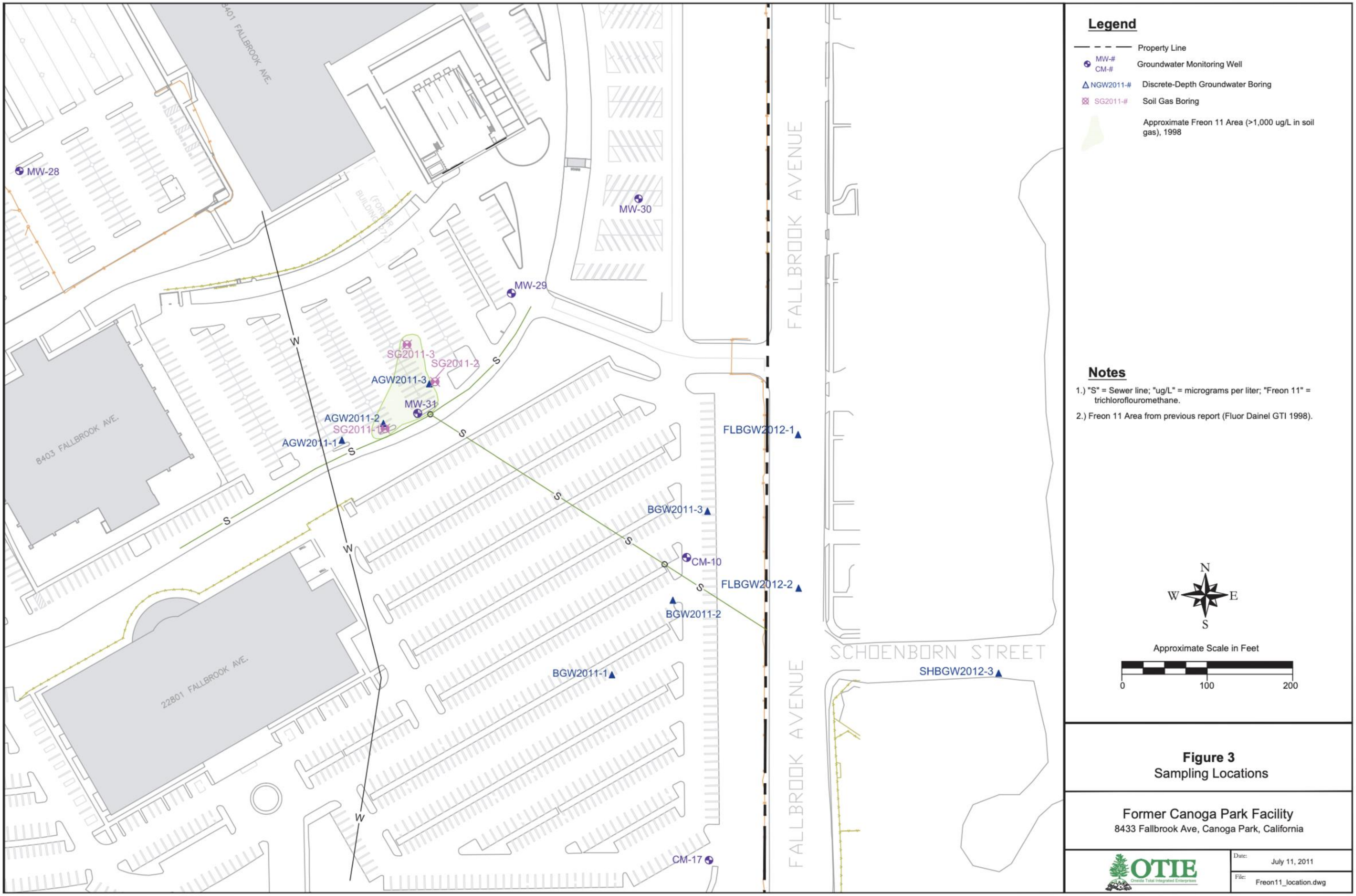

Figure S17. Temporary Soil Vapor Sampling Locations at Former Santa Fe Railyard, Boyle Heights, CA [79], Figure 4.

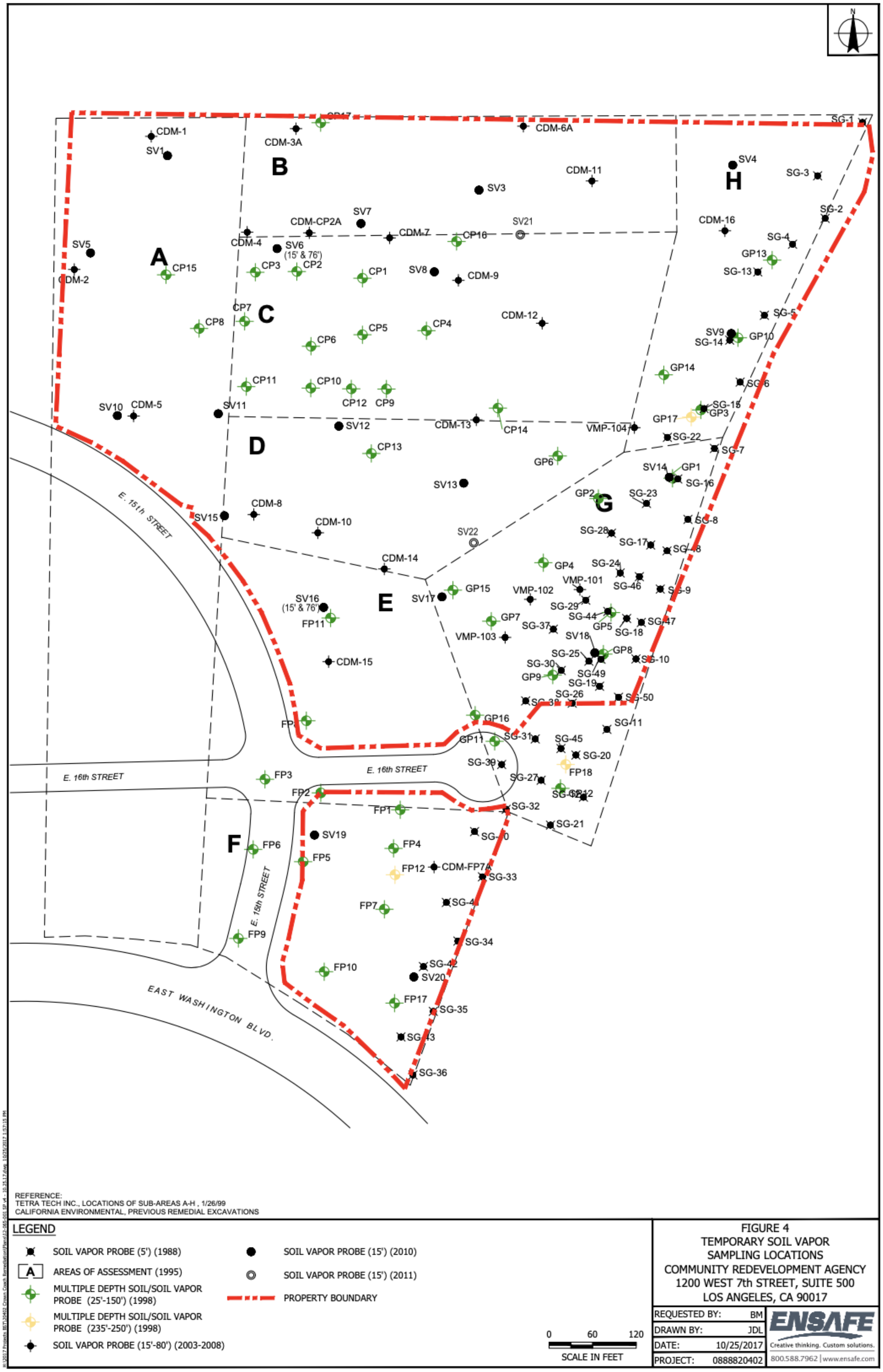

Figure S18. "Hot-Spot" Sampling Locations at Former Naval Ordnance Test Station, Pasadena, CA [58], Figure 7.

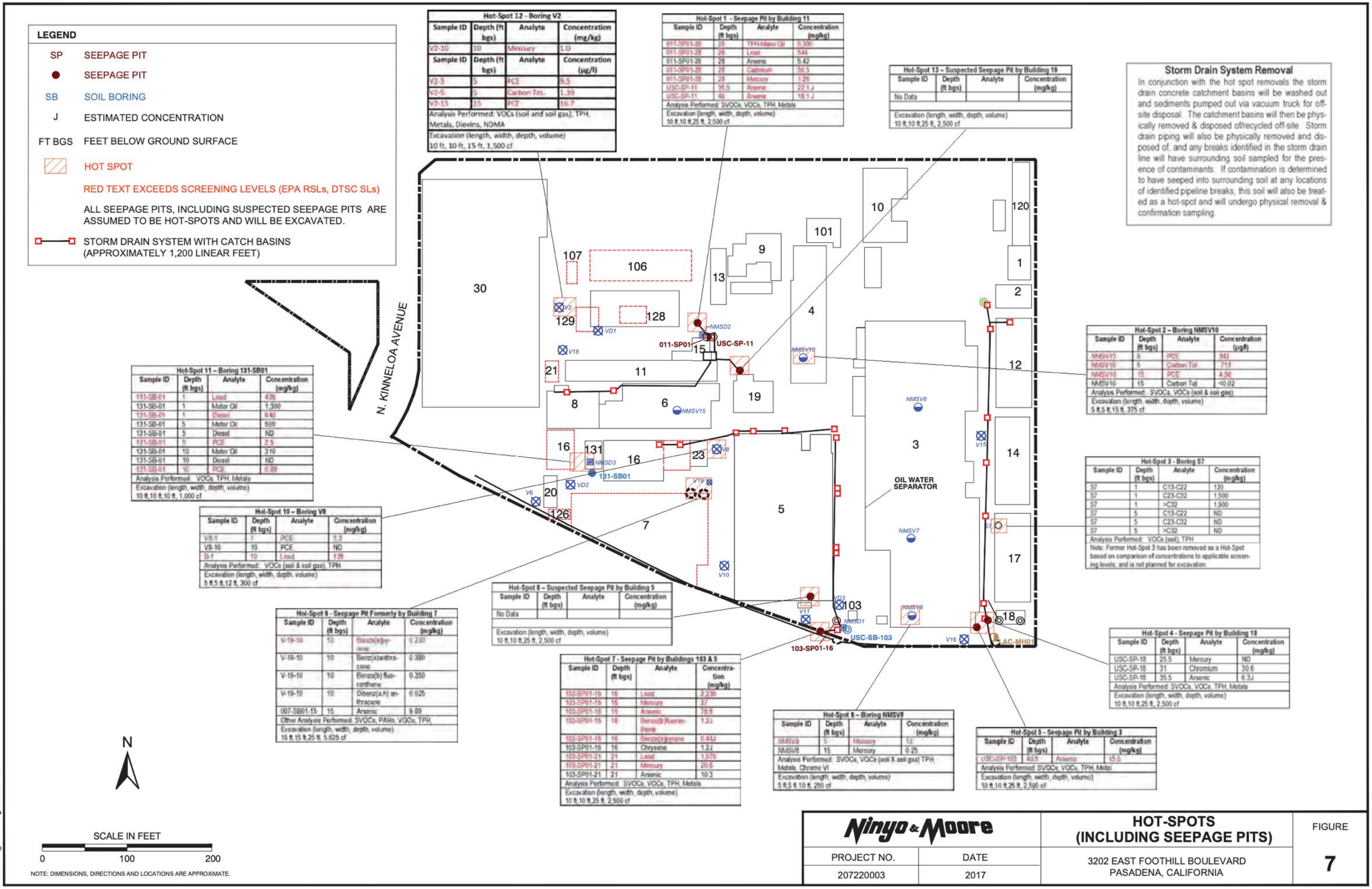

Supplement: Supplementary file 1 [file ijerph-19-07679-s001.zip › 6-2-22-ksf-Supplemental File S1b.pdf]
