# Supplementary material for: Does Hazardous-Waste Testing Follow Technical Guidance, Thus Help Protect Environmental Justice and Health?
Source: Int J Environ Res Public Health. 2022 Jun 23;19(13):7679. doi: 10.3390/ijerph19137679 (PMC9265884; doi:10.3390/ijerph19137679)
Supplement: Supplementary file 1 [file ijerph-19-07679-s001.zip › 6-2-22-ksf-Supplemental File S1c.pdf]

Contents of Supplemental S1c

Tables... 20-33

Table S1…... 21-23
Table S2…... 24-30
Table S3…... 31
Table S4…... 32
Table S5…... 33

TABLE 3 – 2007 REMEDIAL INVESTIGATION ANALYTICAL RESULTS FOR SOIL VAPOR SAMPLES - EPA METHOD 8260B

Sample ID	Date Sample Collected	Depth (feet bgs)	VOCs (µg/l)															
			Carbon Tetrachloride	Chloroform	Dibromochloromethane	Dichlorodifluoromethane	Dichlorotetrafluoroethane	1,1-Dichloroethane	1,1-Dichloroethylene	cis-1,2-Dichloroethylene	Freon 113	Tetrachloroethylene	Toluene	1,1,1-Trichloroethane	Trichloroethylene	Trichlorofluoromethane	Total Xylenes	Other VOCs
V1-5	10/17/2007	5	ND<0.020	ND<0.020	ND<0.020	ND<0.020	ND<0.020	ND<0.020	ND<0.020	ND<0.020	ND<0.020	0.414	ND<0.020	ND<0.020	ND<0.020	ND<0.020	ND<0.020	ND
V1-15	10/17/2007	15	ND<0.020	ND<0.020	ND<0.020	ND<0.020	ND<0.020	ND<0.020	ND<0.020	ND<0.020	ND<0.020	0.170	ND<0.020	ND<0.020	ND<0.020	ND<0.020	ND<0.020	ND
V2-5	10/17/2007	5	1.39	ND<0.020	ND<0.020	0.360	ND<0.020	ND<0.020	ND<0.020	ND<0.020	2.21	9.47	ND<0.020	ND<0.020	ND<0.020	ND<0.020	ND<0.020	ND
V2-15	10/17/2007	15	ND<0.020	ND<0.020	ND<0.020	0.632	ND<0.020	ND<0.020	ND<0.020	ND<0.020	2.87	16.7	ND<0.020	ND<0.020	ND<0.020	0.102	ND<0.020	ND
V3-5	10/17/2007	5	0.462	ND<0.020	ND<0.020	0.212	ND<0.020	ND<0.020	ND<0.020	ND<0.020	0.987	2.29	ND<0.020	ND<0.020	ND<0.020	ND<0.020	0.255	ND
V3-5 DUP	10/17/2007	5	0.456	ND<0.020	ND<0.020	0.186	ND<0.020	ND<0.020	ND<0.020	ND<0.020	0.878	2.34	ND<0.020	ND<0.020	ND<0.020	ND<0.020	ND<0.020	ND
V3-15	10/17/2007	15	0.775	ND<0.020	ND<0.020	0.488	ND<0.020	ND<0.020	ND<0.020	ND<0.020	1.68	4.31	ND<0.020	ND<0.020	ND<0.020	0.029	ND<0.020	ND
V4-5	10/16/2007	5	ND<0.020	ND<0.020	ND<0.020	0.22	ND<0.020	ND<0.020	ND<0.020	ND<0.020	0.304	3.78	ND<0.020	ND<0.020	ND<0.020	ND<0.020	ND<0.020	ND
V4-15	10/16/2007	15	ND<0.020	ND<0.020	ND<0.020	0.265	ND<0.020	ND<0.020	ND<0.020	ND<0.020	5.03	4.79	ND<0.020	ND<0.020	ND<0.020	ND<0.020	ND<0.020	ND
V5-5	10/16/2007	5	ND<0.020	ND<0.020	ND<0.020	0.524	ND<0.020	ND<0.020	ND<0.020	ND<0.020	0.649	28.8	ND<0.020	ND<0.020	0.811	ND<0.020	ND<0.020	ND
V5-15	10/16/2007	15	0.151	ND<0.020	ND<0.020	0.466	ND<0.020	ND<0.020	ND<0.020	ND<0.020	0.878	35.3	ND<0.020	ND<0.020	.496	ND<0.020	ND<0.020	ND
V5-15/QC9-SV**	10/16/2007	15	ND<0.020	ND<0.020	ND<0.020	ND<0.020	4.01	ND<0.020	ND<0.020	ND<0.020	1.80	79.0	ND<0.020	ND<0.020	ND<0.020	1.06	ND<0.020	ND
V6-5	10/16/2007	5	ND<0.020	ND<0.020	ND<0.020	ND<0.020	ND<0.020	ND<0.020	ND<0.020	ND<0.020	ND<0.020	6.75	ND<0.020	ND<0.020	ND<0.020	ND<0.020	ND<0.020	ND
V6-15	10/16/2007	15	ND<0.020	ND<0.020	ND<0.020	0.153	ND<0.020	ND<0.020	ND<0.020	ND<0.020	0.317	20.5	ND<0.020	ND<0.020	ND<0.020	ND<0.020	ND<0.020	ND
V7-5	10/17/2007	5	ND<0.020	ND<0.020	ND<0.020	0.034	ND<0.020	ND<0.020	ND<0.020	ND<0.020	ND<0.020	1.31	0.121	ND<0.020	ND<0.020	ND<0.020	ND<0.020	ND
V7-15	10/17/2007	15	0.464	ND<0.020	ND<0.020	0.338	ND<0.020	ND<0.020	ND<0.020	ND<0.020	0.084	2.75	ND<0.020	ND<0.020	ND<0.020	0.239	ND<0.020	ND
V8-5	10/16/2007	5	0.462	ND<0.020	0.998	ND<0.020	ND<0.020	ND<0.020	ND<0.020	ND<0.020	0.439	6.78	ND<0.020	ND<0.020	ND<0.020	3.12	ND<0.020	ND
V8-15	10/16/2007	15	1.36	ND<0.020	ND<0.020	1.09	ND<0.020	ND<0.020	ND<0.020	ND<0.020	1.31	10.5	ND<0.020	ND<0.020	ND<0.020	ND<0.020	ND<0.020	ND
V9-5	10/18/2007	5	0.265	ND<0.020	ND<0.020	ND<0.020	ND<0.020	ND<0.020	ND<0.020	ND<0.020	0.477	55.6	0.239	0.063	ND<0.020	ND<0.020	0.367	ND
V9-15	10/18/2007	15	0.359	ND<0.020	ND<0.020	0.732	ND<0.020	ND<0.020	ND<0.020	ND<0.020	2.12	137	ND<0.020	ND<0.020	ND<0.020	0.149	ND<0.020	ND
V9-15/QC4-SV**	10/18/2007	15	0.397	ND<0.020	ND<0.020	1.06	ND<0.020	ND<0.020	ND<0.020	ND<0.020	0.341	47.1	ND<0.020	ND<0.020	ND<0.020	ND<0.020	ND<0.020	ND
V10-5	10/17/2007	5	0.694	ND<0.020	ND<0.020	0.230	ND<0.020	ND<0.020	ND<0.020	ND<0.020	0.662	36.3	ND<0.020	ND<0.020	ND<0.020	ND<0.020	ND<0.020	ND
V10-15	10/17/2007	15	1.09	ND<0.020	ND<0.020	0.666	ND<0.020	ND<0.020	ND<0.020	ND<0.020	1.45	34.1	ND<0.020	ND<0.020	ND<0.020	0.077	ND<0.020	ND
V11-5	10/18/2007	5	0.466	ND<0.020	ND<0.020	0.038	ND<0.020	ND<0.020	ND<0.020	ND<0.020	ND<0.020	4.04	ND<0.020	ND<0.020	ND<0.020	ND<0.020	ND<0.020	ND
V11-15	10/18/2007	15	ND<0.020	ND<0.020	ND<0.020	0.259	ND<0.020	ND<0.020	ND<0.020	ND<0.020	0.233	7.98	ND<0.020	ND<0.020	ND<0.020	ND<0.020	ND<0.020	ND
V12-5	10/17/2007	5	0.789	ND<0.020	ND<0.020	0.170	ND<0.020	ND<0.020	ND<0.020	ND<0.020	0.100	2.53	ND<0.020	ND<0.020	ND<0.020	0.212	0.718	ND
V12-15	10/17/2007	15	1.19	ND<0.020	ND<0.020	0.588	ND<0.020	ND<0.020	ND<0.020	ND<0.020	0.499	5.69	ND<0.020	ND<0.020	ND<0.020	0.856	ND<0.020	ND
V13-5	10/17/2007	5	ND<0.020	ND<0.020	ND<0.020	ND<0.020	ND<0.020	ND<0.020	ND<0.020	ND<0.020	ND<0.020	0.288	ND<0.020	ND<0.020	ND<0.020	ND<0.020	ND<0.020	ND
V13-15	10/17/2007	15	ND<0.020	ND<0.020	ND<0.020	0.114	ND<0.020	ND<0.020	ND<0.020	ND<0.020	ND<0.020	0.309	ND<0.020	ND<0.020	ND<0.020	0.059	ND<0.020	ND
V14-5	10/17/2007	5	0.220	ND<0.020	ND<0.020	0.051	ND<0.020	ND<0.020	ND<0.020	ND<0.020	ND<0.020	0.662	ND<0.020	ND<0.020	ND<0.020	0.277	ND<0.020	ND
V14-15	10/17/2007	15	0.438	ND<0.020	ND<0.020	0.360	ND<0.020	ND<0.020	ND<0.020	ND<0.020	ND<0.020	0.860	ND<0.020	ND<0.020	ND<0.020	1.12	ND<0.020	ND
V15-5	10/17/2007	5	0.044	ND<0.020	ND<0.020	ND<0.020	ND<0.020	ND<0.020	ND<0.020	ND<0.020	ND<0.020	0.502	ND<0.020	ND<0.020	ND<0.020	ND<0.020	ND<0.020	ND
V15-15	10/17/2007	15	ND<0.020	ND<0.020	ND<0.020	0.140	ND<0.020	ND<0.020	ND<0.020	ND<0.020	ND<0.020	0.221	ND<0.020	ND<0.020	ND<0.020	0.274	ND<0.020	ND
V16-5	10/18/2007	5	ND<0.020	ND<0.020	ND<0.020	ND<0.020	ND<0.020	ND<0.020	ND<0.020	ND<0.020	ND<0.020	0.366	ND<0.020	ND<0.020	ND<0.020	3.88	ND<0.020	ND
V16-15	10/18/2007	15	ND<0.020	ND<0.020	ND<0.020	0.083	ND<0.020	ND<0.020	ND<0.020	ND<0.020	ND<0.020	0.171	ND<0.020	ND<0.020	ND<0.020	2.71	ND<0.020	ND
V17-5	10/16/2007	5	0.024	ND<0.020	ND<0.020	0.031	ND<0.020	ND<0.020	ND<0.020	ND<0.020	ND<0.020	0.965	ND<0.020	ND<0.020	ND<0.020	ND<0.020	ND<0.020	ND
V17-15	10/16/2007	15	ND<0.020	ND<0.020	ND<0.020	0.199	ND<0.020	ND<0.020	ND<0.020	ND<0.020	0.199	1.61	ND<0.020	ND<0.020	ND<0.020	ND<0.020	ND<0.020	ND
V18-5	10/17/2007	5	0.527	ND<0.020	ND<0.020	0.117	ND<0.020	ND<0.020	ND<0.020	ND<0.020	0.775	8.32	ND<0.020	ND<0.020	ND<0.020	ND<0.020	ND<0.020	ND

TABLE 3 – 2007 REMEDIAL INVESTIGATION ANALYTICAL RESULTS FOR SOIL VAPOR SAMPLES - EPA METHOD 8260B

Sample ID	Date Sample Collected	Depth (feet bgs)	VOCs (µg/l)															
			Carbon Tetrachloride	Chloroform	Dibromochloromethane	Dichlorodifluoromethane	Dichlorotetrafluoroethane	1,1-Dichloroethane	1,1-Dichloroethylene	cis-1,2-Dichloroethylene	Freon 113	Tetrachloroethylene	Toluene	1,1,1-Trichloroethane	Trichloroethylene	Trichlorofluoromethane	Total Xylenes	Other VOCs
V18-15	10/17/2007	15	0.636	ND<0.020	ND<0.020	0.130	ND<0.020	ND<0.020	ND<0.020	ND<0.020	0.863	13.5	ND<0.020	ND<0.020	ND<0.020	ND<0.020	ND<0.020	ND
V19-5	10/17/2007	5	0.338	ND<0.020	ND<0.020	0.234	ND<0.020	ND<0.020	ND<0.020	ND<0.020	0.695	30.2	ND<0.020	0.060	ND<0.020	ND<0.020	ND<0.020	ND
V19-10	10/17/2007	10	0.588	ND<0.020	ND<0.020	0.533	ND<0.020	ND<0.020	ND<0.020	ND<0.020	1.17	39.1	ND<0.020	ND<0.020	ND<0.020	0.160	ND<0.020	ND
NMSV1-5	10/16/2007	5	0.170	ND<0.020	ND<0.020	0.198	ND<0.020	ND<0.020	ND<0.020	ND<0.020	.312	7.56	ND<0.020	ND<0.020	ND<0.020	ND<0.020	ND<0.020	ND
NMSV1-15	10/16/2007	15	ND<0.020	ND<0.020	ND<0.020	0.222	ND<0.020	ND<0.020	ND<0.020	ND<0.020	.459	9.59	ND<0.020	ND<0.020	ND<0.020	ND<0.020	ND<0.020	ND
NMSV2-5	10/16/2007	5	ND<0.020	ND<0.020	ND<0.020	ND<0.020	ND<0.020	ND<0.020	ND<0.020	ND<0.020	0.347	4.78	ND<0.020	ND<0.020	ND<0.020	ND<0.020	ND<0.020	ND
NMSV2-5 Dup	10/16/2007	5	0.267	ND<0.020	ND<0.020	ND<0.020	ND<0.020	ND<0.020	ND<0.020	ND<0.020	3.67	5.17	ND<0.020	ND<0.020	ND<0.020	ND<0.020	ND<0.020	ND
NMSV2-15	10/16/2007	15	ND<0.020	ND<0.020	ND<0.020	0.299	ND<0.020	ND<0.020	ND<0.020	ND<0.020	.596	7.13	ND<0.020	ND<0.020	ND<0.020	ND<0.020	ND<0.020	ND
NMSV2-15 Dup	10/16/2007	15	0.074	ND<0.020	ND<0.020	0.315	ND<0.020	ND<0.020	ND<0.020	ND<0.020	.787	10.2	ND<0.020	ND<0.020	ND<0.020	ND<0.020	ND<0.020	ND
NMSV3-5	10/17/2007	5	0.210	ND<0.020	ND<0.020	0.032	ND<0.020	ND<0.020	ND<0.020	ND<0.020	ND<0.020	0.510	ND<0.020	ND<0.020	ND<0.020	0.097	ND<0.020	ND
NMSV3-15	10/17/2007	15	ND<0.020	ND<0.020	ND<0.020	0.292	ND<0.020	ND<0.020	ND<0.020	ND<0.020	ND<0.020	0.747	ND<0.020	ND<0.020	ND<0.020	0.780	ND<0.020	ND
NMSV4-5	10/18/2007	5	ND<0.020	ND<0.020	ND<0.020	ND<0.020	ND<0.020	ND<0.020	ND<0.020	ND<0.020	ND<0.020	0.897	ND<0.020	ND<0.020	ND<0.020	ND<0.020	ND<0.020	ND
NMSV4-15	10/18/2007	15	ND<0.020	ND<0.020	ND<0.020	0.075	ND<0.020	ND<0.020	ND<0.020	ND<0.020	ND<0.020	1.29	ND<0.020	ND<0.020	ND<0.020	ND<0.020	ND<0.020	ND
NMSV5-5	10/16/2007	5	ND<0.020	ND<0.020	ND<0.020	0.082	ND<0.020	ND<0.020	ND<0.020	ND<0.020	ND<0.020	0.237	ND<0.020	ND<0.020	ND<0.020	ND<0.020	ND<0.020	ND
NMSV5-15	10/16/2007	15	ND<0.020	ND<0.020	ND<0.020	0.090	ND<0.020	ND<0.020	ND<0.020	ND<0.020	ND<0.020	0.281	ND<0.020	ND<0.020	ND<0.020	ND<0.020	ND<0.020	ND
NMSV6-5	10/18/2007	5	1.38	ND<0.020	ND<0.020	0.351	ND<0.020	ND<0.020	ND<0.020	ND<0.020	0.125	3.55	ND<0.020	ND<0.020	ND<0.020	1.55	0.205	ND
NMSV6-15	10/18/2007	15	ND<0.020	0.072	ND<0.020	0.882	ND<0.020	ND<0.020	ND<0.020	ND<0.020	0.595	6.56	ND<0.020	ND<0.020	ND<0.020	2.63	ND<0.020	ND
NMSV7-5	10/18/2007	5	1.82	ND<0.020	ND<0.020	ND<0.020	ND<0.020	ND<0.020	ND<0.020	ND<0.020	0.047	6.63	ND<0.020	ND<0.020	ND<0.020	1.85	ND<0.020	ND
NMSV7-15	10/18/2007	15	ND<0.020	0.03	ND<0.020	0.633	ND<0.020	ND<0.020	ND<0.020	ND<0.020	0.179	6.95	ND<0.020	ND<0.020	ND<0.020	ND<0.020	ND<0.020	ND
NMSV8-5	10/18/2007	5	0.571	ND<0.020	ND<0.020	0.039	ND<0.020	ND<0.020	ND<0.020	ND<0.020	ND<0.020	3.03	ND<0.020	ND<0.020	ND<0.020	1.08	ND<0.020	ND
NMSV8-5 DUP	10/18/2007	5	0.515	ND<0.020	ND<0.020	ND<0.020	ND<0.020	ND<0.020	ND<0.020	ND<0.020	ND<0.020	2.92	ND<0.020	ND<0.020	ND<0.020	0.949	ND<0.020	ND
NMSV8-15	10/18/2007	15	0.583	ND<0.020	ND<0.020	0.199	ND<0.020	ND<0.020	ND<0.020	ND<0.020	ND<0.020	3.26	ND<0.020	ND<0.020	ND<0.020	1.32	ND<0.020	ND
NMSV8-15 DUP	10/18/2007	5	ND<0.020	ND<0.020	ND<0.020	0.176	ND<0.020	ND<0.020	ND<0.020	ND<0.020	ND<0.020	2.88	ND<0.020	ND<0.020	ND<0.020	1.15	ND<0.020	ND
NMSV9-5	10/18/2007	5	0.476	ND<0.020	ND<0.020	0.079	ND<0.020	ND<0.020	ND<0.020	ND<0.020	0.066	1.69	ND<0.020	ND<0.020	ND<0.020	ND<0.020	ND<0.020	ND
NMSV9-15	10/18/2007	15	ND<0.020	ND<0.020	ND<0.020	0.310	ND<0.020	ND<0.020	ND<0.020	ND<0.020	0.364	2.64	ND<0.020	ND<0.020	ND<0.020	0.340	ND<0.020	ND
NMSV10-5	10/18/2007	5	0.713	ND<0.020	ND<0.020	0.153	ND<0.020	ND<0.020	ND<0.020	ND<0.020	0.202	342	ND<0.020	ND<0.020	ND<0.020	0.087	ND<0.020	ND
NMSV10-15	10/18/2007	15	ND<0.020	ND<0.020	ND<0.020	0.435	ND<0.020	ND<0.020	ND<0.020	ND<0.020	0.518	4.58	ND<0.020	ND<0.020	ND<0.020	0.519	ND<0.020	ND
NMSV11-5	10/18/2007	5	0.939	ND<0.020	ND<0.020	0.165	ND<0.020	ND<0.020	ND<0.020	ND<0.020	0.237	8.48	ND<0.020	ND<0.020	ND<0.020	ND<0.020	ND<0.020	ND
NMSV11-15	10/18/2007	15	ND<0.020	ND<0.020	ND<0.020	0.413	ND<0.020	ND<0.020	ND<0.020	ND<0.020	0.489	13.5	ND<0.020	ND<0.020	ND<0.020	0.147	ND<0.020	ND
NMSV12-5	10/18/2007	5	0.569	ND<0.020	ND<0.020	0.078	ND<0.020	ND<0.020	ND<0.020	ND<0.020	ND<0.020	8.89	ND<0.020	ND<0.020	ND<0.020	ND<0.020	ND<0.020	ND
NMSV12-15	10/18/2007	15	ND<0.020	ND<0.020	ND<0.020	0.315	ND<0.020	ND<0.020	ND<0.020	ND<0.020	0.259	14.2	ND<0.020	ND<0.020	ND<0.020	ND<0.020	ND<0.020	ND
NMSV13-5	10/16/2007	5	ND<0.020	ND<0.020	ND<0.020	0.157	ND<0.020	ND<0.020	ND<0.020	ND<0.020	0.567	5.51	ND<0.020	ND<0.020	ND<0.020	ND<0.020	ND<0.020	ND
NMSV13-15	10/16/2007	15	0.218	ND<0.020	ND<0.020	0.122	ND<0.020	ND<0.020	ND<0.020	ND<0.020	0.334	2.83	ND<0.020	ND<0.020	ND<0.020	ND<0.020	ND<0.020	ND
NMSV14-5	10/17/2007	5	0.176	ND<0.020	ND<0.020	0.070	ND<0.020	ND<0.020	ND<0.020	ND<0.020	0.025	4.64	0.244	ND<0.020	ND<0.020	ND<0.020	0.362	ND
NMSV14-15	10/17/2007	15	0.232	ND<0.020	ND<0.020	0.331	ND<0.020	ND<0.020	ND<0.020	ND<0.020	0.486	11.6	ND<0.020	ND<0.020	ND<0.020	0.357	ND<0.020	ND
NMSV15-5	10/16/2007	5	0.334	ND<0.020	ND<0.020	0.256	ND<0.020	ND<0.020	ND<0.020	ND<0.020	0.191	7.78	ND<0.020	ND<0.020	ND<0.020	ND<0.020	ND<0.020	ND
NMSV15-15	10/16/2007	15	ND<0.020	ND<0.020	ND<0.020	0.631	ND<0.020	ND<0.020	ND<0.020	ND<0.020	0.657	14.2	ND<0.020	ND<0.020	0.704	ND<0.020	ND<0.020	ND
VD1-20	10/16/2007	20	ND<0.020	ND<0.020	ND<0.020	0.462	ND<0.020	ND<0.020	ND<0.020	ND<0.020	1.77	20.4	ND<0.020	ND<0.020	ND<0.020	0.066	ND<0.020	ND

TABLE 3 – 2007 REMEDIAL INVESTIGATION ANALYTICAL RESULTS FOR SOIL VAPOR SAMPLES - EPA METHOD 8260B

Sample ID	Date Sample Collected	Depth (feet bgs)	VOCs (µg/l)															
			Carbon Tetrachloride	Chloroform	Dibromochloromethane	Dichlorodifluoromethane	Dichlorotetrafluoroethane	1,1-Dichloroethane	1,1-Dichloroethylene	cis-1,2-Dichloroethylene	Freon 113	Tetrachloroethylene	Toluene	1,1,1-Trichloroethane	Trichloroethylene	Trichlorofluoromethane	Total Xylenes	Other VOCs
VD1-30	10/16/2007	30	ND<0.020	ND<0.020	ND<0.020	0.444	ND<0.020	ND<0.020	ND<0.020	ND<0.020	2.05	8.98	ND<0.020	ND<0.020	0.049	0.079	ND<0.020	ND
VD1-30/QC1-SV**	10/16/2007	30	2.27	ND<0.020	ND<0.020	ND<0.020	3.67	ND<0.020	ND<0.020	ND<0.020	3.89	10.8	0.228	ND<0.020	ND<0.020	14.7	ND<0.020	ND
VD2-20	10/16/2007	20	ND<0.020	ND<0.020	ND<0.020	ND<0.020	ND<0.020	ND<0.020	ND<0.020	ND<0.020	ND<0.020	9.17	ND<0.020	ND<0.020	ND<0.020	ND<0.020	ND<0.020	ND
VD2-30	10/16/2007	30	0.475	ND<0.020	ND<0.020	0.441	ND<0.020	ND<0.020	ND<0.020	ND<0.020	1.07	38.3	ND<0.020	ND<0.020	ND<0.020	0.082	ND<0.020	ND
VD2-30/QC2-SV**	10/16/2007	30	ND<0.020	ND<0.020	ND<0.020	ND<0.020	2.03	ND<0.020	ND<0.020	ND<0.020	1.17	122	ND<0.020	ND<0.020	ND<0.020	ND<0.020	ND<0.020	ND
VD3-20	10/17/2007	20	1.45	ND<0.020	ND<0.020	0.106	ND<0.020	ND<0.020	ND<0.020	ND<0.020	0.086	3.37	ND<0.020	ND<0.020	ND<0.020	ND<0.020	ND<0.020	ND
VD3-30	10/17/2007	30	1.42	ND<0.020	ND<0.020	0.394	ND<0.020	ND<0.020	ND<0.020	ND<0.020	0.374	5.63	ND<0.020	ND<0.020	ND<0.020	0.364	ND<0.020	ND
VD3-30/QC3-SV**	10/17/2007	30	ND<0.020	ND<0.020	ND<0.020	ND<0.020	ND<0.020	ND<0.020	ND<0.020	ND<0.020	ND<0.020	1.33	ND<0.020	ND<0.020	ND<0.020	ND<0.020	ND<0.020	ND
NMSD1-45	10/18/2007	45	0.466	ND<0.020	ND<0.020	ND<0.020	ND<0.020	ND<0.020	ND<0.020	ND<0.020	ND<0.020	4.04	ND<0.020	ND<0.020	ND<0.020	ND<0.020	ND<0.020	ND
NMSD1-70	10/18/2007	70	ND<0.020	0.243	ND<0.020	1.52	ND<0.020	ND<0.020	ND<0.020	ND<0.020	ND<0.020	1.46	ND<0.020	ND<0.020	ND<0.020	1.36	ND<0.020	ND
NMSD1-85	10/18/2007	85	7.53	ND<0.020	ND<0.020	1.09	ND<0.020	ND<0.020	ND<0.020	ND<0.020	1.43	1.59	ND<0.020	ND<0.020	ND<0.020	1.10	ND<0.020	ND
NMSD1-99	10/18/2007	99	ND<0.020	0.372	ND<0.020	ND<0.020	ND<0.020	ND<0.020	ND<0.020	ND<0.020	2.41	0.886	ND<0.020	ND<0.020	ND<0.020	1.39	ND<0.020	ND
NMSD1-99/QC5-SV	10/18/2007	99	5.95	ND<0.020	ND<0.020	9.32	ND<0.020	ND<0.020	ND<0.020	ND<0.020	1.49	0.407	ND<0.020	ND<0.020	ND<0.020	1.06	ND<0.020	ND
NMSD2-63	10/26/2007	63	2.57	ND<0.020	ND<0.020	1.63	ND<0.020	ND<0.020	ND<0.020	ND<0.020	8.68	0.348	ND<0.020	ND<0.020	0.047	0.611	ND<0.020	ND
NMSD2-63 DUP	10/26/2007	63	2.67	ND<0.020	ND<0.020	1.78	ND<0.020	ND<0.020	ND<0.020	ND<0.020	9.29	0.346	ND<0.020	ND<0.020	0.036	ND<0.020	ND<0.020	ND
NMSD2-92	10/26/2007	92	7.20	ND<0.020	ND<0.020	2.50	ND<0.020	ND<0.020	ND<0.020	ND<0.020	14.9	0.454	ND<0.020	ND<0.020	0.091	ND<0.020	ND<0.020	ND
NMSD2-130	10/26/2007	130	12.9	ND<0.020	ND<0.020	2.66	ND<0.020	ND<0.020	0.062	ND<0.020	16.5	1.43	ND<0.020	ND<0.020	0.288	ND<0.020	ND<0.020	ND
NMSD2-150	10/26/2007	150	13.2	ND<0.020	ND<0.020	2.53	ND<0.020	ND<0.020	0.251	ND<0.020	14.8	1.98	ND<0.020	ND<0.020	0.384	ND<0.020	ND<0.020	ND
NMSD2-150/QC6-SV	10/26/2007	150	9.83	ND<0.020	ND<0.020	1.41	ND<0.020	ND<0.020	0.650	ND<0.020	17.6	1.84	ND<0.020	ND<0.020	0.496	ND<0.020	ND<0.020	ND
NMSD3-60	10/26/2007	60	8.39	0.339	ND<0.020	1.56	ND<0.020	0.232	0.728	0.060	6.96	22.3	ND<0.020	ND<0.020	2.39	ND<0.020	ND<0.020	ND
NMSD3-60/QC7-SV	10/26/2007	60	7.18	0.340	ND<0.020	1.11	ND<0.020	0.311	0.552	0.184	9.18	15.6	ND<0.020	ND<0.020	1.52	ND<0.020	ND<0.020	ND
NMSD3-84	10/26/2007	84	24.3	0.724	ND<0.020	2.62	ND<0.020	0.940	1.85	1.23	13.0	22.2	ND<0.020	ND<0.020	6.99	ND<0.020	ND<0.020	ND
NMSD3-113	10/26/2007	113	28.4	1.12	ND<0.020	2.89	ND<0.020	1.56	2.66	2.53	14.9	17.9	ND<0.020	ND<0.020	8.59	ND<0.020	ND<0.020	ND
NMSD3-150	10/26/2007	150	20.6	0.728	ND<0.020	1.63	ND<0.020	0.833	1.7	0.873	8.72	14.6	ND<0.020	ND<0.020	2.92	ND<0.020	ND<0.020	ND
NMSD3-150/QC8-SV	10/26/2007	150	18.5	0.561	ND<0.020	1.06	ND<0.020	0.580	1.95	0.642	12.2	11.8	ND<0.020	ND<0.020	1.83	ND<0.020	ND<0.020	ND
**Maximum Value (ug/l)**			28.4	1.12	.998	9.32	4.01	1.56	1.95	2.53	17.6	342.00	.244	.063	8.59	14.70	.718	--
**Site Screening Values (µg/l)**			0.000067	0.00012	0.00013	0.10	- -	0.0018	0.073	0.0083	31.00	0.00046	0.31	1.00	0.00048	1.30	0.10	--
**Site Screening Values Source**			DTSC	EPA	DTSC	EPA	- -	EPA	DTSC	DTSC	EPA	DTSC	DTSC	DTSC	EPA	DTSC	EPA	--

Notes:

[highlighted] indicates exceedance of the regulatory screening criteria
*Dilutions for these compounds
**Quality Assurance/Quality Control Samples collected in metal 1 liter SUMMA Canisters and analyzed by EPA Method TO-15
-- – Not applicable
bgs – below ground surface
DTSC – Department of Toxic Substances Control
DUP – Duplicate sample
EPA – United States Environmental Protection Agency
ID - identification
µg/l – micrograms per liter
ND<0.020 – Non detect and below listed detection limit
Site Screening Value Source – EPA Residential Regional Screening Levels (RSLs) or DTSC Screening Levels for Residential Ambient Air (HHRA Note 3)
VOCs – Volatile Organic Compounds

Table S2. Summary of Detected Volatile Organic Compounds in Soil Vapor Samples at Former Santa Fe Railyard, Boyle Heights, CA [79], Table 2.

Table 2
Summary of Detected Volatile Organic Compounds in Soil Vapor Samples
Test Method EPA 8260SV (Modified EPA 8260B)
Crown Coach
2425 and 2455 East Washington Boulevard, Los Angeles, California

H&P 8260 SV				Volatile Organic Compounds (µg/L)							2013 Depth Specific Cleanup Goals	
Location	Field Sample ID	Depth (feet bgs)	Date	1,1,1-Trichloroethane	1,1-Dichloroethene	Benzene	Chloroform	cis-1,2-Dichloroethene	Tetrachloroethene	Trichloroethene	Tetrachloroethene	Trichloroethene
				71-55-6	75-35-4	71-43-2	67-66-3	156-59-2	127-18-4	79-01-6	127-18-4	79-01-6
CC-SV-01-15	CC-SV-01-15', P33359cc	15	02/11/2013 12:48:00	ND (<0.20)	ND (<0.20)	ND (<0.04)	ND (<0.04)	ND (<0.20)	**1.5**	ND (<0.04)	71	19
	CC-SV-01		09/29/2014 12:43:00	ND (<0.50)	ND (<0.50)	ND (<0.10)	ND (<0.10)	ND (<0.50)	**2.2**	**0.10**		
	CC-SV-01-15		11/03/2015 12:49:00	ND (<0.50)	ND (<0.50)	ND (<0.10)	ND (<0.10)	ND (<0.50)	**3.2**	ND (<0.10)		
	CC-SV-01-15		08/02/2016 08:29:00	ND (<0.50)	ND (<0.50)	ND (<0.10)	ND (<0.10)	ND (<0.50)	**0.58**	ND (<0.10)		
	CC-SV-01-15		03/09/2017 07:30:00	ND (<0.50)	ND (<0.50)	ND (<0.10)	ND (<0.10)	ND (<0.50)	**1.8**	ND (<0.10)		
CC-SV-02-15	CC-SV-02-15', P33359cc	15	02/13/2013 10:26:00	ND (<0.20)	ND (<0.20)	ND (<0.04)	ND (<0.04)	ND (<0.20)	**7.6**	**0.35**	71	19
	CC-SV-02		09/29/2014 11:00:00	ND (<0.50)	ND (<0.50)	ND (<0.10)	ND (<0.10)	ND (<0.50)	**12**	**0.37**		
	CC-SV-02-15'		10/03/2014 07:37:00	ND (<0.50)	ND (<0.50)	**0.11**	ND (<0.10)	ND (<0.50)	**7.6**	**1.1**		
	CC-SV-02-15		11/03/2015 09:02:00	ND (<0.50)	ND (<0.50)	ND (<0.10)	ND (<0.10)	ND (<0.50)	**5.8**	**1.0**		
	CC-SV-02-15		08/01/2016 07:43:00	ND (<0.50)	ND (<0.50)	ND (<0.10)	ND (<0.10)	ND (<0.50)	**1.5**	ND (<0.10)		
	CC-SV-02-15		03/07/2017 08:40:00	ND (<0.50)	ND (<0.50)	ND (<0.10)	ND (<0.10)	ND (<0.50)	**1.2**	ND (<0.10)		
CC-SV-02-45	CC-SV-02-45', P33741cc	45	02/13/2013 10:46:00	ND (<0.20)	ND (<0.20)	ND (<0.04)	ND (<0.04)	ND (<0.20)	**20**	**2.0**	181	48
	CC-SV-02-45		11/03/2015 09:17:00	ND (<0.50)	ND (<0.50)	ND (<0.10)	ND (<0.10)	ND (<0.50)	**14**	**0.33**		
	CC-SV-02-45 REP		11/03/2015 09:23:00	ND (<0.50)	ND (<0.50)	ND (<0.10)	ND (<0.10)	ND (<0.50)	**9.6**	**0.31**		
	CC-SV-02-45		08/03/2016 09:10:00	ND (<0.50)	ND (<0.50)	ND (<0.10)	ND (<0.10)	ND (<0.50)	**2.6**	ND (<0.10)		
	CC-SV-02-45		03/07/2017 08:48:00	ND (<0.50)	ND (<0.50)	ND (<0.10)	ND (<0.10)	ND (<0.50)	**5.7**	**0.20**		
CC-SV-02-84	CC-SV-02-84', P34117cc	84	02/13/2013 11:10:00	ND (<0.20)	**3.3**	ND (<0.04)	**0.10**	ND (<0.20)	**82**	**17**	324	85
	CC-SV-02-84'		10/03/2014 07:44:00	ND (<0.50)	ND (<0.50)	**0.12**	ND (<0.10)	ND (<0.50)	**13**	**1.2**		
	CC-SV-02-84		11/03/2015 09:33:00	ND (<0.50)	ND (<0.50)	ND (<0.10)	ND (<0.10)	ND (<0.50)	**13**	**0.79**		
	CC-SV-02-84		08/01/2016 07:47:00	ND (<0.50)	ND (<0.50)	ND (<0.10)	ND (<0.10)	ND (<0.50)	**1.8**	**0.10**		
	CC-SV-02-84 rep		08/01/2016 07:47:00	ND (<0.50)	ND (<0.50)	ND (<0.10)	ND (<0.10)	ND (<0.50)	**1.6**	**0.12**		
	CC-SV-02-84		03/07/2017 08:53:00	ND (<0.50)	ND (<0.50)	ND (<0.10)	ND (<0.10)	ND (<0.50)	**4.1**	**0.16**		
CC-SV-03-15	CC-SV-03-15', P33359cc	15	02/12/2013 07:25:00	ND (<0.20)	ND (<0.20)	ND (<0.04)	ND (<0.04)	ND (<0.20)	**1.7**	ND (<0.04)	71	19
	CC-SV-03		09/29/2014 09:05:00	ND (<0.50)	ND (<0.50)	ND (<0.10)	ND (<0.10)	ND (<0.50)	**7.4**	**0.11**		
	CC-SV-03-15		10/13/2014 08:16:00	ND (<0.50)	ND (<0.50)	ND (<0.10)	ND (<0.10)	ND (<0.50)	**4.7**	**0.16**		
	CC-SV-03-15		11/03/2015 11:14:00	ND (<0.50)	ND (<0.50)	ND (<0.10)	ND (<0.10)	ND (<0.50)	**8.9**	**0.24**		

Table S2. Summary of Detected Volatile Organic Compounds in Soil Vapor Samples at Former Santa Fe Railyard, Boyle Heights, CA [79], Table 2.

	CC-SV-03-15		08/02/2016 07:46:00	ND (<0.50)	ND (<0.50)	ND (<0.10)	ND (<0.10)	ND (<0.50)	**6.7**	**0.25**		
	CC-SV-03-15		03/08/2017 12:36:00	ND (<0.50)	ND (<0.50)	**0.10**	ND (<0.10)	ND (<0.50)	**7.9**	**0.25**		
CC-SV-03-40	CC-SV-03-40', P33722cc	40	02/12/2013 07:45:00	ND (<0.20)	ND (<0.20)	ND (<0.04)	ND (<0.04)	ND (<0.20)	**3.1**	ND (<0.04)	162	43
	CC-SV-03-40		11/03/2015 11:25:00	ND (<0.50)	ND (<0.50)	ND (<0.10)	ND (<0.10)	ND (<0.50)	**7.5**	**0.13**		
	CC-SV-03-40		08/02/2016 07:54:00	ND (<0.50)	ND (<0.50)	ND (<0.10)	ND (<0.10)	ND (<0.50)	**4.4**	**0.13**		
	CC-SV-03-40		03/08/2017 12:44:00	ND (<0.50)	ND (<0.50)	ND (<0.10)	ND (<0.10)	ND (<0.50)	**9.8**	**0.29**		
CC-SV-03-57	CC-SV-03-57', P33905c	57	02/12/2013 08:05:00	ND (<0.20)	ND (<0.20)	ND (<0.04)	ND (<0.04)	ND (<0.20)	**1.9**	ND (<0.04)	225	60
	CC-SV-03-57		10/13/2014 08:28:00	ND (<0.50)	ND (<0.50)	ND (<0.10)	ND (<0.10)	ND (<0.50)	**9.7**	**0.22**		
	CC-SV-03-57		11/03/2015 11:46:00	ND (<0.50)	ND (<0.50)	ND (<0.10)	ND (<0.10)	ND (<0.50)	**12**	**0.19**		
	CC-SV-03-57		08/02/2016 08:00:00	ND (<0.50)	ND (<0.50)	ND (<0.10)	ND (<0.10)	ND (<0.50)	**2.1**	ND (<0.10)		
	CC-SV-03-57		03/08/2017 13:01:00	ND (<0.50)	ND (<0.50)	ND (<0.10)	ND (<0.10)	ND (<0.50)	**4.7**	**0.12**		
CC-SV-04R-15	CC-SV-04-15', P33359cc	15	02/13/2013 11:30:00	ND (<0.20)	ND (<0.20)	ND (<0.04)	ND (<0.04)	ND (<0.20)	**4.6**	**0.42**	71	19
	CC-SV-04R-15		11/02/2015 09:53:00	ND (<0.50)	ND (<0.50)	ND (<0.10)	ND (<0.10)	ND (<0.50)	**12**	**0.95**		
	CC-SV-04R-15 REP		11/02/2015 09:54:00	ND (<0.50)	ND (<0.50)	ND (<0.10)	ND (<0.10)	ND (<0.50)	**13**	**1.0**		
	CC-SV-04R-15		08/01/2016 10:02:00	ND (<0.50)	ND (<0.50)	ND (<0.10)	ND (<0.10)	ND (<0.50)	**5.7**	**0.61**		
	CC-SV-04R-15		03/07/2017 10:16:00	ND (<0.50)	ND (<0.50)	ND (<0.10)	ND (<0.10)	ND (<0.50)	**3.1**	**0.28**		
CC-SV-04R-40	CC-SV-04-40', P33722cc	40	02/13/2013 11:50:00	ND (<0.20)	**0.70**	ND (<0.04)	ND (<0.04)	ND (<0.20)	**22**	**2.5**	162	43
	CC-SV-04R-40		11/02/2015 10:05:00	ND (<0.50)	ND (<0.50)	ND (<0.10)	ND (<0.10)	ND (<0.50)	**28**	**2.6**		
	CC-SV-04R-40		08/01/2016 10:10:00	ND (<0.50)	ND (<0.50)	ND (<0.10)	ND (<0.10)	ND (<0.50)	**5.4**	**0.32**		
	CC-SV-04R-40		03/07/2017 10:25:00	ND (<0.50)	ND (<0.50)	ND (<0.10)	ND (<0.10)	ND (<0.50)	**7.3**	**1.2**		
CC-SV-04R-62	CC-SV-04-60', P34007cc	60	02/13/2013 12:10:00	ND (<0.20)	**0.99**	ND (<0.04)	ND (<0.04)	ND (<0.20)	**39**	**4.2**	236	63
	CC-SV-04R-62	62	11/02/2015 10:20:00	ND (<0.50)	ND (<0.50)	ND (<0.10)	ND (<0.10)	ND (<0.50)	**25**	**2.2**	243	65
	CC-SV-04R-62R		11/03/2015 10:06:00	ND (<0.50)	ND (<0.50)	ND (<0.10)	ND (<0.10)	ND (<0.50)	**31**	**2.6**		
	CC-SV-04R-62		08/01/2016 10:14:00	ND (<0.50)	ND (<0.50)	ND (<0.10)	ND (<0.10)	ND (<0.50)	**4.1**	**0.11**		
	CC-SV-04R-62		03/07/2017 10:37:00	ND (<0.50)	ND (<0.50)	ND (<0.10)	ND (<0.10)	ND (<0.50)	**3.3**	**0.17**		
CC-SV-05-15	CC-SV-05-15', P33359cc	15	02/11/2013 10:52:00	ND (<0.20)	ND (<0.20)	ND (<0.04)	ND (<0.04)	ND (<0.20)	**7.4**	**0.76**	71	19
	CC-SV-05		09/29/2014 09:22:00	ND (<0.50)	ND (<0.50)	ND (<0.10)	ND (<0.10)	ND (<0.50)	**15**	**1.1**		
	CC-SV-05-15		10/13/2014 08:57:00	ND (<0.50)	ND (<0.50)	ND (<0.10)	ND (<0.10)	ND (<0.50)	**5.7**	**0.48**		
	CC-SV-05-15		11/02/2015 13:10:00	ND (<0.50)	ND (<0.50)	ND (<0.10)	ND (<0.10)	ND (<0.50)	**15**	**0.96**		
	CC-SV-05-15		08/01/2016 12:54:00	ND (<0.50)	ND (<0.50)	ND (<0.10)	ND (<0.10)	ND (<0.50)	**5.9**	**0.43**		
	CC-SV-05-15		03/07/2017 13:43:00	ND (<0.50)	ND (<0.50)	ND (<0.10)	ND (<0.10)	ND (<0.50)	**3.6**	**0.20**		
CC-SV-05-40	CC-SV-05-40', P33722cc	40	02/11/2013 11:13:00	ND (<0.20)	ND (<0.20)	ND (<0.04)	ND (<0.04)	ND (<0.20)	**13**	**1.3**	162	43
	CC-SV-05-40		11/02/2015 13:22:00	ND (<0.50)	ND (<0.50)	ND (<0.10)	ND (<0.10)	ND (<0.50)	**10**	**0.56**		
	CC-SV-05-40		08/01/2016 12:27:00	ND (<0.50)	ND (<0.50)	ND (<0.10)	ND (<0.10)	ND (<0.50)	**1.8**	ND (<0.10)		
	CC-SV-05-40		03/07/2017 12:58:00	ND (<0.50)	ND (<0.50)	ND (<0.10)	ND (<0.10)	ND (<0.50)	**2.9**	ND (<0.10)		
CC-SV-05-59	CC-SV-05-59', P34003cc	59	02/11/2013 11:34:00	ND (<0.20)	ND (<0.20)	ND (<0.04)	ND (<0.04)	ND (<0.20)	**15**	**0.83**	233	62
	CC-SV-05-59		10/13/2014 09:17:00	ND (<0.50)	**0.89**	ND (<0.10)	ND (<0.10)	**0.80**	**190**	**9.2**		
CC-SV-05DR-57	CC-SV-05DR-57	57	11/02/2015 13:51:00	ND (<0.50)	ND (<0.50)	ND (<0.10)	ND (<0.10)	ND (<0.50)	**25**	**1.2**	224	61

Table S2. Summary of Detected Volatile Organic Compounds in Soil Vapor Samples at Former Santa Fe Railyard, Boyle Heights, CA [79], Table 2.

	CC-SV-05DR-57		08/01/2016 12:37:00	ND (<0.50)	ND (<0.50)	ND (<0.10)	ND (<0.10)	ND (<0.50)	**3.0**	ND (<0.10)		
	CC-SV-05DR-57		03/07/2017 13:20:00	ND (<0.50)	ND (<0.50)	ND (<0.10)	ND (<0.10)	ND (<0.50)	**6.4**	**0.20**		
CC-SV-06-15	CC-SV-06-15', P33359cc	15	02/13/2013 08:20:00	ND (<0.20)	ND (<0.20)	ND (<0.04)	ND (<0.04)	ND (<0.20)	**7.9**	**0.86**	71	19
	CC-SV-06		09/29/2014 11:14:00	ND (<0.50)	ND (<0.50)	ND (<0.10)	ND (<0.10)	ND (<0.50)	**19**	**2.4**		
	CC-SV-06-15'		10/03/2014 07:57:00	ND (<0.50)	ND (<0.50)	**0.18**	ND (<0.10)	ND (<0.50)	**10**	**0.83**		
				**NA**	**NA**	**NA**	**NA**	**NA**	**NA**	**NA**		
CC-SV-06SMR-15	CC-SV-06SMR-15	15	11/02/2015 08:33:00	ND (<0.50)	ND (<0.50)	ND (<0.10)	ND (<0.10)	ND (<0.50)	**19**	**1.2**	71	19
	CC-SV-06SMR-15		08/01/2016 08:40:00	ND (<0.50)	ND (<0.50)	ND (<0.10)	ND (<0.10)	ND (<0.50)	**5.0**	**0.33**		
	CC-SV-06SMR- 15		03/07/2017 08:34:00	ND (<0.50)	ND (<0.50)	ND (<0.10)	ND (<0.10)	ND (<0.50)	**2.7**	**0.17**		
CC-SV-06-SMR-40	CC-SV-06-40', P33722cc	40	02/13/2013 08:41:00	ND (<0.20)	**1.1**	ND (<0.04)	ND (<0.04)	ND (<0.20)	**30**	**4.6**	162	43
	CC-SV-06SMR-40		11/02/2015 08:45:00	ND (<0.50)	ND (<0.50)	ND (<0.10)	ND (<0.10)	ND (<0.50)	**26**	**2.5**		
	CC-SV-06SMR-40		08/01/2016 08:37:00	ND (<0.50)	ND (<0.50)	ND (<0.10)	ND (<0.10)	ND (<0.50)	**6.0**	**0.29**		
	CC-SV-06SMR- 40		03/07/2017 08:08:00	ND (<0.50)	ND (<0.50)	ND (<0.10)	ND (<0.10)	ND (<0.50)	**9.6**	**0.59**		
CC-SV-06-64	CC-SV-06-60' Rep, P34057cc	60	02/13/2013 09:03:00	ND (<0.20)	**2.3**	ND (<0.04)	ND (<0.04)	ND (<0.20)	**60**	**11**	236	63
	CC-SV-06-60', P34007cc		02/13/2013 09:02:00	ND (<0.20)	**1.5**	ND (<0.04)	ND (<0.04)	ND (<0.20)	**38**	**6.4**		
	CC-SV-06-64'	64	10/03/2014 08:02:00	ND (<0.50)	ND (<0.50)	**0.15**	ND (<0.10)	ND (<0.50)	**26**	**4.2**	249	67
	CC-SV-06-64		11/02/2015 08:59:00	ND (<0.50)	ND (<0.50)	ND (<0.10)	ND (<0.10)	ND (<0.50)	**23**	**2.8**		
	CC-SV-06-64R		11/03/2015 08:39:00	ND (<0.50)	ND (<0.50)	ND (<0.10)	ND (<0.10)	ND (<0.50)	**32**	**3.8**		
	CC-SV-06-64		08/01/2016 08:22:00	ND (<0.50)	ND (<0.50)	ND (<0.10)	**0.16**	ND (<0.50)	**5.3**	**0.33**		
	CC-SV-06-64		03/07/2017 07:40:00	ND (<0.50)	ND (<0.50)	ND (<0.10)	**0.10**	ND (<0.50)	**7.9**	**0.70**		
	CC-SV-06-64 REP		03/07/2017 07:41:00	ND (<0.50)	ND (<0.50)	ND (<0.10)	ND (<0.10)	ND (<0.50)	**6.6**	**0.55**		
CC-SV-07-15	CC-SV-07-15', P33359cc	15	02/13/2013 07:19:00	ND (<0.20)	ND (<0.20)	ND (<0.04)	ND (<0.04)	ND (<0.20)	**8.1**	**0.73**	71	19
	CC-SV-07		09/29/2014 10:25:00	ND (<0.50)	ND (<0.50)	ND (<0.10)	ND (<0.10)	ND (<0.50)	**14**	**1.3**		
	CC-SV-07-15		11/03/2015 07:46:00	ND (<0.50)	ND (<0.50)	ND (<0.10)	ND (<0.10)	ND (<0.50)	**6.7**	**0.42**		
	CC-SV-07-15		08/01/2016 09:06:00	ND (<0.50)	ND (<0.50)	ND (<0.10)	ND (<0.10)	ND (<0.50)	**2.9**	**0.36**		
	CC-SV-07-15		03/07/2017 09:22:00	ND (<0.50)	ND (<0.50)	ND (<0.10)	ND (<0.10)	ND (<0.50)	**2.4**	**0.12**		
CC-SV-07-40	CC-SV-07-45', P33722cc	40	02/13/2013 07:39:00	ND (<0.20)	**0.84**	ND (<0.04)	ND (<0.04)	ND (<0.20)	**32**	**3.9**	162	43
	CC-SV-07-B		10/13/2014 10:53:00	ND (<0.50)	ND (<0.50)	ND (<0.10)	ND (<0.10)	ND (<0.50)	**28**	**2.9**		
	CC-SV-07-40		11/03/2015 07:59:00	ND (<0.50)	ND (<0.50)	ND (<0.10)	ND (<0.10)	ND (<0.50)	**24**	**1.9**		
	CC-SV-07-40		08/01/2016 09:18:00	ND (<0.50)	ND (<0.50)	ND (<0.10)	ND (<0.10)	ND (<0.50)	**2.2**	**0.17**		
	CC-SV-07-40		03/07/2017 09:35:00	ND (<0.50)	ND (<0.50)	ND (<0.10)	ND (<0.10)	ND (<0.50)	**8.8**	**0.52**		
CC-SV-07-60	CC-SV-07-65.5', P34022cc	60	02/13/2013 08:00:00	ND (<0.20)	**1.3**	ND (<0.04)	ND (<0.04)	ND (<0.20)	**46**	**5.5**	236	63
	CC-SV-07-A		10/13/2014 10:42:00	ND (<0.50)	ND (<0.50)	ND (<0.10)	ND (<0.10)	ND (<0.50)	**3.7**	**0.30**		
	CC-SV-07-60		11/03/2015 08:12:00	ND (<0.50)	ND (<0.50)	ND (<0.10)	ND (<0.10)	ND (<0.50)	**30**	**2.4**		
	CC-SV-07-60		08/01/2016 09:20:00	ND (<0.50)	ND (<0.50)	ND (<0.10)	ND (<0.10)	ND (<0.50)	**7.3**	**0.64**		
	CC-SV-07-60		03/07/2017 09:48:00	ND (<0.50)	ND (<0.50)	ND (<0.10)	ND (<0.10)	ND (<0.50)	**11**	**0.93**		
CC-SV-08-15	CC-SV-08-15', P33359cc	15	02/11/2013 10:27:00	ND (<0.20)	ND (<0.20)	ND (<0.04)	ND (<0.04)	ND (<0.20)	**6.1**	**0.45**	71	19
	CC-SV-08		09/29/2014 12:16:00	ND (<0.50)	**1.5**	ND (<0.10)	ND (<0.10)	ND (<0.50)	**50**	**5.7**		

Table S2. Summary of Detected Volatile Organic Compounds in Soil Vapor Samples at Former Santa Fe Railyard, Boyle Heights, CA [79], Table 2.

	CC-SV-08-15		10/13/2014 09:43:00	ND (<0.50)	ND (<0.50)	ND (<0.10)	ND (<0.10)	ND (<0.50)	**9.6**	**0.93**		
	CC-SV-08-15		11/02/2015 10:46:00	ND (<0.50)	**0.75**	ND (<0.10)	ND (<0.10)	ND (<0.50)	**36**	**3.2**		
	CC-SV-08-15		08/01/2016 11:51:00	ND (<0.50)	ND (<0.50)	ND (<0.10)	ND (<0.10)	ND (<0.50)	**6.2**	**0.56**		
	CC-SV-08-15		03/07/2017 12:09:00	ND (<0.50)	ND (<0.50)	ND (<0.10)	ND (<0.10)	ND (<0.50)	**4.4**	**0.48**		
CC-SV-08-40	CC-SV-08-40', 10PV, P112407cc	40	02/11/2013 08:01:00	ND (<0.20)	**3.9**	ND (<0.04)	ND (<0.04)	ND (<0.20)	**70**	**7.8**	162	43
	CC-SV-08-40', 1PV, P11241cc		02/11/2013 07:34:00	ND (<0.20)	**3.7**	ND (<0.04)	ND (<0.04)	ND (<0.20)	**69**	**7.8**		
	CC-SV-08-40', 3PV, P33722cc		02/11/2013 07:38:00	ND (<0.20)	**4.0**	ND (<0.04)	ND (<0.04)	ND (<0.20)	**77**	**8.4**		
	CC-SV-08-40		11/02/2015 11:00:00	ND (<0.50)	**2.8**	ND (<0.10)	ND (<0.10)	ND (<0.50)	**91**	**10**		
	CC-SV-08-40		08/01/2016 12:00:00	ND (<1.0)	ND (<1.0)	ND (<0.20)	ND (<0.20)	ND (<1.0)	**3.5**	ND (<0.20)		
	CC-SV-08-40		03/07/2017 12:19:00	ND (<0.50)	**0.53**	ND (<0.10)	ND (<0.10)	ND (<0.50)	**6.0**	**0.51**		
CC-SV-08-59.5	CC-SV-08-59.5' Rep, P34055cc	59.5	02/11/2013 10:48:00	ND (<0.20)	**8.4**	ND (<0.04)	ND (<0.04)	ND (<0.20)	**320**	**17**	236	63
	CC-SV-08-59.5', P34005cc		02/11/2013 10:47:00	ND (<0.20)	**7.0**	ND (<0.04)	ND (<0.04)	ND (<0.20)	**280**	**14**		
	CC-SV-08-59.5		10/13/2014 09:57:00	ND (<0.50)	**5.5**	ND (<0.10)	ND (<0.10)	**0.59**	**210**	**19**		
	CC-SV-08-59.5-R		11/02/2015 14:23:00	ND (<0.50)	**5.8**	ND (<0.10)	ND (<0.10)	**0.75**	**250**	**21**		
	CC-SV-08-59.5-RR		11/03/2015 10:37:00	ND (<0.50)	**6.9**	**0.10**	ND (<0.10)	**0.80**	**250**	**25**		
	CC-SV-08-59.5		08/01/2016 12:02:00	ND (<2.5)	ND (<2.5)	ND (<0.50)	ND (<0.50)	ND (<2.5)	**56**	**8.6**		
	CC-SV-08-59.5		03/07/2017 12:29:00	ND (<0.50)	**1.0**	ND (<0.10)	ND (<0.10)	ND (<0.50)	**42**	**6.6**		
CC-SV-09-15	CC-SV-09-15', P33359cc	15	02/12/2013 09:45:00	ND (<0.20)	ND (<0.20)	ND (<0.04)	ND (<0.04)	ND (<0.20)	**8.8**	**1.2**	71	19
	CC-SV-09		09/29/2014 11:58:00	ND (<0.50)	ND (<0.50)	ND (<0.10)	ND (<0.10)	ND (<0.50)	**19**	**3.1**		
	CC-SV-09-15		10/13/2014 11:18:00	ND (<0.50)	ND (<0.50)	ND (<0.10)	ND (<0.10)	ND (<0.50)	**8.9**	**1.6**		
	CC-SV-09-15		11/05/2015 08:49:00	ND (<0.50)	ND (<0.50)	ND (<0.10)	ND (<0.10)	ND (<0.50)	**22**	**3.5**		
	CC-SV-9-15		08/03/2016 07:59:00	ND (<0.50)	ND (<0.50)	ND (<0.10)	ND (<0.10)	ND (<0.50)	**0.42**	ND (<0.10)		
	CC-SV-09-15		03/09/2017 09:04:00	ND (<0.50)	ND (<0.50)	ND (<0.10)	ND (<0.10)	ND (<0.50)	**20**	**3.6**		
CC-SV-09-45	CC-SV-09-45', P33747cc	45	02/12/2013 10:04:00	ND (<0.20)	**0.57**	ND (<0.04)	ND (<0.04)	ND (<0.20)	**22**	**2.7**	181	48
	CC-SV-09-45		11/05/2015 09:00:00	ND (<0.50)	ND (<0.50)	ND (<0.10)	ND (<0.10)	ND (<0.50)	**30**	**4.3**		
	CC-SV-9-45		08/03/2016 08:22:00	ND (<0.50)	ND (<0.50)	ND (<0.10)	ND (<0.10)	ND (<0.50)	**8.7**	**0.90**		
	CC-SV-09-45		03/09/2017 09:25:00	ND (<0.50)	ND (<0.50)	ND (<0.10)	ND (<0.10)	ND (<0.50)	**15**	**1.9**		
	CC-SV-09-45 REP		03/09/2017 09:26:00	ND (<0.50)	ND (<0.50)	ND (<0.10)	ND (<0.10)	ND (<0.50)	**15**	**1.8**		
CC-SV-09-65.5	CC-SV-09-65.5', P34022cc	65.5	02/12/2013 10:04:00	ND (<0.20)	**0.86**	ND (<0.04)	ND (<0.04)	ND (<0.20)	**31**	**3.6**	254	68
	CC-SV-09-65.5		10/13/2014 11:29:00	ND (<0.50)	ND (<0.50)	ND (<0.10)	ND (<0.10)	ND (<0.50)	**24**	**3.7**		
	CC-SV-09-65.5		11/04/2015 14:27:00	ND (<0.50)	ND (<0.50)	ND (<0.10)	ND (<0.10)	ND (<0.50)	**28**	**4.1**		
	CC-SV-9-65.5		08/03/2016 07:56:00	ND (<0.50)	ND (<0.50)	ND (<0.10)	**0.17**	ND (<0.50)	**6.9**	**0.79**		
	CC-SV-09-65.5		03/09/2017 09:43:00	ND (<0.50)	ND (<0.50)	ND (<0.10)	**0.11**	ND (<0.50)	**13**	**1.6**		
CC-SV-10-15	CC-SV-10-15', P33359c	15	02/12/2013 08:53:00	ND (<0.20)	ND (<0.20)	ND (<0.04)	ND (<0.04)	ND (<0.20)	**0.95**	ND (<0.04)	71	19
CC-SV-10R-15	CC-SV-10R-15		11/03/2015 13:15:00	ND (<0.50)	ND (<0.50)	ND (<0.10)	ND (<0.10)	ND (<0.50)	**13**	**1.3**		
	CC-SV-10R-15		08/01/2016 13:19:00	ND (<0.50)	ND (<0.50)	ND (<0.10)	ND (<0.10)	ND (<0.50)	**2.8**	**0.16**		
	CC-SV-10R-15		03/07/2017 13:45:00	ND (<0.50)	ND (<0.50)	ND (<0.10)	ND (<0.10)	ND (<0.50)	**3.0**	**0.30**		

Table S2. Summary of Detected Volatile Organic Compounds in Soil Vapor Samples at Former Santa Fe Railyard, Boyle Heights, CA [79], Table 2.

CC-SV-10-40	CC-SV-10-40', P33722cc	40	02/12/2013 09:15:00	ND (<0.20)	ND (<0.20)	ND (<0.04)	ND (<0.04)	ND (<0.20)	**6.6**	**0.35**	162	43
CC-SV-10R-40	CC-SV-10R-40		11/03/2015 13:26:00	ND (<0.50)	**1.2**	ND (<0.10)	ND (<0.10)	ND (<0.50)	**63**	**6.4**		
	CC-SV-10R-40		08/01/2016 13:22:00	ND (<0.50)	**0.88**	ND (<0.10)	ND (<0.10)	ND (<0.50)	**53**	**7.9**		
	CC-SV-10R-40		03/08/2017 11:39:00	ND (<0.50)	**1.3**	ND (<0.10)	ND (<0.10)	ND (<0.50)	**57**	**11**		
	CC-SV-10R-40 REP		03/08/2017 11:42:00	ND (<0.50)	**1.0**	ND (<0.10)	ND (<0.10)	ND (<0.50)	**45**	**8.2**		
CC-SV-10-60	CC-SV-10-60' Rep, P34070cc	60	02/12/2013 09:36:00	ND (<0.20)	**1.3**	ND (<0.04)	ND (<0.04)	ND (<0.20)	**38**	**4.8**	236	63
	CC-SV-10-60', P34020cc		02/12/2013 09:35:00	ND (<0.20)	**0.83**	ND (<0.04)	ND (<0.04)	ND (<0.20)	**25**	**3.4**		
CC-SV-10R-62	CC-SV-10R-62	62	11/03/2015 13:45:00	ND (<0.50)	ND (<0.50)	ND (<0.10)	ND (<0.10)	ND (<0.50)	**32**	**2.4**	244	65
	CC-SV-10R-62		08/02/2016 07:20:00	ND (<0.50)	ND (<0.50)	ND (<0.10)	ND (<0.10)	ND (<0.50)	**8.8**	**1.7**		
	CC-SV-10R-62		03/08/2017 12:07:00	ND (<0.50)	ND (<0.50)	**0.10**	ND (<0.10)	ND (<0.50)	**12**	**1.4**		
CC-SV-11-15	CC-SV-11-15', P33359cc	15	02/14/2013 07:15:00	ND (<0.20)	ND (<0.20)	ND (<0.04)	ND (<0.04)	ND (<0.20)	**2.0**	**0.10**	71	19
	CC-SV-11-15'		10/03/2014 08:18:00	ND (<0.50)	ND (<0.50)	**0.20**	ND (<0.10)	ND (<0.50)	**7.1**	**0.49**		
CC-SV-12-15	CC-SV-12-15', P33359cc	15	02/13/2013 12:26:00	ND (<0.20)	**2.3**	ND (<0.04)	ND (<0.04)	ND (<0.20)	**41**	**13**	71	19
	CC-SV-12		09/29/2014 11:34:00	ND (<0.50)	**0.86**	ND (<0.10)	ND (<0.10)	ND (<0.50)	**29**	**6.8**		
	CC-SV-12-15'		10/03/2014 08:29:00	ND (<0.50)	ND (<0.50)	**0.12**	ND (<0.10)	ND (<0.50)	**11**	**2.0**		
	CC-SV-12-15		11/05/2015 07:26:00	ND (<0.50)	ND (<0.50)	ND (<0.10)	ND (<0.10)	ND (<0.50)	**21**	**2.8**		
	CC-SV-12-15 REP		11/05/2015 07:27:00	ND (<0.50)	ND (<0.50)	ND (<0.10)	ND (<0.10)	ND (<0.50)	**23**	**3.1**		
	CC-SV-12-15		08/02/2016 13:49:00	ND (<0.50)	ND (<0.50)	ND (<0.10)	ND (<0.10)	ND (<0.50)	**0.98**	**0.20**		
	CC-SV-12-15		03/09/2017 10:05:00	ND (<0.50)	ND (<0.50)	ND (<0.10)	ND (<0.10)	ND (<0.50)	**3.2**	**0.43**		
CC-SV-12-45	CC-SV-12-45', P33747cc	45	02/13/2013 12:46:00	ND (<0.20)	**4.0**	ND (<0.04)	ND (<0.04)	ND (<0.20)	**66**	**26**	181	48
	CC-SV-12-45		11/05/2015 07:37:00	ND (<0.50)	**1.8**	ND (<0.10)	ND (<0.10)	ND (<0.50)	**57**	**13**		
	CC-SV-12-45		08/02/2016 13:51:00	ND (<0.50)	ND (<0.50)	ND (<0.10)	ND (<0.10)	ND (<0.50)	**10**	**1.2**		
	CC-SV-12-45		03/09/2017 10:19:00	ND (<0.50)	ND (<0.50)	ND (<0.10)	ND (<0.10)	ND (<0.50)	**13**	**2.1**		
	CC-SV-12-45		03/09/2017 10:19:00	ND (<0.50)	ND (<0.50)	ND (<0.10)	ND (<0.10)	ND (<0.50)	**13**	**2.1**		
CC-SV-12-60	CC-SV-12-60, P34007cc	60	02/13/2013 13:05:00	ND (<0.20)	**4.2**	ND (<0.04)	ND (<0.04)	ND (<0.20)	**71**	**26**	236	63
	CC-SV-12-66'		10/03/2014 08:35:00	ND (<0.50)	**1.1**	**0.13**	ND (<0.10)	ND (<0.50)	**29**	**8.2**		
	CC-SV-12-66		11/05/2015 08:00:00	ND (<0.50)	**1.7**	ND (<0.10)	ND (<0.10)	ND (<0.50)	**48**	**12**		
	CC-SV-12-66		08/03/2016 07:35:00	ND (<0.50)	ND (<0.50)	ND (<0.10)	ND (<0.10)	ND (<0.50)	**1.1**	**0.21**		
	CC-SV-12-66 rep		08/03/2016 07:35:00	ND (<0.50)	ND (<0.50)	ND (<0.10)	ND (<0.10)	ND (<0.50)	**0.95**	**0.18**		
				**NA**	**NA**	**NA**	**NA**	**NA**	**NA**	**NA**		
CC-SV-13-15	CC-SV-13-15', P33359cc	15	02/14/2013 07:34:00	ND (<0.20)	ND (<0.20)	ND (<0.04)	ND (<0.04)	ND (<0.20)	**32**	**7.5**	71	19
CC-SV-13-C	CC-SV-13-C, P24960cc		11/05/2014 07:37:00	**1.0**	ND (<0.50)	ND (<0.10)	ND (<0.10)	ND (<0.50)	**75**	**25**		
	CC-SV-13-C		01/23/2015 10:03:00	**0.65**	ND (<0.50)	ND (<0.10)	ND (<0.10)	ND (<0.50)	**59**	**15**		
	CC-SV-13-C		11/04/2015 11:48:00	**1.1**	ND (<0.50)	ND (<0.10)	ND (<0.10)	ND (<0.50)	**110**	**31**		
	CC-SV-13-C		08/02/2016 10:07:00	ND (<1.0)	ND (<1.0)	ND (<0.20)	ND (<0.20)	ND (<1.0)	**62**	**14**		
	CC-SV-13-C rep		08/02/2016 10:07:00	ND (<1.0)	ND (<1.0)	ND (<0.20)	ND (<0.20)	ND (<1.0)	**56**	**13**		
	CC-SV-13-C		03/08/2017 09:15:00	ND (<0.50)	ND (<0.50)	ND (<0.10)	ND (<0.10)	ND (<0.50)	**36**	**8.2**		

Table S2. Summary of Detected Volatile Organic Compounds in Soil Vapor Samples at Former Santa Fe Railyard, Boyle Heights, CA [79], Table 2.

CC-SV-13-45	CC-SV-13-45', P33747cc	45	02/14/2013 07:55:00	ND (<0.20)	ND (<0.20)	ND (<0.04)	ND (<0.04)	ND (<0.20)	**17**	**3.4**	181	48
CC-SV-13-A	CC-SV-13-A, P24960cc		11/05/2014 07:12:00	**0.59**	ND (<0.50)	ND (<0.10)	ND (<0.10)	ND (<0.50)	**56**	**18**		
	CC-SV-13-A		01/23/2015 09:37:00	ND (<0.50)	ND (<0.50)	ND (<0.10)	ND (<0.10)	ND (<0.50)	**61**	**15**		
	CC-SV-13-A		11/04/2015 11:11:00	ND (<0.50)	ND (<0.50)	ND (<0.10)	ND (<0.10)	ND (<0.50)	**65**	**13**		
	CC-SV-13-A		08/02/2016 10:30:00	ND (<0.50)	ND (<0.50)	ND (<0.10)	ND (<0.10)	ND (<0.50)	**24**	**11**		
	CC-SV-13-A		03/08/2017 08:57:00	ND (<0.50)	ND (<0.50)	ND (<0.10)	ND (<0.10)	ND (<0.50)	**25**	**4.4**		
CC-SV-13-75	CC-SV-13-75' Rep, P34122cc	75	02/14/2013 08:17:00	ND (<0.20)	ND (<0.20)	ND (<0.04)	**0.16**	ND (<0.20)	**17**	**3.3**	293	77
	CC-SV-13-75', P34072cc		02/14/2013 08:16:00	ND (<0.20)	ND (<0.20)	ND (<0.04)	**0.10**	ND (<0.20)	**12**	**2.2**		
CC-SV-13-B	CC-SV-13-B, P24960cc		11/05/2014 07:24:00	ND (<0.50)	**0.60**	ND (<0.10)	ND (<0.10)	ND (<0.50)	**37**	**10**		
	CC-SV-13-B		01/23/2015 09:52:00	ND (<0.50)	**0.75**	ND (<0.10)	ND (<0.10)	ND (<0.50)	**46**	**9.5**		
	CC-SV-13-B		11/04/2015 11:30:00	ND (<0.50)	ND (<0.50)	ND (<0.10)	ND (<0.10)	ND (<0.50)	**65**	**11**		
	CC-SV-13-B		08/02/2016 10:33:00	ND (<0.50)	ND (<0.50)	ND (<0.10)	ND (<0.10)	ND (<0.50)	**22**	**2.4**		
				**NA**	**NA**	**NA**	**NA**	**NA**	**NA**	**NA**		
CC-SV-14-15	CC-SV-14-15	15	11/03/2015 14:18:00	ND (<0.50)	ND (<0.50)	ND (<0.10)	ND (<0.10)	ND (<0.50)	**22**	**1.7**	71	19
	CC-SV-14-15		08/03/2016 08:35:00	ND (<0.50)	ND (<0.50)	ND (<0.10)	ND (<0.10)	ND (<0.50)	**2.2**	**0.21**		
	CC-SV-14-15		03/09/2017 08:02:00	ND (<0.50)	ND (<0.50)	ND (<0.10)	ND (<0.10)	ND (<0.50)	**2.5**	**0.20**		
CC-SV-14-39	CC-SV-14-39	39	11/04/2015 13:04:00	ND (<0.50)	ND (<0.50)	ND (<0.10)	ND (<0.10)	ND (<0.50)	**52**	**3.1**	159	42
	CC-SV-14-39 REP		11/04/2015 13:05:00	ND (<0.50)	ND (<0.50)	ND (<0.10)	ND (<0.10)	ND (<0.50)	**57**	**3.2**		
	CC-SV-14-39		08/03/2016 08:46:00	ND (<0.50)	ND (<0.50)	ND (<0.10)	ND (<0.10)	ND (<0.50)	**13**	**0.56**		
	CC-SV-14-39		03/09/2017 08:15:00	ND (<0.50)	ND (<0.50)	ND (<0.10)	ND (<0.10)	ND (<0.50)	**7.9**	**0.42**		
CC-SV-14-55	CC-SV-14-55	55	11/04/2015 14:45:00	ND (<0.50)	ND (<0.50)	ND (<0.10)	ND (<0.10)	ND (<0.50)	**31**	**2.3**	218	58
	CC-SV-14-55		08/03/2016 08:49:00	ND (<0.50)	ND (<0.50)	ND (<0.10)	ND (<0.10)	ND (<0.50)	**15**	**0.77**		
	CC-SV-14-55		03/09/2017 08:34:00	ND (<0.50)	ND (<0.50)	ND (<0.10)	ND (<0.10)	ND (<0.50)	**8.3**	**0.38**		
CC-SV-15-15	CC-SV-15-15', P33359cc	15	02/14/2013 07:32:00	ND (<0.20)	ND (<0.20)	ND (<0.04)	ND (<0.04)	ND (<0.20)	**6.6**	**0.79**	71	19
	CC-SV-15-15		01/23/2015 09:15:00	ND (<0.50)	ND (<0.50)	ND (<0.10)	ND (<0.10)	ND (<0.50)	**25**	**3.9**		
	CC-SV-15-15		11/04/2015 10:16:00	ND (<0.50)	ND (<0.50)	ND (<0.10)	ND (<0.10)	ND (<0.50)	**30**	**4.2**		
	CC-SV-15-15		08/02/2016 09:16:00	ND (<0.50)	ND (<0.50)	ND (<0.10)	ND (<0.10)	ND (<0.50)	**8.7**	**1.6**		
	CC-SV-15-15		03/08/2017 08:31:00	ND (<0.50)	ND (<0.50)	ND (<0.10)	ND (<0.10)	ND (<0.50)	**7.4**	**0.79**		
CC-SV-16-15	CC-SV-16-15', P33359cc	15	02/14/2013 08:33:00	ND (<0.20)	ND (<0.20)	ND (<0.04)	ND (<0.04)	ND (<0.20)	**4.4**	**0.48**	71	19
CC-SV-16-C	CC-SV-16-C, P24990cc		11/05/2014 08:59:00	ND (<0.50)	ND (<0.50)	ND (<0.10)	ND (<0.10)	ND (<0.50)	**9.5**	**1.4**		
	CC-SV-16-C		01/23/2015 07:51:00	ND (<0.50)	ND (<0.50)	ND (<0.10)	ND (<0.10)	ND (<0.50)	**7.6**	**0.81**		
	CC-SV-16-C		11/04/2015 08:39:00	ND (<0.50)	ND (<0.50)	ND (<0.10)	ND (<0.10)	ND (<0.50)	**15**	**1.4**		
	CC-SV-16-C		08/02/2016 13:18:00	ND (<0.50)	ND (<0.50)	ND (<0.10)	ND (<0.10)	ND (<0.50)	**1.3**	**0.17**		
	CC-SV-16-C		03/08/2017 10:57:00	ND (<0.50)	ND (<0.50)	ND (<0.10)	ND (<0.10)	ND (<0.50)	**1.5**	**0.21**		
CC-SV-16-45	CC-SV-16-45', P33747cc	45	02/14/2013 08:34:00	ND (<0.20)	ND (<0.20)	ND (<0.04)	ND (<0.04)	ND (<0.20)	**11**	**1.5**	181	48
CC-SV-16-B	CC-SV-16-B, P24990cc		11/05/2014 08:49:00	ND (<0.50)	ND (<0.50)	ND (<0.10)	ND (<0.10)	ND (<0.50)	**40**	**6.1**		
	CC-SV-16-B		01/23/2015 07:41:00	ND (<0.50)	ND (<0.50)	ND (<0.10)	ND (<0.10)	ND (<0.50)	**25**	**2.3**		

Table S2. Summary of Detected Volatile Organic Compounds in Soil Vapor Samples at Former Santa Fe Railyard, Boyle Heights, CA [79], Table 2.

	CC-SV-16-B	82	11/04/2015 08:24:00	ND (<0.50)	ND (<0.50)	ND (<0.10)	ND (<0.10)	ND (<0.50)	**21**	**1.5**		
	CC-SV-16-B		08/02/2016 12:57:00	ND (<0.50)	ND (<0.50)	ND (<0.10)	ND (<0.10)	ND (<0.50)	**4.0**	**0.49**		
	CC-SV-16-B		03/08/2017 10:43:00	ND (<0.50)	ND (<0.50)	ND (<0.10)	ND (<0.10)	ND (<0.50)	**1.2**	**0.11**		
CC-SV-16-82	CC-SV-16-82', P34107cc		02/14/2013 08:54:00	ND (<0.20)	**2.3**	ND (<0.04)	**0.10**	ND (<0.20)	**440**	**31**	318	84
CC-SV-16-A	CC-SV-16-A, P24990cc		11/05/2014 08:39:00	ND (<0.50)	**3.5**	ND (<0.10)	**0.19**	ND (<0.50)	**280**	**46**		
	CC-SV-16-A		01/23/2015 07:29:00	ND (<0.50)	**4.1**	ND (<0.10)	**0.23**	ND (<0.50)	**300**	**48**		
	CC-SV-16-A		11/04/2015 08:09:00	ND (<0.50)	**2.8**	ND (<0.10)	**0.18**	ND (<0.50)	**230**	**46**		
	CC-SV-16-A		08/02/2016 12:55:00	ND (<2.5)	ND (<2.5)	ND (<0.50)	ND (<0.50)	**4.1**	**130**	**28**		
				**NA**	**NA**	**NA**	**NA**	**NA**	**NA**	**NA**		
CC-SV-17-15	CC-SV-17-15', P12762cc	15	11/05/2014 08:21:00	ND (<0.50)	ND (<0.50)	ND (<0.10)	ND (<0.10)	ND (<0.50)	**36**	**7.8**	71	19
	CC-SV-17-15		01/23/2015 08:18:00	ND (<0.50)	ND (<0.50)	ND (<0.10)	ND (<0.10)	ND (<0.50)	**35**	**6.9**		
	CC-SV-17-15		11/04/2015 09:13:00	ND (<0.50)	ND (<0.50)	ND (<0.10)	ND (<0.10)	ND (<0.50)	**60**	**9.2**		
	CC-SV-17-15		08/02/2016 11:00:00	ND (<0.50)	ND (<0.50)	ND (<0.10)	ND (<0.10)	ND (<0.50)	**9.8**	**2.4**		
	CC-SV-17-15		03/08/2017 10:15:00	ND (<0.50)	ND (<0.50)	ND (<0.10)	ND (<0.10)	ND (<0.50)	**15**	**2.9**		
CC-SV-17-45	CC-SV-17-45', P14112cc	45	11/05/2014 08:12:00	ND (<0.50)	ND (<0.50)	ND (<0.10)	ND (<0.10)	ND (<0.50)	**55**	**11**	181	48
	CC-SV-17-45		01/23/2015 08:27:00	ND (<0.50)	ND (<0.50)	ND (<0.10)	ND (<0.10)	ND (<0.50)	**61**	**11**		
	CC-SV-17-45		11/04/2015 09:26:00	ND (<0.50)	ND (<0.50)	ND (<0.10)	ND (<0.10)	ND (<0.50)	**100**	**13**		
	CC-SV-17-45		08/02/2016 10:58:00	ND (<1.0)	ND (<1.0)	ND (<0.20)	ND (<0.20)	ND (<1.0)	**21**	**5.0**		
	CC-SV-17-45		03/08/2017 09:59:00	ND (<0.50)	ND (<0.50)	ND (<0.10)	ND (<0.10)	ND (<0.50)	**19**	**3.9**		
CC-SV-17-78	CC-SV-17-78', P24930cc	78	11/05/2014 08:01:00	ND (<0.50)	**2.6**	ND (<0.10)	**0.31**	**1.1**	**290**	**48**	303	80
	CC-SV-17DR-78		11/04/2015 09:49:00	ND (<0.50)	**1.7**	ND (<0.10)	**0.14**	**0.88**	**210**	**31**		
	CC-SV-17-78		08/02/2016 11:16:00	ND (<4.0)	ND (<4.0)	ND (<0.80)	ND (<0.80)	ND (<4.0)	**46**	**8.8**		
				**NA**	**NA**	**NA**	**NA**	**NA**	**NA**	**NA**		

Notes:

Bgs	=	Below Ground Surface
DUP	=	Field Duplicate
NA	=	Denotes Sample not Analyzed
ND (<X)	=	Denotes Result was Below the Detection Limit of X µg/L
µg/L	=	Micrograms per Liter

Yellow Highlights denote concentrations above the depth specific cleanup goals
Red Highlights denote samples were not collected due to water in the line

ENSAFE

Table S3. Violations of the First Safety Benchmark, No "Response-Action" Levels or Environmental Screening Level (ESLs), Indoor Air, Former Naval Ordnance Testing Station, Pasadena, CA [90] (Table 6).

Table 6. Violations of the First Safety Benchmark, No "Response-Action" Levels or Environmental Screening Level (ESLs), Indoor Air, Former Naval Ordnance Testing Station, Pasadena, California (NOTSPA). Bold = Violations.

Contaminant	"Safe," No-Response-Action Level [1] = ESL [2] μg/m³	Detected-Contaminant Levels, μg/m³				ESL [2]	Risks of Detected-Contaminant Levels, Based on the 10^{-6} Risk ESLs			
		Generally Calibrated [3] (Uncalibrated Results) X (1.55–2.31)		Uncalibrated [4]			Generally Calibrated [3]		Uncalibrated [4]	
		Highest Results	Lowest Results	Highest Results	Lowest Results		Highest Risks[5]	Lowest Risks	Highest Risks	Lowest Risks
Carbon tetrachloride	0.47 [6]	1.1–1.6	1.1–1.6	0.679 J [7]	0.679 J [7]	10^{-6}	**(3.4) 10^{-6}**	**(2.3) 10^{-6}**	**(1.4) 10^{-6}**	**(1.4) 10^{-6}**
Dichlorodifluoromethane	0.12 [8]	2.8–4.2	0.7–1.1	1.83	0.464 J [7]	10^{-6}	**(3.5) 10^{-5}**	**(5.8) 10^{-6}**	**(1.5)10^{-5}**	**(3.9) 10^{-6}**
Perchloroethylene	0.46 [6]	20.8–31.0	2.2–3.3	13.4	1.43	10^{-6}	**(6.7) 10^{-5}**	**(4.8) 10^{-6}**	**(2.9) 10^{-5}**	**(3.1) 10^{-6}**

[1] [17,26,27,40,42]. [2] See Section 2.2.1.3, this paper. [3] See Section 2.2.1.5, this paper. [4] See Section 3.4 and Section 3.4.1, this paper. [5] Given the perchloroethylene (PCE) ESL = 0.46 ug/m³ (column 2), and the PCE ESL risk of 10^{-6}, one extrapolates to obtain the risk of various detected PCE levels. [6] State of California screening level [42]. [7] J Value is estimated because it is above the Beacon detection limit, thus clearly detected, but below the Beacon quantitation limit; as a result, it is somewhere between the detection and quantitation limits, but clearly above the residential ESL. [8] See Section 2.2.1.6, this paper.

Table S4. Violations of the Second Safety Benchmark, No Significant Risk Level (NSRL), Indoor-Air Passive Samplers, Former Naval Ordnance Testing Station, Pasadena, CA [90] (Table 7).

Table 7. Violations of the Second Safety Benchmark, No Significant Risk Level (NSRL), Indoor-Air Passive Samplers, Former Naval Ordnance Testing Station, Pasadena, California (NOTSPA). Bold = Violations.

Contaminant	"Safe," No-Response-Action Level [1] = ESL [2] 10^{-6} Risk, μg/m³	Detected-Contaminant Levels, μg/m³				Risks of Detected-Contaminant Levels, Based On The NSRL Or "Safe Harbor" Level, μg/m³ Per Day				
		Generally Calibrated [3]		Uncalibrated [4]		NSRL [5]	Generally Calibrated [3]		Uncalibrated [4]	
		Highest Results	Lowest Results	Highest Results	Lowest Results		Highest Risks [6]	Lowest Risks	Highest Risks	Lowest Risks
Carbon tetrachloride	0.47 [7]	1.1–1.6	1.1–1.6	0.679 J [8]	0.679 J [8]	5	**(6.4) 10^{-5}**	**(4.4) 10^{-5}**	**(2.7) 10^{-5}**	**(2.7) 10^{-5}**
Dichlorodifluoromethane	0.12 [9]	2.8–4.2	0.7–1.1	1.83	0.464 J [8]	not given	-	-	-	-
Perchloroethylene	0.46 [7]	20.8–31.0	2.2–3.3	13.4	1.43	14	**(4.4) 10^{-4}**	**(3.1) 10^{-5}**	**(1.9) 10^{-4}**	**(2.0) 10^{-5}**

[1] [17,26,27,40,42]. [2] See Section 2.2.1.3, this paper and [28]. [3] See Section 2.2.1.5, this paper. [4] See Section 3.4 and Section 3.4.1, this paper. [5] The NSRL of California DTSC is defined in regulations as the daily contaminant-intake level calculated to result in one excess case of cancer in a population of 100,000 exposed people [43]. Per note 2 above, we use the residential NSRL [28]. [6] Given a detected contaminant level/day, associated with the NSRL, this is the generally calibrated risk range represented by (detected level) (20 h) for residential risk and (detected level) (10 h) for commercial risk. [7] State of California screening level [42]. [8] J Value is estimated because it is above the Beacon detection limit, but below the Beacon quantitation limit; as a result, it is somewhere between the detection and quantitation limits, but clearly detected above the residential ESL. [9] See Section 2.2.1.6, this paper.

Table S5. Violations of the Third Safety Benchmark, Based on Inhalation Unit Risk (IUR), Indoor-Air Passive Samplers, Former Naval Ordnance Testing Station, Pasadena, CA [90] (Table 8).

Table 8. Violations of the Third Safety Benchmark, Based on Inhalation Unit Risk (IUR), Indoor-Air Passive Samplers, Former Naval Ordnance Testing Station, Pasadena, California (NOTSPA). Bold = Violations.

Contaminant	"Safe," No-Response-Action Level [1] = ESL [2] 10^{-6} Risk, $\mu g/m^3$	Detected-Contaminant Levels, $\mu g/m^3$				IUR [5]	Risks of Contaminant Levels, Based on the IUR $\mu g/m^3$			
		Generally Calibrated [3]		Uncalibrated [4]			Generally Calibrated [3]		Uncalibrated [4]	
		Highest Results	Lowest Results	Highest Results	Lowest Results		Highest Risks [6]	Lowest Risks	Highest Risks	Lowest Risks
Carbon tetrachloride	0.47 [7]	1.1–1.6	1.1–1.6	0.679 J [8]	0.679 J [8]	(4.2) 10^{-5}	**(6.7) 10^{-5}**	**(4.6) 10^{-5}**	**(2.9) 10^{-5}**	**(2.9) 10^{-5}**
Dichlorodifluoromethane	0.12 [9]	2.8–4.2	0.7–1.1	1.83	0.464 J [8]	not given	-	-	-	-
Perchloroethylene	0.46 [7]	20.8–31.0	2.2–3.3	13.4	1.43	(6.1) 10^{-6}	**(1.9) 10^{-4}**	**(1.3) 10^{-5}**	**(8.2) 10^{-5}**	**(8.7) 10^{-6}**

[1] [17,26,27,40,42]. [2] See Section 2.2.1.3, this paper. [3] See Section 2.2.1.5, this paper. [4] See Section 3.4 and Section 3.4.1, this paper. [5] The Inhalation Unit Risk (IUR) of US EPA "is an estimate of the increased cancer risk from inhalation exposure to a [contaminant] concentration of 1 $\mu g/m^3$ for a lifetime" [49]. [6] Given the perchloroethylene (PCE) IUR = 6.1×10^{-6} (column 7), the PCE inhalation risk for the value of the highest generally calibrated PCE detection of 31 $\mu g/m^3$ (column 3) = (PCE IUR) (PCE detected value whose risk we want to know) = (0.0000061) (31) = 0.00019 or 1.9×10^{-4} inhalation cancer risk. [7] State of California screening level [42]. [8] J Value is estimated because it is above the Beacon detection limit, but below the Beacon quantitation limit; as a result, it is somewhere between the detection and quantitation limits, but above the residential ESL. [9] See Section 2.2.1.6 of this analysis.
