# Supplementary material for: Does Hazardous-Waste Testing Follow Technical Guidance, Thus Help Protect Environmental Justice and Health?
Source: Int J Environ Res Public Health. 2022 Jun 23;19(13):7679. doi: 10.3390/ijerph19137679 (PMC9265884; doi:10.3390/ijerph19137679)
Supplement: Supplementary file 1 [file ijerph-19-07679-s001.zip › 6-2-22-ksf-Supplemental S2.pdf]

Supplementary Materials S2, Including All References

2.1 US CERCLA and Brownfields Sites and Why They Have Not Been Tested and Remediated

Under Superfund, the US Comprehensive Environmental Response, Compensation, and Liability Act (CERCLA), there are two main types of US hazardous-waste facilities: National Priorities (NPL)/Superfund installations and brownfields. NPL sites are federally remediated under the US Environmental Protection Agency (US EPA) and have "national priority among the known releases or threatened releases of hazardous substances" [11]. They must follow CERCLA testing/cleanup rules. Brownfields, typically remediated through state programs, are properties with "the presence or potential presence of a hazardous substance" [118]. Brownfields (that are no longer continuing their operations) usually follow Resource Conservation and Recovery Act (RCRA) testing/cleanup rules that are less demanding than CERCLA rules [119]. However, US military installations, whether owned/operated by private defense contractors or the armed services, can be either NPL or brownfields (RCRA) sites [120]. As CERCLA allows [111] (p. 12), the former Naval Ordnance Testing Station, Pasadena, California (NOTSPA) is a state-cleanup brownfield [7], but must follow CERCLA testing/remediation rules, given its serious contamination [88]. Sites like NOTSPA show there is no mutually exclusive distinction between brownfields and CERCLA/NPL sites.

US EPA estimates that US brownfields number up to 1,000,000 [14], of which only a small fraction have been remediated (which requires prior testing). Of these brownfields, EPA has remediated 2,180; state programs have remediated another 190,050; and 8,871 are "ready for reuse" but not remediated [15]. Those in the last category have not been remediated because state regulators claim either that toxins have been reduced [16], that the wastes are unlikely to cause significant effects, or that the economic benefits of immediate site reuse "outweigh… the significant and unavoidable impact" of contaminants [17] (p. 18). Given US EPA's total-brownfields estimate (up to 1,000,000), roughly 19% (192,230 of 1,000,000) have been remediated.

Of 1,714 US NPL sites, only 387 or 23% have been tested and remediated [11, 12, 13]. Most of those unremediated are military sites [122, 114 (p. 11)]: facilities of military contractors such as Boeing; armed-services bases, laboratories, or test sites, like NOTSPA; or US nuclear-weapons facilities that constitute "the largest environmental cleanup program in the world" [123]. Because NPL and military sites are the most contaminated and

difficult to remediate, most unremediated NPL sites were added to the list more than 30 years ago, during the first 10 years after 1980 CERCLA passage [12].

At least 6 reasons help explain US toxic sites' incomplete testing and remediation.

First, testing and cleanup are ***expensive***, "costly and time-intensive," not "practical" [58] (pp. 47, 51), claims Trammell Crow Company (TCC) the largest US commercial developer [10]. Expensive testing/cleanup is why multiple prospective purchasers rejected NOTSPA. TCC's own tests show that NOTSPA soil concentrations of volatile organic compound (VOC) chlorinated solvents, such as trichloroethylene (TCE), perchloroethylene (PCE), carbon tetrachloride (CT), and dibromochloromethane---the four site "risk drivers" [6](p. 34)---are up to nearly one million times above allowed levels [59] (see Table S1 in Appendix 1). As a result, NOTSPA faces vapor intrusion (VI) [5], migration of subsurface gaseous carcinogens like CT, PCE, and TCE into surface buildings [128]. VI sites have reported increases in rates of cancer, low-birthweight children, restricted fetal growth, and cardiac defects, although only a few epidemiological studies have fully confirmed VI causality, given background co-exposures, eg., [5, 27, 30, 125,127,128].

Second, remediation often does not occur because responsible parties use anti-regulatory ***legal maneuvers*** to avoid teting/cleanup. For instance, given suspected VI that threatens NOTSPA renters, California Department of Toxic Substances Control (DTSC) issued a 2004 "Imminent and Substantial Endangerment" order and demanded cleanup [87, 123]. Yet despite no cleanup, DTSC was forced to drop the order, a condition demanded by the responsible parties before they would enter negotiations with DTSC. Because negotiations failed [128], NOTSPA remains contaminated.

Third, both state and federal cleanups face ***inadequate funding***. Federal/CERCLA remediation monies have fallen by half since 1995 because the tax (on the petrochemical industries) that financed Superfund expired in 1995 and was not renewed [12]. Because all US states face prohibitive toxics-cleanup costs, all have adopted privatized programs through which most remediation occurs. Privatized schemes include US EPA's so-called "voluntary agreement" program[19]. In voluntary programs, private parties (usually redevelopers) negotiate testing/cleanup levels with a state, in exchange for US EPA-funded financial and regulatory incentives [19], such as tax credits, site-liability protection, allowing land-use controls, and letting the redeveloper choose a licensed private party, not the state, to conduct testing/cleanup oversight [129].

Fourth, as the US Government Accountability Office (GAO) warns, frequently full testing/cleanup does not occur because of ***the absence of interagency agreements.*** EPA is able to enforce toxic-site remediation only if other agencies, such as the Department of Defense (DOD), agree to report hazardous facilities to it [121] (p. 22). Yet few incentives exist for agencies to report toxic sites for whose cleanup they are financially liable.

Fifth, testing/remediation often does not occur because US EPA's list of hazardous facilities includes only those with reported releases. Any ***unknown or untested toxic sites*** have no reported releases, thus are not on the list [111].

Sixth, ***outdated cleanup regulations*** have not kept pace with scientific knowledge, as illustrated by NOTSPA contaminants like VOCs and perfluoroalkyl substances (PFAS) that cause vapor intrusion (VI) [130, 131]. As a result, US EPA warns that at least one quarter (up to 250,000) of estimated US toxic sites [14], poses VI threats [20]. As a result, hundreds of thousands of facilities, once considered clean, now must be reevaluated, tested for VI [31, 32, 45, 132, 133].

2.2 Privatization and Why Many Threats at US Hazardous-Waste Sites Are Unknown

Besides incomplete testing and remediation, at least three facts suggest that many toxic-waste threats are unknown because of incomplete government data.

First, despite US EPA's 2020 estimate of up to 1,000,000 US hazardous sites [14], US GAO warned the number is "unknown, due primarily to ***incomplete and inaccurate data***" (authors' bold) and that "it will take decades to complete the inventory" [121], partly because US EPA's estimates come from other agencies that have inadequate data. For instance, the Bureau of Land Management of the Department of the Interior (DOI) lists more than 30,000 abandoned unassessed mines on its lands, but only 4,722 contaminated sites. Although GAO's latest estimate is that up to 500,000 abandoned US mines require cleanup [134], many of them must be added to US EPA's list of hazardous facilities [111].

Second, even when toxic sites have been assessed/remediated/closed, US GAO warns that ***assessments often are incomplete*** [121], partly because it cannot document safe cleanup at 40 percent of supposedly remediated military facilities [135]. DOD's Inspector General agrees, citing fraudulent cleanup contractors [136] like Tetra Tech. It took $1 billion from the US Navy to remediate Hunter's Point Naval Station in San Francisco. Yet after thousands of mostly African-Americans moved onto the redeveloped site and reported disproportionate numbers of cancers, the Navy and US EPA discovered Tetra Tech had falsified 97% of cleanup data [137, 138, 139, 140].

Third, many toxic-site problems also are unknown because, except for CERCLA facilities, most US hazardous-waste locations have ***privatized*** [18, 15], including voluntary-program cleanups, including testing [19, 129]. This remediation often occurs with minimal state oversight/knowledge. New Jersey and Massachusetts, for instance, have two of the longest established, privatized, voluntary-cleanup programs, yet their results suggest significant safety problems. For instance, every year, 20% of Massachusetts' privatized cleanups must receive state-government audits, of which only 2% undergo soil sampling [18]. Yet annually 87-71% of audited, privatized, Massachusetts toxic-site cleanups, numbering roughly 1,800 or more locations per year, fail this audit [18, 33]. If the unaudited 80% of privatized, Massachusetts cleanups undergo similar poor remediation, this means the numbers of risky, but supposedly clean, toxic sites are substantially greater than 1,800 per year. Why? Most threats are neither detected nor remediated because most sites are not audited. Such failures help explain why attorneys say private remediators "routinely" violate regulations and create public-health risks [18]. Every year as many as 21% of audited, privatized, Massachusetts cleanups (479 locations) pose such urgent health threats that the state invalidates their closures ; if these data are representative, they suggest that the remaining 80 percent of annual, unaudited, supposedly remediated toxic sites could include nearly 2,000 Massachusetts locations whose closures threaten public health and should be invalidated [33].

In New Jersey two reasons suggest privatized site remediation is more problematic than in Massachusetts. One reason is that only 10% of hazardous-facility, privatized-facility cleanups receive annual audits, not 20%, as in Massachusetts. Another reason is that New Jersey has no voluntary-program cleanups, overseen by the state; instead, to save money, the entire cleanup and regulatory process is privatized, with persons hired by the interested parties being responsible for choosing testing/cleanup options, a situation that creates a conflict of interest [18].

If the preceding data are representative of most states, where no routine scientific-data audits occur, then other privatized cleanups, as at NOTSPA, may be questionable because site assessors and remediators know that no one will officially check on them according to specific, prescribed standards. At the very least, the safety of many privatized, hazardous-waste cleanups is unknown.

Although negotiated, privatized schemes dominate remediation in every state [19, 132], the schemes differ, state to state, depending on the degree to which the state privatizes, or outsources, various testing/cleanup functions [18, 33]. Such functions include control of (1) permitting, (2) scope of testing/cleanup, (3) testing, (4) cleanup, (5) oversight, (6) certification of cleanup, and (7) enforcement [33].

In the privatized programs of Connecticut, Massachusetts, and New Jersey, the state controls functions (1) and (7), outsources (2)-(6) [18, 33], then guarantees it will not sue the assessors, remediators, and redevelopers, provided they follow required standards [141]. However, as preceding paragraphs illustrated, Massachusetts has a more robust enforcement/auditing program than New Jersey, and most states have no such post-cleanup audits [18, 33] In California, the state controls functions (1) and (5)-(7). However, in exchange for liability protection, streamlined regulatory responses, and financial incentives, private redevelopers may negotiate (2)-(4), reduced toxic-site testing and cleanup [141, 142].

Mainly because private redevelopers negotiate reduced toxic-site testing/cleanup, the success of privatized cleanups is controversial. On one hand, most states focus on economics and claim that privatized programs have saved money, reduced cleanup time, and increased brownfields redevelopment. Within several years, New Jersey's program greatly increased the pace of testing/remediation and closed 6,000 of 20,000 active hazardous sites [143]. Similarly, Massachusetts remediated "thousands more sites per year" than its earlier, non-privatized program [18]. On the other hand, public-health and legal experts say that state oversight of privatized testing/cleanup is inadequate; that "remediation is typically driven by purely financial motives"; and that private interests routinely "conduct a partial investigation or remediation without penalty" [18].

Such a privatized, partial investigation occurred at the TCE- and PCE-contaminated site in Franklin, Indiana. As a result, in the last 11 years Franklin families have reported 58 children in their small town with rare blood and brain cancers, although epidemiological studies have not been completed. During the supposed Franklin cleanup, US EPA and the state retained control of (1) permitting, (5) oversight, (6) cleanup certification, and (7) enforcement, but outsourced to private interests (2) the scope of testing/cleanup, (3) testing, and (4) cleanup. However, the private interests ignored state/federal scientific guidance to test for offsite-VOC migration and VI---testing that might have prevented the town's increase in reported child cancers. Typical of most US privatized cleanups, until children began dying, no officials investigated the quality of the supposed testing/remediation [125].

2.3 Why Hazardous-Waste Cleanup Does Not Correct Environmental Injustice

Despite the preceding, documented, limited oversight of privatized testing/cleanup, US EPA relies on little evidence when it touts brownfields remediation and redevelopment as a way to correct environmental injustice [144].

However, because hazardous facilities tend to be located in environmental-justice communities, hurt by the toxins, therefore EPA gratuitously assumes that brownfields remediation will protect these communities eg, [8, 9, 145, 146].

Contrary to US EPA's assumption, the literature shows that testing/remediation often fails to serve environmental-justice communities because toxic-site redevelopers tend to drive privatized cleanups, based on what is most profitable, not what ensures environmental justice. Given reliance on market mechanisms and the absence of federal statutory requirements to consider environmental injustice [138], privatized cleanups tend to employ less protective cleanup standards. Although privatized programs typically give redevelopers immunity from toxic-site liability, in exchange for partial testing/remediation, redevelopers frequently fail to address contamination, thus worsen environmental injustice [147, 148].

Post-cleanup gentrification also is a problem. Because supposed cleanup tends to increase percentages of white and non-poor populations living on former toxic-waste sites, it can worsen inequity and the plight of marginalized populations [149, 150, 151]. Genuine remediation and redevelopment also are less likely to occur in poorer, largely minority communities than in wealthier, non-minority communities. When partial cleanup does occur in poorer locations, it typically proceeds much more slowly than in wealthier areas [8, 147, 148].

2.4 The Weight of Evidence (WoE) Method

In part to protect environmental-justice communities and ensure defensible science, how does one evaluate the adequacy of toxic-site assessments and cleanup? Although current epidemiological methods are reliable, largely because they are very narrow, they cannot be used for such an evaluation. Because of their narrowness, they are unable to evaluate either the adequacy of multi-disciplinary, toxic-site cleanups or the degree to which they protect environmental-justice and affected communities.

Except for US EPA's systematic 2016, three-part, weight-of-evidence (WoE) method, scientists have virtually no transparent, systematic method for integrating/weighing multiple pieces of diverse, often-conflicting lab/field/statistical/epidemiological/modeling evidence, in order to infer likely causality/teratogenicity/safety/needed regulatory action [34], based on examining all available/relevant positive and negative evidence. WoE dictates (1) assembling a body of positive/negative evidence regarding some question, e.g., which of two hypotheses is superior; (2) justifying evidence-weighting/scoring procedures; and (3) evaluating this weighting/scoring to answer the relevant question [34](p. 1).

WoE is widely used, primarily by US EPA/risk/environmental-impact/hazardous-site assessors and is based on Bradford-Hill considerations [34]. Given diverse, conflicting, and incomplete evidence, US EPA recently used WoE, for instance, to weigh this evidence and infer the teratogenicity of TCE [41] and to impose TCE Interim/Early Action Mandates on Superfund sites [42].

Precisely because WoE evaluates many diverse, often conflicting, pieces of evidence, it can tentatively answer questions that are more policy relevant than those of most statistical or epidemiological methods, questions such as whether a hazardous-waste site is safe, or whether a particular chemical should be regulated, questions that cannot be answered by a single lab/field/statistical/epidemiological/ modeling method [34]. No single statistical or empirical method can assess, for instance, which of two diverse-evidence hypotheses/theories is more reliable, because there are **no specific/quantitative methods or methodological rules** for such hypothesis/theory assessment. Instead, each theory-assessment situation is partly different, requiring comparison/interpretation of different evidence from different sources. Thus one can only argue, not show quantitatively or empirically, that such comparisons/interpretations of theory-relevant methodological rules/values (such as predictive power, coherence, etc) are tentatively correct, unless one refers to other scientists' expectations, which (by definition) are not empirical [44].

Yet because WoE is not quantitative or empirical in the traditional sense, but meta-scientific/analytical, it can more easily be used badly or arbitrarily than can narrower scientific methods. To avoid such arbitrariness, we follow four US EPA-recommended, WoE strategies:

- comparing which of two same-subfield theories/hypotheses is more credible, rather than whether a single theory/hypothesis is credible [34] (pp. 16, 39). Thus, our study asks whether privatized soil-gas tests tend to follow or to violate government testing guidance.
- specifying all WOE methods, procedures, strategies, ahead of time [34] (p. 4), as we do in 2.5 below.
- using a program or field's own criteria to assign weights/scores to evidence [34] (p. 4). Thus, our study assigns weights in WoE Part 2, based on what DTSC and US EPA guidance call testing "requirements."
- being transparent and systematic in specifying strategies for each of the 3 stages of WoE [34] (pp. 4-5).

Thus, our study specifies and defends 7 WoE Part 1 selection criteria for hazardous sites to evaluate, and 6 WoE Part 2 requirements for soil-gas testing, based on what guidance calls requirements.

2.5 WoE Part 1.1: Strategies to Find Federal and State of California Guidance on VI Testing

2.5.1 The Strategy for Federal Documents

The first part of the first (or federal) document-search strategy for WoE Part 1.1 is to go to <epa.gov/vaporintrusion>, then to the website header, “Current Guidance,” then to download documents listed under it. The second part of the first strategy is to go to “Vapor Intrusion at Superfund Sites,” at <epa.gov/vaporintrusion/vapor-intrusion-superfund-sites#:~:text=hazard%20ranking %20system-,Trichloroethylene%20and%20Vapor%20Intrusion,non%2Dcancer%20human%20health%20hazards>, then to download all guidance documents that appear.

2.5.2 Strategy for State of California Documents

The second (or state) document-search strategy is more complex than the federal strategy because California VI-guidance documents are spread among the different CAL-EPA offices that generated each of them. Thus, one must employ at least 3 separate/mutually exclusive sub-strategies to find all documents:

- Go to <dtsc.ca.gov/vapor-intrusion/>, use the search term “guidance,” then download all non-duplicate, latest-version records listed under “VI guidance documents” (Search Strategy 1, WoE Part 1.1).
- Go to <dtsc.ca.gov/vapor-intrusion/>, then to the header, “Site Mitigation and Restoration Program Links,” then click on “Proven Technologies and Remedies Documents,” which leads to <dtsc.ca.gov/proven-technologies-remedies-documents/>. At the previous link, click on “Remediation of Chlorinated VOCs in Vadose Zone Soil,” then download all guidance documents for subsurface-VOC tests (Search Strategy 2, WoE Part 1.1).
- Go to <dtsc.ca.gov/vapor-intrusion/>, then to the header, “Site Mitigation and Restoration Program Links,” then click on “Human and Ecological Risk Office (HERO),” which leads to <dtsc.ca.gov/assessing-risk/ >. At the preceding link, click on “Human Health Risk Assessment,” which leads to <dtsc.ca.gov/human-health-risk-hero/>. At the preceding link, under the header “Human Health Risk Assessment Guidance,” download all (but only) guidance documents for VOCs, soil-gas, or VI testing (Search Strategy 3, WoE Part 1.1).

2.6 WoE Part 1.1: Strategies to Find Hazardous-Waste Sites Whose VI-Testing Documents to Evaluate

To find California toxic sites whose documents to evaluate for conformity to testing guidance, one can begin by using two prominent California databases. EnviroStor <dtsc.ca.gov/your-envirostor/> is the DTSC's premier online data-management system. It tracks state-controlled (not federal) hazardous-facility assessment, cleanup, permitting, and enforcement, including US military sites with known/suspected contamination. CEQAnet <ceqanet.opr.ca.gov/> is the online, searchable database of the State Clearinghouse for California Environmental Quality Act (CEQA) documents that provide cumulative state/local environmental review of proposed actions/projects that involve federal financial assistance or development documents [152]. CEQAnet is not a comprehensive database, as most toxic sites (required to follow CEQA) have no federal financial/development assistance, thus have no documents in CEQAnet [146].

Unfortunately, EnviroStor and CEQAnet databases have limited filters that allow no searches for most criteria/key words in our hypothesis. As a result, one cannot use the databases to search for toxic sites that satisfy the 7 selection criteria (for WoE, Part 1.2), listed in the text:

i **California locations**, subject to state guidance;

ii **sampling performed by/for TC**, the largest US commercial developer [10], who "pioneered…privatized remediation" [40] and is "the industry leader in Brownfields development" (see Appendix 2 (2.6);

iii **recent testing/remediation**, conducted since 2011, when California's main subsurface-VOC/VI guidance appeared [21];

iv **publicly accessible documents**, on Envirostor;

v **subsurface-trichloroethylene** (TCE) contamination;

vi multiple subsurface-**VOC carcinogens**; and

vii **privatized cleanup**.

In Envirostor, one can search for only the fifth (v) of our selection criteria (TCE sites), but if one then searches by year, or by using any successive search terms (e.g., Trammell Crow, TCC, TCC, VOC, etc.), each successive search produces 0 results. Even advanced Envirostor searches allow only name, address, assessor's parcel number, census tract, school district, cleanup program (e.g., NPL, voluntary, etc.), funding, contaminant, past uses (e.g., aerospace rocket testing), and media affected <envirostor.dtsc.ca.gov/public/search>. One can search Envirostor for voluntary cleanups, but because much privatized cleanup is not voluntary (see the Introduction in the text), this search fails to

capture many privatized cleanups. Moreover, Envirostor's 14,000+ toxic sites (and even its 926 sites with known TCE) are too many to search by hand, to check whether each site's roughly 20 documents meet all 7 selection criteria. <envirostor.dtsc.ca.gov/public/search?CMD=search&ocieerp=&HWMP=False&business_name=&main_street_name=&city=&zip=&county=&censustract=&case_number=&apn=&CRITICAL_POL=30027&Search=Get+Report>.

Similar problems beset CEQAnet. Like Envirostor, CEQAnet has limited filtering/search capacities. CEQAnet permits searches for only 2 of the preceding 7 criteria, the first ("Trammell Crow") and the third (by year). Yet such searches do not allow hypothesis assessment because CEQAnet contains only a small percentage of toxic sites and associated documents.

Because of CEQAnet incompleteness and the inability to search either Envirostor or CEQAnet by using most of the 7 selection criteria, Envirostor and CEQAnet cannot generate a reliable list of current TCC California cleanups that meet our 7 toxic-site, selection criteria. To generate such a list, one needs at least 4 interdependent, successive strategies, only the last of which generates the final results:

- *Strategy 1*: employ (a) CEQAnet <ceqanet.opr.ca.gov/> and the two online (b) TCC lists of California toxic sites that TCC is testing, remediating, or redeveloping [154, 155], so as to generate a **preliminary, unedited toxic-site list** of California redevelopment projects that meet the first 2 of 7 selection criteria.
- *Strategy 2*: use Strategy-1 results to conduct internet searches for addresses of each Strategy-1 site, then use these addresses to strike all differently named, same-address, duplicate sites, thus generate a **preliminary, nonduplicate toxic-site list** of locations that meet the first 2 selection criteria.
- *Strategy 3*: use each Strategy-2 address to search Envirostor <dtsc.ca.gov/your-envirostor/> and CEQAnet, then download/examine all official documents for each site. Remove any sites that are not redevelopments of installations that state or federal governments list as former/existing hazardous facilities, then use the resulting inventory to generate a **preliminary, nonduplicate, toxic-site list** of redevelopment projects, based on the first 2 of 7 selection criteria.
- *Strategy 4*: use all Strategy-3 downloads to find all testing-related documents for each site; assess each document, so as to determine which hazardous facilities meet the last 5 of the 7 selection criteria; and thus generate a **final toxic-site list**, based on all 7 selection criteria.

To implement preceding Strategy 1, we used the search term "Trammell Crow" in (a) CEQAnet and found 23 entries. To find (b) lists of California TCC assessment/remediation/redevelopment sites, we went to the website of

TCC Vice President (Robert Chute), in charge of all Environmental Risk Management and hazardous locations; he lists 6 toxic-site projects [155]. Because TCC is both the largest US commercial developer [4] and "the industry leader in Brownfields development" [154], next we went to the website of TCC's major unit, the brownfields unit, Brownfields Acquisition and Development (BAD), within the environmental-risk or EASI (Environmental Asset Services, Inc.) Division; it contains a list of 26 sites [154]. Strategy 1 thus provides a list of (CEQAnet+Chute+EASI) projects numbering (23+6+26), 55 in all. Strategy 1 thus generates a 55-item **preliminary, unedited, toxic-site list** of TCC California redevelopment projects that meet the first 2 of our 7 criteria.

We implemented Strategy 2 to eliminate Strategy-1 duplicates. (Duplicates occur because TCC lists its toxic sites in inconsistent ways: by pre-redevelopment, post-redevelopment, informal, or LLC names; or combinations of the preceding labels [154, 155].) Strategy 2 generates a **25-item** (9+5+11) list, respectively, of (CEQAnet+Chute+EASI) projects: a **preliminary, nonduplicate, toxic-site list** of TCC projects that meet our first 2 selection criteria.

We implemented Strategy 3 by using Strategy-2 addresses as search terms, downloading Envirostor/CEQAnet documents for the 25 sites, then examining the documents, so as to strike (from our Strategy-2 list) all projects not at current/former toxic sites. Strategy 3 thus generates 11 (CEQAnet+Chute+EASI) or (2+2+7) toxic-site redevelopments ---an **11-item**, TCC, California, **preliminary, nonduplicate toxic-site list**, based on the first 2 selection criteria**.**

To implement Strategy 4, we assessed Strategy-3 findings (documents from all TCC toxic-site redevelopments) to determine which projects meet all 7 selection criteria. The Results presents our WOE Part 1 findings: a **final toxic-site list**, based on all 7 selection criteria.

2.7 How Can Government Guidance Impose Requirements

Of course, the main purpose of generating the preceding list of toxic sites is to provide an objectively determined inventory of hazardous-waste facilities whose assessment and remediation to evaluate. In order for this evaluation to answer the main question of the paper, the evaluation must determine whether actual toxic-site testing meets government testing requirements. However, this evaluation raises the question: **How can guidance (which is not part of regulations) impose testing requirements** [21]? We ask and answer this question because, in response to our criticisms that TCC violates DTSC technical-guidance testing requirements [95], DTSC's NOTSPA project director wrote that NOTSPA testing "satisfies current regulatory requirements and enforceable cleanup standards" [95] (p. 192). That is, he appears to accept only legally enforceable requirements, but not scientific requirements

mandated by government technical guidance. If he is correct, then this paper is incorrect in evaluating adherence to testing-guidance requirements, as there are no such requirements.

On the contrary, as the text's discussion of methodological assumptions suggests, we believe that US EPA/DTSC VI guidance mandates technical requirements for toxic-site testing [46]. These technical requirements [21] "clarify … implementation of the regulations" [156] but, of course, impose no "inflexible requirements" [21], because they must be flexible enough to incorporate ongoing scientific progress in testing methods. That is, scientific/technical guidance imposes ***default technical requirements*** that are binding---unless scientists explicitly demonstrate better or technically equivalent test procedures. If assessors do not follow (what DTSC calls) its testing "requirements," then DTSC technical guidance says assessors' "alternative approaches and technologies should be supported by adequate technical documentation" [45] (p. 2); see [157].

Because TCC provides no justification for any of its testing violations/alternative test methods---but instead misrepresents its testing as adhering to the highest standards, its testing violations are indefensible. Why? Contrary to DTSC technical guidance [45] (p. 2), TCC provides no "technical documentation" to justify its violations of requirements and its use of alternative tests. Moreover, TCC likely cannot justify its violations because the Results and Question-1 discussion in the text show that all TCC testing violations cause risk underestimates; yet, by definition, contaminant/risk underestimates cannot provide testing that is "technically equivalent" to what DTSC requires [45, 21].

2.8 How Trammell Crow Violates DTSC Risk Calculations for Cancer

In addition to underestimating NOTSPA-site risk as a result of testing violations, TCC also underestimates site risk as a result of technical-calculation violations. It *violates 6 cancer-calculation rules,* mandated by DTSC for toxic-site Human Health Screening Evaluations (HHSEs) [21, 49, 50]. In doing so, it undercalculates NOTSPA cancer risks by a factor of nearly 100. This undercalculation is a result of TCC's providing:

(1) no calculations of **unmitigated risk,** required by DTSC [21, 50]; instead, it gives only estimated mitigated site risk, after presumed removal of metals hotspots/drains [6], some never tested [6] (see Figure S1 in Appendix 1).

(2) no calculations for **"the maximum measured indoor-air concentration of each chemical,"** required by DTSC [21 (p. 35), 49, 50] (authors' bold). Instead, TCC uses an illegitimate assumption, rejected by DTSC [21 (pp. 21, 55), 45 (p. vii)] that at a site like NOTSPA, with subsurface preferential VI pathways, indoor-air carcinogenic gases

are only 0.001 of soil-gas levels. This faulty assumption causes an illegitimate, 50-fold underestimate of NOTSPA indoor-air VOCs [21, 45].

(3) no risk calculations for **highest-level, any-depth site toxins,** required by DTSC [21, 50], as VI can arise from from toxins 100+ feet subsurface [21, 45]; instead, it provides only highest-level toxins from 30 feet subsurface [6].

(4) no total cancer risk, based on "**all lines of evidence"** (authors' bold), as DTSC requires, from ambient air, ground-water, indoor air, soil, soil gas [21, 50]; instead, it omits groundwater, indoor-air, and ambient-air risks [59].

(5) no calculation for the **site-attenuation factor,** required by DTSC, [21]. Instead, it uses an outdated, default-factor attenuation (0.0001, not 0.05) [6] that illegitimately reduces indoor risks by a factor of 500 [45, 21]

(6) no risk for **maximum toxin concentrations,** required by DTSC [21, 50]; instead, it uses the average concentration for the 95th percentile Upper Confidence Limit (UCL) [6], that illegitimately reduces risks by roughly 100 [85].

With respect to (6), health threats from TCC's undercalculating cancer risks, **for 2 reasons DTSC mandates that HHSEs must use maximum-concentration levels of all site contaminants** [21 (p. 16), 50].

First, HHSEs rely on **limited sampling** [50] (p. 15). For instance, NOTSPA-VOC sampling avoided the three typically highest VOC-contamination areas: deep soil, under (sub-slab) buildings, and toxin sources [21, 45, 48]. It tested no soil within 180 feet above groundwater [6, 59]; conducted no sampling under 89% of NOTSPA buildings [6]; and identified only low-level, shallow-soil sources for only 2 of the 35 VOC contaminants [6]. This illustrates why DTSC thus warns that because HSSE screening sampling, by definition, has no "robust dataset" of "at least…. eight subsurface samples for a single building" [21] (p. 23), and because "a robust dataset is needed for statistical approximation" [21] (p. 23) and employing UCLs, HHSEs should use "maximum subsurface concentration data" [21] (p. 24).

Second, by definition, HHSEs employ only screening tests, thus include **no randomization** in sampling strategies [50] (p. 15) unlike epidemiological or probabilistic risk assessments. Yet any statistical interpretation of HHSE results requires such randomization [158] (p. 13). For both preceding reasons, DTSC warns that "unless the data are sufficiently robust for statistical approximation of the average concentration, the maximum measured" toxin concentrations should be used [21] (p. 35), and "the 95 percent upper confidence limit (95% UCL) on the arithmetic mean" should not be used [50] (p. 15). Yet because TCC uses NOTSPA average concentrations for the 95 percent UCL, it underestimates risks, violates DTSC cancer-calculation rules, and makes an elementary error: confusing the statistics available for full

grid/random sampling with those for screening sampling. This error, apparently designed to make TCC's flawed sampling look statistically robust, instead underestimates risks.

References used in both the text and Appendices 1a, 1b, 1c, and 2 (supplemental materials)

1. Bonner, C.A.; Bonner, A. C., Attorneys for Plaintiffs. Case No.: CGC-18-566188, First Amended Class Action Complaint: Bayview Hunters Point residents, Danielle Carpenter, Catherine Muhammad, including all parties listed in paragraph 15 and incorporated herein; and Doe Plaintiffs 1-40,000, on behalf of themselves, and all others similarly situated, Plaintiffs, vs. Tetra Tech EC, Inc.; Tetra Tech, Inc; Dan L. Batrack, in his individual and official capacity, Chairman, Chief Executive Officer and President of Tetra Tech; Steven M. Burdick, in his individual and official capacity, Executive Vice President, Chief Financial Officer of Tetra Tech; Stephen C. Rolfe, in his individual and official capacity, Managing Agent of Tetra Tech; Justin E. Hubbard, in his individual and official capacity, Managing Agent of Tetra Tech; Lennar, Inc.; and Five Point Holdings, LLC., and Does 1-100 inclusive, Defendants. San Francisco: Superior Court of California, County of San Francisco, Unlimited Jurisdiction; July 2, 2018; Available from: hunterspointcommunitylawsuit.com/wp-content/HPLFIRSTAMENDEDCOMPLAINTW-EXHIBITS.pdf . Accessed 2 February 2022.
2. Powers, M.B. U.S. Joins Suits Citing Tetra Tech Fraud In Navy Site Cleanup. Engineering News Record. January 23, 2019. Available from: enr.com/articles/46260-us-joins-suits-citing-tetra-tech-fraud-in-navy-site-cleanup . Accessed 15 January 2022.
3. Aragón, T. Cancer Incidence Among Residents of the Bayview-Hunters Point Neighborhood, San Francisco, California, 2008—2012. San Francisco: San Francisco Department of Public Health; March 6, 2019. Available from: sfdph.org/dph/files/listenshareconnect/3-7-19_BVHP_Cancer_Incidence.pdf . Accessed 18 January 2022.
4. Trammell Crow Company (TCC). Corporate Information. 2021. Available from: trammellcrow.com/en/about/corporate-information. Accessed 9 Aug 2021.
5. United States Naval Ordinance Test Station China Lake and Pasadena, California. Basic Information about NOTS. Publishing Division, Technical Information Department. 1962. Available from: chinalakealumni.org/Downloads/1962%20D89TS62-168%20Basic%20Info%20NOTS.pdf. Accessed 5 Apr 2021.
6. World Health Organization (WHO). Environmental Health Inequalities Resource Package. Copenhagen: WHO Europe; 2019. Available from: euro.who.int/__data/assets/pdf_file/0018/420543/WHO-EH-inequalities-resource-package.pdf. Accessed 5 Apr 2021.
7. California Department of Toxic Substances Control (DTSC). Naval Information Research Foundation (19970020). CalEnviroScreen. 2021. Available from: envirostor.dtsc.ca.gov/public/profile_report?global_id=19970020. Accessed 5 Apr 2021.
8. California Office of Environmental Health Hazard Assessment (OEHHA). Linguistic Isolation. 2021. Available from: oehha.ca.gov/calenviroscreen/indicator/linguistic-isolation. Accessed 5 Apr 2021.
9. Forand, S.P.; Lewis-Michl, E.L.; Gomez M.I. Adverse Birth Outcomes and Maternal Exposure to Trichloroethylene and Tetrachloroethylene through Soil Vapor Intrusion in New York State. Environ. Health Perspect. 2011, 120(4), 616–621. doi.org/10.1289/ehp.1103884.
10. Ninyo and Moore. Remedial Investigation and Feasibility Study: Former Naval Information Research Foundation Undersea Center. 2017. Available from: envirostor.dtsc.ca.gov/public/deliverable_documents/3954603433/Final%20RI.FS%20report11.2018.pdf. Accessed 30 Aug 2021.
11. United States Environmental Protection Agency (US EPA). Superfund: National Priorities List (NPL). 2021. Available from: epa.gov/superfund/superfund-national-priorities-list-npl. Accessed 5 Apr 2021.
12. Morrison, J. Polluted Sites Linger under U.S. Cleanup Program. Chemical and Engineering News. 2017. Available from: cen.acs.org/articles/95/i14/Polluted-sites-linger-under-US-clean-up-program.html. Accessed 5 Apr 2021.

13. Peak, C. Seven Environmental Disasters. Nationswell. 2020. Available from: nationswell.com/superfund-contamination-sites-cleaned-up/#:~:text=Since%20its%20inception%20in%201981,has%20weakened%20the%20EPA's%20efforts. Accessed 5 Apr 2021.

14. United States Environmental Protection Agency (US EPA). Brownfields FAQs. 2020. Available from: epa.gov/brownfields/frequently-asked-questions#:~:text=The%20original%20concept%20of%20a,or%20other%20types%20of%20sites.&text=Since%20GAO's%20estimate%20remains%20the,450%2C000%20%E2%80%93%201%20million%20brownfield%20sites. Accessed 5 Apr 2021.

15. United States Environmental Protection Agency (US EPA). Brownfields Accomplishments. 2021. Available from: epa.gov/brownfields/brownfields-program-accomplishments-and-benefits. Accessed 5 Apr 2021.

16. California Department of Toxic Substances Control (DTSC). California Environmental Quality Act, Notice of Determination. 2019. Available from: envirostor.dtsc.ca.gov/public/view_document?docurl=/public/deliverable_documents/4368376016/20190806%20Final%20NOD%20Filed%2Epdf. Accessed 5 Apr 2021.

17. California Department of Toxic Substances Control (DTSC). DTSC's Response to the IRP's Request for Information Regarding CEQA Compliance and Human Health Risk Assessments in Permit Decisions. 2016. Available from: dtsc.ca.gov/wp-content/uploads/sites/31/2020/10/DTSC-Report-on-CEQA-Compliance-and-Human-Health-Risk-Assessments-in-Permitting-Decisions-October-14-2016_ADA.pdf. Accessed 5 Apr 2021.

18. Maro, A. Outsourcing the Filth: Privatizing Brownfield Remediation in New Jersey. B.C. Envtl. Aff. L. Rev. 2011, 38(1), 159-191.

19. United States Environmental Protection Agency (US EPA). Brownfields State and Tribal Response Program Grants. 2021. Available from: epa.gov/brownfields/state-and-tribal-response-program-grants. Accessed 5 Apr 2021.

20. United States Environmental Protection Agency (US EPA). Cleaning Up the Nation's Waste Sites. 2004. Available from: nepis.epa.gov/Exe/ZyNET.exe/30006II3.txt?ZyActionD=ZyDocument&Client=EPA&Index=2000%20Thru%202005&Docs=&Query=%28350000%29%20OR%20FNAME%3D%2230006II3.txt%22%20AND%20FNAME%3D%2230006II3.txt%22&Time=&EndTime=&SearchMethod=1&TocRestrict=n&Toc=&TocEntry=&QField=&QFieldYear=&QFieldMonth=&QFieldDay=&UseQField=&IntQFieldOp=0&ExtQFieldOp=0&XmlQuery=&File=D%3A%5CZYFILES%5CINDEX%20DATA%5C00THRU05%5CTXT%5C00000008%5C30006II3.txt&User=ANONYMOUS&Password=anonymous&SortMethod=h%7C-&MaximumDocuments=1&FuzzyDegree=0&ImageQuality=r75g8/r75g8/x150y150g16/i425&Display=hpfr&DefSeekPage=x&SearchBack=ZyActionL&Back=ZyActionS&BackDesc=Results%20page&MaximumPages=1&ZyEntry=10. Accessed 5 Apr 2021.

21. California Department of Toxic Substances Control (DTSC). Guidance for the Evaluation and Mitigation of Subsurface Vapor Intrusion to Indoor Air. 2011. Available from: dtsc.ca.gov/wp-content/uploads/sites/31/2018/01/Final_VIG_Oct_2011.pdf. Accessed 5 Apr 2021.

22. California Department of Toxic Substances Control (DTSC). Agreement and Covenant Not to Sue. 2011. Available from: envirostor.dtsc.ca.gov/public/view_document?docurl=/public/deliverable_documents/8377835929/Final%20PPA%20Pasadena%20Gateway%20PPA%2Epdf. Accessed 9 Aug 2021.

23. United States Environmental Protection Agency Office of Land and Emergency Management. Brownfields Site Information from SEMS as of the End of FY2019 from the 2015-2018 American Community Survey. 2020. Available from: epa.gov/sites/default/files/2015-09/documents/webpopulationbrownfieldsites.9.28.15.pdf. Accessed 13 Aug 2021.

24. United States Environmental Protection Agency Office of Land and Emergency Management. Superfund Site Information from SEMS as of the end of FY2019 from the 2015-2018 American Community Survey. Available from: epa.gov/sites/production/files/2015-09/documents/webpopulationrsuperfundsites9.28.15.pdf.

25. Savchenko, O.M.; Braden, J.B. Do Public Benefits of Voluntary Cleanup Programs Justify Their Public Costs? Evidence from New York. Land Economics. 2019, 95(3), 369-390. doi.org/10.3368/le.95.3.369.

26. Sigman, H.; Stafford, S. Management of Hazardous Waste and Contaminated Land. Annu Rev Resour Econ. 2011, 3(1), 255-275. doi.org/10.1146/annurev-resource-083110-120011.

27. Cernansky, R. It Came from Beneath: Detecting and Mitigating Vapor Intrusion. Environ Health Perspect. 2016, 124(4), a141-a146. doi.org/10.1289/ehp.124-A141.

28. United States Environmental Protection Agency (US EPA). Attachment A. In: 1998 Administrative Order on Consent Franklin Power Products, Inc and Amphenol Corporation. 2018. Available from: epa.gov/in/attachment-1998-administrative-order-consent-franklin-power-products-inc-and-amphenol-corporation. Accessed Accessed 5 Apr 2021.

29. Hamilton, J.W. Contamination at US Military Bases. Stanf Environ Law J. 2016, 35(2), 223-249.

30. United States Department of Health and Human Service Agency for Toxic Substances and Disease Registry Division of Community Health Investigations (ATSDR). Vapor Intrusion Public Health Assessment. 2020. Available from: atsdr.cdc.gov/sites/lejeune/Vapor-Intrusion-PHA.html. Accessed Accessed 5 Apr 2021.

31. Rolph, C.G.; Torres, V.E.; Everett, J.W. The "Volatile" World of Vapor Intrusion. Pace Environ Law Rev. 2012, 30, 107.

32. Fracassi, T.C.; Walsh, W.J. When May a Previously Approved Site Closure Not Provide Closure - Reopening Vapor Intrusion Sites Troutman Pepper. Troutman Pepper. 2016. Available from: lexology.com/library/detail.aspx?g=18a7c37c-fe12-41c9-9984-6d9fac765ad6. Accessed Accessed 5 Apr 2021.

33. Seifter, M. Rent-a-Regulator: Design and Innovation in Privatized Governmental Decision Making. Ecology Law Journal. 2006, 33, 1091-1148.

34. United States Environmental Protection Agency (US EPA). Weight of Evidence in Ecological Risk Assessment, EPA/100/R-16/001. 2016. Available from: nepis.epa.gov/Exe/ZyPURL.cgi?Dockey=P100SFXR.TXT. Accessed Accessed 5 Apr 2021.

35. Groeger, L.; GrochowskiJones, R.; Lustgarten, A. Bombs in Your Backyard. 2017. Available from: projects.propublica.org/bombs/. Accessed Accessed 5 Apr 2021.

36. Retail Compliance Center (RCC). Hazardous Waste Variations by State Matrix. 2020. Available from: rila.org/retail-compliance-center/hazardous-waste-variations-by-state. Accessed Accessed 5 Apr 2021.

37. United States Environmental Protection Agency (US EPA). National Priorities List Sites-by State. 2020. Available from: epa.gov/superfund/national-priorities-list-npl-sites-state. Accessed Accessed 5 Apr 2021.

38. Trickey, K.; Hadjimichael, N.; Sanghavi, P. Public Reporting of Hydraulic Fracturing Chemicals in the USA, 2011–18. Lancet Planet Health. 2020, 4(5), e178-e185. doi.org/10.1016/S2542-5196(20)30076-0.

39. Glicksman, R.L. Shuttered Government. Arizona Law Review. 2020, 62(3), 573-635.

40. Cox, B. The Future of Industrial Infill Development, The ICON Industrial Conference for Commercial Real Estate, National Association for Industrial and Office Parks. 2013. Available from: naiop.org/~/media/4EB2C472F63F408D96F43BE2DDEBFD9B.ashx. Accessed Accessed 5 Apr 2021.

41. United States Environmental Protection Agency (US EPA). Trichloroethylene; CASRN 79-01-6. 2011. Available from: cfpub.epa.gov/ncea/iris/iris_documents/documents/subst/0199_summary.pdf#nameddest=canceroral. Accessed 30 Aug 2020.

42. United States Environmental Protection Agency (US EPA). Compilation of Information Relating to Early/Interim Actions at Superfund Sites and the TCE IRIS Assessment. 2014. Available from: semspub.epa.gov/work/HQ/174044.pdf. Accessed Accessed 5 Apr 2021.

43. California Department of Toxic Substances Control (DTSC). Project Search Results. 2021. Available from: envirostor.dtsc.ca.gov/public/search?CMD=search&ocieerp=&HWMP=False&business_name=&main_street_name=&city=&zip=&county=&censustract=&case_number=&apn=&CRITICAL_POL=30027&Search=Get+Report. Accessed 9 Aug 2021.

44. Kripke, Saul. Wittgenstein on Rules and Private Language. 1982. Harvard University Press: Cambridge.

45. California Department of Toxic Substances Control (DTSC), California Water Resources Control Boards. Supplemental Guidance: Screening and Evaluating Vapor Intrusion, Draft for Public Comments. 2020. Available from: dtsc.ca.gov/wp-content/uploads/sites/31/2020/02/Public-Draft-Supplemental-VI-Guidance_2020-02-14.pdf. Accessed 30 Jan 2021.

46. United States Environmental Protection Agency (US EPA). OSWER Technical Guide for Assessing and Mitigating the Vapor Intrusion Pathway from Subsurface Vapor Sources to Indoor Air. 2015. Available from: epa.gov/sites/production/files/2015-09/documents/oswer-vapor-intrusion-technical-guide-final.pdf. Accessed Accessed 5 Apr 2021.

47. United States Environmental Protection Agency (US EPA). Technical Guide for Addressing Petroleum Vapor Intrusion. 2015. Available from: epa.gov/sites/production/files/2015-06/documents/pvi-guide-final-6-10-15.pdf. Accessed 5 Apr 2021.

48. California Department of Toxic Substances Control (DTSC), Los Angeles Regional Water Quality Control Board (LARWQCB), San Francisco Regional Water Quality Control Board (SFRWQCB). Advisory: Active Soil Gas Investigations. 2015. Available from: dtsc.ca.gov/wp-content/uploads/sites/31/2018/01/VI_ActiveSoilGasAdvisory_FINAL.pdf. Accessed 5 Apr 2021.

49. California Department of Toxic Substances Control (DTSC). Proven Technologies and Remedies Guidance: Remediation of Chlorinated Volatile Organic Compounds in Vadose Zone Soil. 2010. Available from: dtsc.ca.gov/wp-content/uploads/sites/31/2018/11/cVOC_040110.pdf. Accessed Accessed 5 Apr 2021.

50. California Department of Toxic Substances Control, Human and Ecological Risk Office (DTSC-HERO). Human Health Risk Assessment Note #4. 2019. Available from: dtsc.ca.gov/wp-content/uploads/sites/31/2019/05/HHRA-Note-Number-4-May-14-2019.pdf. Accessed Accessed 5 Apr 2021.

51. California Department of Toxic Substances Control (DTSC). Naval Information Research Foundation (19970020). 2020. Available from: envirostor.dtsc.ca.gov/public/profile_report?global_id=19970020. Accessed Accessed 5 Apr 2021.

52. California Department of Toxic Substances Control (DTSC). Alexan Monrovia (60002828). 2020. Available from: envirostor.dtsc.ca.gov/public/profile_report?global_id=60002828. Accessed 30 Aug 2021.

53. California Department of Toxic Substances Control (DTSC). Raytheon Systems Co (CAD041162124). 2020. Available from: envirostor.dtsc.ca.gov/public/hwmp_profile_report?global_id=CAD041162124&starttab=. Accessed 30 Aug 2020.

54. California Department of Toxic Substances Control (DTSC). Branford Landfill (19990021). 2020. Available from: envirostor.dtsc.ca.gov/public/profile_report?global_id=19990021. Accessed 30 Aug 2021.

55. California Department of Toxic Substances Control (DTSC). Crown Coach (19400008). 2020. Available from: envirostor.dtsc.ca.gov/public/profile_report?global_id=19400008. Accessed 30 Aug 2021.

56. National Research Council. Contaminants in the Subsurface. 2005. Available from: nap.edu/read/11146/chapter/1. Accessed 13 Aug 2021.

57. City of Pasadena Planning & Community Development (CPPCD). Sustainable Communities Environmental Assessment. 2018. Available from: ww5.cityofpasadena.net/planning/wp-content/uploads/sites/56/2018/01/3200-E-Foothill-SCEA.pdf. Accessed 30 Aug 2021.

58. Ninyo and Moore. Removal Action Workplan: Former Naval Information Research Foundation Undersea Center. 2019. Available from: envirostor.dtsc.ca.gov/public/deliverable_documents/1932116448/Revised%20RAW%2012.12.2017.PDF. Accessed 30 Aug 2021.

59. Ninyo and Moore. Appendix D of Remedial Investigation and Feasibility Study: Former Naval Information Research Foundation Undersea Center. 2017. Available from: envirostor.dtsc.ca.gov/public/deliverable_documents/3954603433/Final%20RI.FS%20report11.2018.pdf. Accessed 30 August 2021.

60. California Department of Toxic Substances Control (DTSC). Human Health Risk Assessment (HHRA) Note Number 3, DTSC-Modified Screening Levels (DTSC-SLs). 2020. Available from: dtsc.ca.gov/wp-content/uploads/sites/31/2019/04/HHRA-Note-3-June-2020-A.pdf. Accessed 30 Jan 2021.

61. Frey Environmental Inc. Preliminary Endangerment Assessment Workplan, Alexan Monrovia Project, Southwest Corner of South Magnolia & West Evergreen Avenues (340, 410, 418 & 494 West Evergreen Avenue, 1607, 1621, 1625, 1641 & 1671 South Magnolia Avenue & 1541 South Dale Drive), Monrovia, California 91016. 2020. Available from: envirostor.dtsc.ca.gov/public/deliverable_documents/2288649618/FinalPEAWorkplan_AlexanMonrovia_02262020.pdf. Accessed 30 Aug 2021.

62. Partner Engineering and Science Inc. Appendix E of Phase II Environmental Assessment Report. 2011. Available from: cityofmonrovia.org/home/showdocument?id=21200. Accessed 30 Aug 2021.

63. United States Environmental Protection Agency (US EPA). Regional Screening Levels (RSLs) – Generic Tables. 2020. Available from: https://www.epa.gov/risk/regional-screening-levels-rsls-generic-tables. Accessed 5 Apr 2021. Accessed 13 Aug 2021.

64. Los Angeles City Planning Commission (LACPC). Corrected Determination: Corporate Pointe; Chatsworth-Porter Ranch. 2009. Available from: clkrep.lacity.org/onlinedocs/2009/09-1510_rpt_cpc_6-15-09.pdf. Accessed 30 Aug 2021.

65. California Department of Toxic Substances Control (DTSC). Raytheon Systems Co (80001366). 2020. Available from: envirostor.dtsc.ca.gov/public/profile_report?global_id=80001366. Accessed 30 Aug 2021.

66. Holdridge, N. Neal Holdridge (Trammell Crow) to Martin Herrmann (DTSC), West Hills Campus at 8413 Fallbrook Avenue, May 5, 2008. 2008. Available from: envirostor.dtsc.ca.gov/public/view_document?docurl=/public/deliverable_documents/1158131241/2008%2D0508%20SMP%20dtsc%20approval%20letter%20MEPT%20%2DEnvirostor%20mod%2Epdf. Accessed 30 Aug 2021.

67. Los Angeles Regional Water Quality Control Board (LARWQCB). No Further Action for Groundwater. 2018. Available from: documents.geotracker.waterboards.ca.gov/regulators/deliverable_documents/9192200183/0693A.%20No%20Further%20Action%20For%20Groundwater_3-22-2018.pdf. Accessed 30 Aug 2021.

68. Unger, S. Los Angeles Regional Water Quality Control Board, Order No. R4-2011-0194, Postclosure Maintenance, Waste Discharge Requirements for Scannell Properties #127, LLC, Branford Landfill, File No. 56-101. 2011. Available from: documents.geotracker.waterboards.ca.gov/regulators/deliverable_documents/4513079583/FN%2056-101%20%232%20(Branford%20Landfill).pdf. Accessed 30 Aug 2021.

69. Chiang, K. Branford Landfill Site EPA Region IX Site Screening Checklist. 1997. Available from: envirostor.dtsc.ca.gov/public/view_document?docurl=/public/deliverable_documents/6312543570/BRANFORD%20LANDFILL%2Epdf. Accessed 30 Aug 2021.

70. Dickerson, D.A. Los Angeles Regional Water Quality Control Board Order No. R4-2011-0194, Postclosure Maintenance, Waste Discharge Requirements for Scannell Properties #127, LLC, Branford Landfill, File No. 56-101. 2012. Available from: documents.geotracker.waterboards.ca.gov/regulators/deliverable_documents/4513079583/FN%2056-101%20%232%20(Branford%20Landfill).pdf. Accessed 30 Aug 2021.

71. California Integrated Water Quality System (CIWQS). Violations with/without Enforcement Report. 2020. Available from: ciwqs.waterboards.ca.gov/ciwqs/readOnly/ciwqsReportEnforcement.jsp?reportID=2&place=&sortOrder=AGENCY&sortDir=DESC&printerFriendly=Y. Accessed 30 Aug 2021.

72. Oneida Total Integrated Enterprises. Confirmation Sampling Report for No Further Action Status in the Southern Parcels. 2012. Available from: documents.geotracker.waterboards.ca.gov/esi/uploads/geo_report/7808011889/SL2043T1572.PDF. Accessed 30 Aug 2021.

73. California Department of Toxic Substances Control (DTSC). Raytheon-Canoga Park - DTSC Comments. 2016. Available from: envirostor.dtsc.ca.gov/public/deliverable_documents/2647779703/2016-09-02-Raytheon-Canoga-historical%20reports-DTSC%20Comments.pdf. Accessed 30 Aug 2021.

74. Tsiatsios, C. Christopher Tsiatsios of Haley and Aldrich to Los Angeles Regional Water Quality Control Board, Re Branford Monitoring and Reporting. 2012. Available from: documents.geotracker.waterboards.ca.gov/regulators/deliverable_documents/4513079583/FN%2056-101%20%232%20(Branford%20Landfill).pdf. Accessed 30 Aug 2021.

75. California Department of Toxic Substances Control (DTSC). DTSC Comments; Raytheon Canoga Park Site - 8433 Fallbrook Avenue, Canoga Park, CA 91304. 2017. Available from: envirostor.dtsc.ca.gov/public/deliverable_documents/2595278680/2017-12-15-Raytheon-DTSC_Ltr_SouthernParcels.pdf. Accessed 30 Aug 2021.

76. Fluor Daniel Groundwater Technology Incorporated (GTI). Appendix A: Historical Soil Gas Data. 1998. Available from: documents.geotracker.waterboards.ca.gov/esi/uploads/geo_report/7808011889/SL2043T1572.PDF. Accessed 30 Aug 2021.

77. Environ. Interim Results Report, Soil Sampling and Monitoring; Construction of Café Area. 2008. Available from: envirostor.dtsc.ca.gov/public/deliverable_documents/7398267447/2008-0718%20West%20Hills%20Cafe%20Area%20Interim%20Results%20Report%20final.pdf . Accessed 30 Aug 2021.

78. State Water Resources Control Board (SWRCB). Branford Landfill (L10002785228). 2020. Available from: geotracker.waterboards.ca.gov/profile_report?global_id=L10002785228. Accessed 30 Aug 2021.

79. EnSafe Inc. Final Remedial Action Closure Report CleanTech Manufacturing Center 2425 Washington Boulevard, Los Angeles, California. 2018. Available from: envirostor.dtsc.ca.gov/public/deliverable_documents/7772862076/2018-08-24%20Final%20Remedial%20Action%20Completion%20Report.pdf. Accessed 30 Aug 2021.

80. California Department of Toxic Substances Control (DTSC) and LACRA. Exhibit B: Property Description of Project Environmental Oversight Agreement for the Crown Coach Industrial Redevelopment Project. 2007. Available from: envirostor.dtsc.ca.gov/public/deliverable_documents/9058850944/fully%20executed.pdf. Accessed 30 Aug 2021.

81. California Department of Toxic Substances Control (DTSC). Removal Action Certification Form. 2019. Available from: envirostor.dtsc.ca.gov/public/final_documents2?global_id=19400008&enforcement_id=60429106. Accessed 30 August 2021.

82. United States Environmental Protection Agency (US EPA). Regional Screening Level Composite Worker Ambient Air Table (TR=1E-06, HQ=1) May 2016. 2016. Available from: 19january2017snapshot.epa.gov/sites/production/files/2016-06/documents/indair_sl_table_run_may2016.pdf. Accessed 30 Aug 2021.

83. Frey Environmental Inc. Appendix G1; Phase I Environmental Assessment Report. 2017. Available from: cityofmonrovia.org/home/showdocument?id=21200. Accessed 30 Aug 2021.

84. Shrader-Frechette, K.; Biondo, A.M. Data-Quality Assessment Signals Toxic-Site Safety Threats and Environmental Injustice. Int J Environ Res Public Health. 2021, 18(4), 2012. doi.org/10.3390/ijerph18042012.

85. Shrader-Frechette, K.; Meade, T. Using Routine, Independent, Scientific-Data Audits as an Early-Warning for Potentially Fraudulent Toxic-Site Cleanup. Account Res. 2020, 27(1), 1-31. doi.org/10.1080/08989621.2019.1695200.

86. Shrader-Frechette, K.; Biondo, A.M. Protecting Children from Toxic Waste: Data-Usability Evaluation Can Deter Flawed Cleanup. Int J Environ Res Public Health. 2020, 17(2), 424. doi.org/10.3390/ijerph17020424.

87. Scandura, J.E. Imminent and Substantial Endangerment Determination and Remedial Action Order, I/SE-ROA 04/05-00. Cypress: California Department of Toxic Substances Control; 2004.

88. California Department of Toxic Substances Control (DTSC). Agreement and Covenant Not to Sue. 2011. Available from: envirostor.dtsc.ca.gov/public/deliverable_documents/8377835929/Final%20PPA%20Pasadena%20Gateway%20PPA.pdf. Accessed 30 Aug 2021.

89. California Department of Toxic Substances Control (DTSC). Amendment to Agreement and Covenant Not to Sue. 2017. Available from: envirostor.dtsc.ca.gov/public/final_documents2?global_id=19970020&enforcement_id=60406047. Accessed 30 Aug 2021.

90. Shrader-Frechette, K.; Biondo, A.M. Health Misinformation about Toxic-Site Harm: The Case for Independent-Party Testing to Confirm Safety. Int J Environ Res Public Health. 2021, *18*(8), 3882; doi.org/10.3390/ijerph18083882.

91. Holdridge, N. Holdridge, Neal H. (Trammell Crow), to City Council of Pasadena Re: 3200 E. Foothill Boulevard/Item No. 14 on the Council's April 29 Agenda, April 29, 2019. 2019. Available from: ww2.cityofpasadena.net/councilagendas/2019%20Agendastable/Apr_29_19/AR%2014%20SUPPLEMENTAL%20CORRESPONDENCE.pdf. Accessed 30 Aug 2021.

92. California Office of Senate Floor Analyses, Senate Bill 158: Hazardous Waste, Analysis, 2021. Available from: leginfo.legislature.ca.gov/faces/billAnalysisClient.xhtml?bill_id=202120220SB158#. Accessed 12 Aug 2021.

93. Johnston, J.E.; Hricko, A. Industrial Lead Poisoning in Los Angeles. Environ Justice. 2017;10(5):162–167. doi.org/10.1089/env.2017.0019.

94. California Department of Toxic Substances Control (DTSC), Williams M. DTSC Imminent and Substantial Endangerment Determination Tied to Exide Bankruptcy. 2021. Available from: dtsc.ca.gov/2020/10/12/dtsc-imminent-and-substantial-endangerment-determination-tied-to-exide-bankruptcy/. Accessed 5 Apr 2021.

95. California Department of Toxic Substances Control (DTSC). Response to Public Comments: 3200 East Foothill Boulevard, Pasadena. Cypress: DTSC; 2019.

96. California Environmental Protection Agency (CAL-EPA). Los Angeles Initiative Report. 2017. Available from: calepa.ca.gov/wp-content/uploads/sites/6/2017/02/LAReport.pdf. Accessed 18 Oct 2021.

97. California Office of Environmental Health Hazard Assessment (OEHHA). CalEnviroScreen 3.0 Results. 2018. Available from: oehha.maps.arcgis.com/apps/webappviewer/index.html?id=4560cfbce7c745c299b2d0cbb07044f5. Accessed 30 Aug 2021.

98. City of Los Angeles Department of City Planning. Boyle Heights Demographic Profile. 2019. Available from: planning.lacity.org/odocument/f09e7f04-93d6-4a5b-9aa4-0cf428397a02/2017_demo_profile_boyle_hts.pdf. Accessed 30 Nov 2021.

99. Statistical Atlas. Age and Sex in Pacoima, Los Angeles, California. 2018. Available from: statisticalatlas.com/neighborhood/California/Los-Angeles/Pacoima/Age-and-Sex. Accessed 30 Nov 2020.

100. United States Environmental Protection Agency (US EPA). Environmental Justice. 2016. Available from: archive.epa.gov/socal/web/html/justice.html. Accessed 18 Oct 2021.

101. Statistical Atlas. Age and Sex in Canoga Park, Los Angeles, California. 2018. Available from: statisticalatlas.com/neighborhood/California/Los-Angeles/Canoga-Park/Race-and-Ethnicity. Accessed 30 Nov 2021.

102. Cohen, Y.; Harmon, T.C.; Glik, D.; Katner, A.; Grifoll, J.; Wheeler, N.; Chinkin, L.; Ryan, P. The Potential for Offsite Exposures Associated with Santa Susana Field Laboratory, Ventura County, California. UCLA Center for Environmental Risk Reduction. 2006. Available from: https://www.dtsc-ssfl.com/files/lib_offsite_investig/bbi/reports/UCLA-2006-Cohen-UCLA-2006-02-02.pdf. Accessed 15 Oct 2021.

103. California Environmental Protection Agency (CAL-EPA). Report of the Santa Susana Field Laboratory Advisory Panel. 2006. Available from: ssflpanel.org/files/SSFLPanelReport.pdf. Accessed 15 Oct 2021.

104. Traffic Data Branch of California State Transportation Agency Department of Transportation. Annual Average Daily Truck Traffic on the California State Highway System. 2016. Available from: dot.ca.gov/-/media/dot-media/programs/traffic-operations/documents/census/f0017681-2016-aadt-truck-a11y.pdf. Accessed 30 Nov 2021.

105. Statistical Atlas. Household Types in California. 2018. Available from: statisticalatlas.com/state/California/Household-Types. Accessed 30 Nov 2021.

106. Trammell Crow Residential (TCR); Moore, Iacofano, & Goltsman Inc (MIG). Planned Development General Plan Amendment, Zoning Code Amendment, & Alexan Foothills Specific Plan. 2019. Available from: ceqanet.opr.ca.gov/2018101058/3/Attachment/7b0RIk. Accessed 13 Aug 2021.

107. California Air Resources Board (CARB). Air Quality and Land Use Handbook. 2005. Available from: arb.ca.gov/ch/landuse.htm. Accessed 18 Oct 2021.

108. US Environmental Protection Agency. National Priorities List and Superfund Alternative Approach Site. 2022. Available from: epa.gov/superfund/search-superfund-sites-where-you-live#advanced. Accessed 3 March 2022

109. Massachusetts Office of Energy and Environmental Affairs. Data Portal, 2022. Available from: eeaonline.eea.state.ma.us/Portal/#!/search/wastesite . Accessed 8 March 2022.

110. New Jersey Department of Environmental Protection. Site Remediation Program. 2022. Available from: https://www.nj.gov/dep/srp/brownfields/ . Accessed 6 March 2022.

111. Geosyntec Consultants. Vapor Intrusion Guidance by State. 2022. Available from:

geosyntec.com/vapor-intrusion-guidance. Accessed 8 March 2022.

112. McHugh, T.; Loll,P.; Eklund, B. Recent advances in vapor intrusion site investigations. Journal of Environmental Management. 2017, 204(2), 783-792. doi.org/10.1016/j.jenvman.2017.02.015 . Accessed 8 March 2022.

113. Office of the Inspector General. US Environmental Protection Agency. Fiscal Year 2022 Oversight Plan. epa.gov/system/files/documents/2021-12/_epaoig_20211214_oig_fiscal_year_2022_oversight_plan.pdf . Accessed 2 March 2022.

114. US Government Accountability Office. Environmental Auditing: A Useful Tool That Can Improve Environmental Performance and Reduce Costs. GAO/RCED-95-37. 1995. Available from: govinfo.gov/content/pkg/GAOREPORTS-RCED-95-37/html/GAOREPORTS-RCED-95-37.htm . Accessed 8 Oct 2021.

115. Shamoo, A.E.; Annau, Z. Ensuring Scientific Integrity. Nature. 1987, 327(6123), 550. doi.org/10.1038/327550b0.

116. Electronic Code of Federal Regulations. Scientific Integrity. CFR 1552.203-72. 2021. Available from: https://www.ecfr.gov/cgi-bin/text-idx?SID=2483538e46c4021c1f1e86a058fea4ff&mc=true&node=se48.6.1552_1203_672&rgn=div8. Accessed 18 Apr 2021.

117. Gorman, S. California Bans Private Prisons and Immigration Detention Centers. 2019. Available from: reuters.com/article/us-california-prisons/california-bans-private-prisons-and-immigration-detention-centers-idUSKBN1WQ2Q9. Accessed 13 Aug 2021.

118. United States Environmental Protection Agency (US EPA). Information on Sites Eligible for Brownfields Funding under CERCLA §104(k). 2017. Available from: epa.gov/sites/production/files/2018-10/documents/web-content-info-on-site-eligibility.pdf. Accessed 5 Apr 2021.

119. United States Environmental Protection Agency (US EPA). RCRA Online. 2020. Available from: rcrapublic.epa.gov/rcraonline/. Accessed 10 Apr 2021.

120. United States Environmental Protection Agency (US EPA). The Environmental Challenge of Military Munitions and Federal Facilities. 2020. Available from: epa.gov/enforcement/environmental-challenge-military-munitions-and-federal-facilities. Accessed 5 Apr 2021.

121. United States Government Accountability Office (GAO). Hazardous Waste Cleanup, GAO-15-830T. 2015. Available from: gao.gov/assets/680/672464.pdf. Accessed 5 Apr 2021..

122. Pintas and Mullins Law Firm. How Many Military Sites are Superfund Sites? 2021. Available from: pintas.com/pfas-class-action-lawsuit/how-many-military-sites-are-superfund-sites/. Accessed 5 Apr 2021.

123. United States Department of Energy (US DOE). Cleanup Sites. 2021. Available from: energy.gov/em/mission/cleanup-sites. Accessed 5 Apr 2021.

124. Colbert, K.L.; Palazzolo, J.E. Vapor Intrusion: Liability Determination Protects Profits and Minimizes Risk. Real Estate Finance. 2008, 24, 17-22.

125. Cosier, S. With Dozens of Sick Children, Parents Took a Hard Look at Their Town's Toxic Legacy. Earth Island Journal. 2019. Available from: earthisland.org/journal/index.php/articles/entry/sick-children-cancer-parents-indiana-towns-toxic-legacy/. Accessed 5 Apr 2021.

126. Colorado Department of Public Health and Environment. Analysis of Diagnosed Vs. Expected Cancer Cases in the Vicinity of the Redfield Plume Area in Southeast Denver County, 1979–1999. Denver: Colorado Department of Public Health and Environment, Disease Control & Environmental Epidemiology Division; 2002.

127. World Health Organization (WHO). WHO/HEP/ECH/WSH/2020.10, Trichloroethene in Drinking-Water, Background Document for Development of WHO Guidelines for Drinking-Water Quality. 2020. Available from: apps.who.int/iris/bitstream/handle/10665/338071/WHO-HEP-ECH-WSH-2020.10-eng.pdf. Accessed 5 Apr 2021.

128. Kennedy-Jenks Consultants. Environmental Summary Report: Former Naval Information Research Foundation Space/Site Bank. 2007. Available from: envirostor.dtsc.ca.gov/public/deliverable_documents/4649860978/Environmental%20Summary%20Report%20Jenks%20May%2022%202007%20.pdf. Accessed 5 Apr 2021..

129. United States Environmental Protection Agency (US EPA). Brownfields Voluntary Agreements. 2020. Available from: epa.gov/brownfields/brownfields-voluntary-agreements#:~:text=State%20voluntary%20cleanup%20programs%20(VCP,regarding%20the%20cleanup%20of%20sites. Accessed 5 Apr 2021.

130. Bălan, S.A.; Mathrani, V.C.; Guo, D.F.; Algazi, A.M. Regulating PFAS as a Chemical Class under the California Safer Consumer Products Program. Environ Health Perspect. 2021, 129(2), 025001. doi.org10.1289/EHP7431.

131. United States Environmental Protection Agency (US EPA). Status of EPA Research and Development on PFAS. 2021. Available from: epa.gov/chemical-research/status-epa-research-and-development-pfas. Accessed 5 Apr 2021.

132. Johnston, J.; MacDonaldGibson, J. Indoor Air Contamination from Hazardous Waste Sites. Int. J. Environ. Res. Public Health. 2015, 12(12), 15040-57. doi.org/10.3390/ijerph121214960.

133. Burk, T.; Zarus, G. Community Exposures to Chemicals through Vapor Intrusion. J. Environ. Health. 2013, 75(9), 36–41.

134. United States Government Accountability Office (US GAO). Hardrock Mining: Information on Abandoned Mines, GAO-08-574T. 2008. Available from: govinfo.gov/content/pkg/GAOREPORTS-GAO-08-574T/html/GAOREPORTS-GAO-08-574T.htm. Accessed 14 Apr 2021.

135. United States Government Accountability Office (GAO). Environmental Cleanup: Too Many High Priority Sites Impede DOD's Program, GAO/NSIAD-94-133. 1994. Available from: govinfo.gov/content/pkg/GAOREPORTS-NSIAD-94-133/html/GAOREPORTS-NSIAD-94-133.htm. Accessed 30 Jan 2021.

136. Lustgarten, A. How Military Outsourcing Turned Toxic. ProPublica. 2017. Available from: features.propublica.org/military-pollution/military-pollution-contractors-scandal-toxic-cleanups/. Accessed 5 Apr 2021.

137. Mullaney, R.D. CERCLA Cleanup, 2019.09.06: Comment on Navy FYR Addendum on Soil, Environmental Law and Justice Clinic, Golden Gate University School of Law. 2019 Available from: digitalcommons.law.ggu.edu/cgi/viewcontent.cgi?article=1000&context=hpns. Accessed 13 Aug 2021.

138. *PorterSumchai, A.* Brain Cancer Biomonitoring in Bayview Hunter's Point. San Francisco Bay View National Black Newspaper. 2021. Available from: sfbayview.com/2021/03/brain-cancer-biomonitoring-in-bayview-hunters-point/. Accessed 13 Aug 2021.

139. Hirsch, D.; Caine, M.; Altenbern, T.; Williams, H.; Ford, A. From Cleanup to Coverup. Committee to Bridge the Gap. 2019. Available from: committeetobridgethegap.org/hunters-point-reports/FromCleanupToCoverup.pdf. Accessed 5 Apr 2021.

140. Gibbs Law Group. Hunters Point Lawsuit 2021 SF Shipyards Lawsuit. 2021. Available from: classlawgroup.com/hunters-point-contamination-lawsuit/. Accessed 5 Apr 2021.

141. Environmental Litigation Committee of the American Bar Association (ELC-ABA). 50-State Survey of Protections Available for Purchasers of Contaminated Property. 2014. Available from: nelsonmullins.com/storage/6027b7d9d8e6105166c31130d10292b8.pdf. Accessed 5 Apr 2021.

142. California Department of Toxic Substances Control (DTSC). Standard Voluntary Agreement Model. 2020. Available from: dtsc.ca.gov/wp-content/uploads/sites/31/2019/04/Standard-Voluntary-Agreement-Model.pdf. Accessed 5 Apr 2021.

143. New Jersey Department of Environmental Protections (NJDEP). New Jersey's Contaminated-site Cleanup Program Hits Milestone. 2017. Available from: nj.gov/dep/newsrel/2017/17_0069.htm. Accessed 5 Apr 2021.

144. United States Environmental Protection Agency (US EPA). Addressing Environmental Justice in EPA Brownfields Communities. 2009. Available from: epa.gov/brownfields/addressing-environmental-justice-epa-brownfields-communities. Accessed 13 Aug 2021.

145. Yacovone, K. Brownfields and the Poor: Is Cleanup a Hazardous Waste of Time? Fordham Int. Law J. 2016, 35, 201-47.

146. Faber, D.R.; Krieg, E.J. Unequal Exposure to Ecological Hazards: Environmental Injustices in the Commonwealth of Massachusetts. Environ Health Perspect. 2002, 110(Suppl 2), 277–288. doi.org/10.1289/ehp.02110s2277.

147. Engel, K.H. Brownfield Initiatives and Environmental Justice? J. Energy Nat. Resour. Law. 1997, 13(2), 317–338.

148. McCarthy, L. Off the Mark?: Efficiency in Targeting the Most Marketable Sites Rather than Equity in Public Assistance for Brownfield Redevelopment. Econ. Dev. Q. 2009, 23(3), 211–228. doi.org/10.1177/0891242408331159.

149. Haninger, K.; Ma, L.; Timmins, C. The Value of Brownfield Remediation. J Assoc Environ Resour Econ. 2017, 4(1), 197-241. doi.org/10.1086/689743.

150. Essoka, J.D. The Gentrifying Effects of Brownfields Redevelopment. West J Black Stud. 2010, 34(3), 299–315.

151. Lee, S.; Mohai, P. Environmental Justice Implications of Brownfield Redevelopment in the United States. Soc Nat Resour. 2012, 25(6), 602-609. doi.org/10.1080/08941920.2011.566600.

152. State of California. About CEQAnet. 2021. Available from: ceqanet.opr.ca.gov/Home/About. Accessed 5 Apr 2021.

153. State of California. State Clearinghouse. 2021. Available from: opr.ca.gov/clearinghouse/ceqa. Accessed 5 Apr 2021.

154. Trammell Crow Company (TCC). Environmental Assets Services International (EASI). 2020. Available from: trammellcrow.com/-/media/tcc_hsr/countrytcc/files/brochures/corp_mm_easi.pdf. Accessed 5 Apr 2021.

155. Trammel Crow Company (TCC). Robert Chute. 2020. Available from: trammellcrow.com/en/people-and-offices/robert-chute. Accessed 5 Apr 2021.

156. United States Environmental Protection Agency (US EPA). Resource Conservation and Recovery Act (RCRA) Overview. 2020. Available from: epa.gov/rcra/resource-conservation-and-recovery-act-rcra-overview. Accessed 5 Apr 2021..

157. United States Environmental Protection Agency (US EPA). Guidelines for Carcinogen Risk Assessment. EPA/630/P-03/001F. 2005. Available from: epa.gov/sites/default/files/2013-09/documents/cancer_guidelines_final_3-25-05.pdf. Accessed 5 Apr 2021.

158. United States Environmental Protection Agency (US EPA). 540/R-95/141 Superfund Program Representative Sampling Guidance Volume 4: Waste. 2016. Available from: epa.gov/sites/production/files/2016-03/documents/superfund-samp.pdf. Accessed 5 Apr 2021.
